# Human Infection with Orf Virus and Description of Its Whole Genome, France, 2017

**DOI:** 10.3201/eid2512.181513

**Published:** 2019-12

**Authors:** Julien Andreani, Jessica Fongue, Jacques Y. Bou Khalil, Laurene David, Saïd Mougari, Marion Le Bideau, Jonatas Abrahão, Philippe Berbis, Bernard La Scola

**Affiliations:** Institut Hospitalo-Universitaire Méditerranée Infection, Marseille, France (J. Andreani, J.Y. Bou Khalil, S. Mougari, M. Le Bideau, B. La Scola);; Centre Hospitalier Universitaire Hôpital Nord, Marseille (J. Fongue, L. David, P. Berbis);; Universidade Federal de Minas Gerais, Belo Horizonte, Brazil (J. Abrahão)

**Keywords:** orf virus, viruses, poxviridae, human infection, Aïd-el-Fitr, festival, case report, whole-genome sequencing, phylogenetic analysis, ecthyma contagiosum, sheep, France

## Abstract

Zoonotic transmission of parapoxvirus from animals to humans has been reported; clinical manifestations are skin lesions on the fingers and hands after contact with infected animals. We report a human infection clinically suspected as being ecthyma contagiosum. The patient, a 65-year-old woman, had 3 nodules on her hands. She reported contact with a sheep during the Aïd-el-Fitr festival in France during 2017. We isolated the parapoxvirus orf virus from these nodules by using a nonconventional cell and sequenced the orf genome. We identified a novel orf virus genome and compared it with genomes of other orf viruses. More research is needed on the genus *Parapoxvirus* to understand worldwide distribution of and infection by orf virus, especially transmission between goats and sheep.

*Parapoxvirus* is a genus of double-stranded DNA viruses (family *Poxviridae*) that contains 4 virus species: orf virus, bovine papular stomatitis virus, parapoxvirus of red deer, and pseudocowpoxvirus. Recently, a complete genome from a gray seal infected by a parapoxvirus was reported and constituted a putative novel virus in this genus (*1*). Zoonotic transmission of parapoxvirus from animals to humans has been reported in the past few decades; the main human clinical manifestations are skin lesions on the fingers and hands after contact with infected animals (*2*–*5*).

Most human cases of infection with parapoxvirus reported are caused by orf virus (*2*,*6*,*7*), but some human infections are caused by pseudocowpoxviruses (*8*,*9*). Infection of small ruminants with orf virus is frequent and widely distributed worldwide. Orf virus disease is also known as contagious ecthyma, scabby mouth, sore mouth, or infectious pustular dermatitis. Humans can be infected with orf virus by contact with sheep and goats during religious or cultural practices and during slaughter of animals (*4*,*10*), and infections appear to be more frequent during the last 3 months of each year (*3*). Human-to-human infection is extremely rare (*11*,*12*). Vaccination against orf virus is available for animals, although it does not confer long-term protective immunity (*13*). Human infections are relatively frequent when populations are exposed to sheep and goats (occupational disease). However, complete genomes of orf viruses are rarely found in public databases, which results in limited comparative studies in this field.

Diagnosis of human infections with orf virus is usually made by histologic analysis, molecular biology (PCR) studies, or electron microscopy. At least 11 complete orf virus genomes are available, and at least 19 are available for the entire genus *Parapoxvirus*. Nevertheless, a unique orf virus was sequenced after a human case report (*14*) (orf virus strain B029). These data are in contrast with those for orthopoxviruses (another *Poxviridae* genus) in which >300 genomes are available. One of the reasons for limited availability of parapoxvirus genomes is that many of these viruses are not cultivable on most diagnostic laboratories, cell lines (*15*).

In 2017, we identified a 65-year-old woman in France who had 3 nodules on her hands. She was given a diagnosis of ecthyma contagiosum. Genomic and electron microscopy data confirmed the initial diagnosis as infection with orf virus and identified this virus as the etiologic agent. We also isolated this virus on OA3.Ts cells.

## Materials and Methods

### Case-Patient

**Figure 1:**
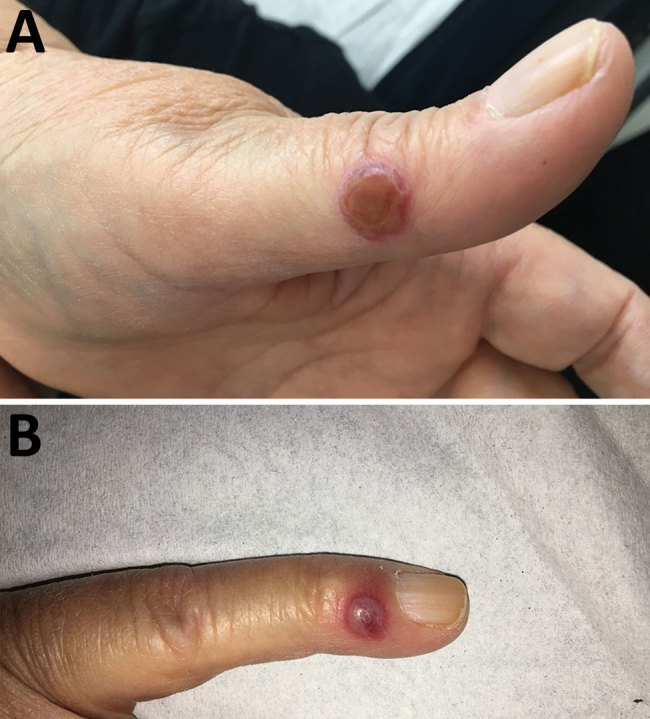
Nodules on the A) left thumb and B) left little finger of a 65-year-old woman infected with orf virus during Aïd-el-Fitr festival, France, 2017.

**Figure 2:**
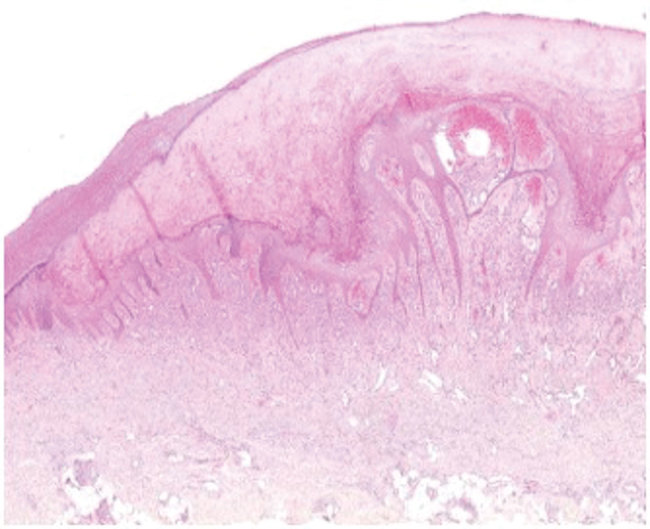
Histopathologic analysis of a skin biopsy specimen from a 65-year-old woman infected with orf virus during Aïd-el-Fitr festival, France, 2017. The specimen shows epidermal hyperplasia with acantolysis and papillomatosis, extensive hyperkeratosis, spongiform degeneration and vacuolated cells, and inflammatory infiltration in the dermis, predominantly by histiocytes and lymphocytes. Hematoxylin and eosin stain, original magnification x100.

A 65-year-old woman came to North Hospital (Marseille, France) because of 3 nodules on her hands. She reported contact 3 weeks earlier with the carcass of a dead sheep during the Aïd-el-Fitr festival (June 25, 2017) in the Bouches-du-Rhones Department in southern France. Clinically, she had 3 painless, well-delimited erythematosus nodules on her fingers with an erythematosus halo (Figure 1). On the basis of clinical suspicion of ecthyma contagiosum, we obtained a cutaneous biopsy specimen for biologic confirmations by PCR and histologic analysis. Histopathologic analysis of the skin biopsy specimen showed a moderate epidermal hyperplasia, spongiform degeneration with vacuolated cells, and inflammatory infiltration into the dermis (Figure 2). The patient was given antiseptic and local antimicrobial drug therapy (2% fusidic acid cream) to prevent bacterial superinfection. All skin lesions healed in 3 weeks.

### Virus Detection, Isolation, and Production

We performed a parapoxvirus PCR on the cutaneous biopsy sample by using primers forward 5′-CGGTGCAGCACGAGGTC-3′, reverse 5′- CGGCGTATTCTTCTCGGACT-3′, and 6FAM-5′-GCCTAGGAAGCGCTCCGGCG-3′. These primers are specific for the B2L gene, which encodes the major membrane protein of parapoxvirus.

For virus culture, we crushed a biopsy sample and resuspended it in Hanks’ balanced salt solution (Thermo Fisher Scientific, https://www.thermofisher.com). We then inoculated 400 μL of this sample onto 2 shell vials (200 μL/vial) (7-mL TRAC bottles; Thermo Fisher Scientific) containing 1 mL of OA3.Ts testes cells from *Ovis aries* sheep (CRL-6546; American Type Culture Collection, https://www.atcc.org) at a concentration of 10^6^ cells/ mL. We incubated 1 vial at 32°C and 1 vial at 37°C in an atmosphere of 5% CO_2_ and observed daily by inverted microscopy to detect any potential cytopathic effect.

For virus production, we prepared 15 flasks (T75cm^2^; Corning, https://www.corning.com) containing OA3.Ts cells and Dulbecco’s modified Eagle medium (Thermo Fisher Scientific) plus 10% fetal bovine serum and 1% glutamine. We then incubated the cells, and when they reached a confluence of 80% of confluence, we removed the medium and inoculated the monolayer with 5 mL of virus suspension at a multiplicity of infection of 0.01. We incubated the flasks at 37°C for 1 h to enable adsorption. We then added 20 mL of Dulbecco’s modified Eagle medium to the flasks and incubated them for 3 days. On the third day, we discarded the supernatant, washed the cell monolayer 3 times with phosphate-buffered saline, and removed the monolayer by using a scraper. Once all the flasks were scraped and washed twice to collect cells, we transferred all contents to a 50-mL tube and kept the tube on ice.

We then centrifuged the cells at 500 × *g* for 10 min, removed the supernatant, resuspended the pellet in 10 mL of sterile lysis buffer (1 mmol/L MgCl_2_, 10 mmol/L Tris, and 10 mmol/L KCl, pH 7.0), and incubated this suspension for 10 min on ice. We performed mechanical lysis by using a sterile douncer device (80 cycles on ice). In parallel, we filtered the entire supernatant by using a 0.45-μm polyvinylidene difluoride membrane (Dutscher, https://www.dutscher.com) and centrifuged the supernatant. Finally, we added 10 mL of 25% sucrose to a plastic centrifugation tube, and slowly transferred the virus mixture from the filtrate to avoid mixing with the sucrose solution (biphasic final solution). We centrifuged the tube at 60,000 × *g* for 1 h at 4°C, collected the pellet, and stored the pellet at −80°C in small aliquots before genome sequencing.

### Sample Embedding and Cell Preparation

We maintained OA3.Ts cells in culture containing minimal essential medium plus 10% fetal bovine serum. We inoculated the cell monolayer with parapoxvirus at a multiplicity of infection of 0.01 and incubated. We then collected the contents after scraping the flask (T-25cm^2^) at 24 h postinfection.

We used the protocol of cell embedding as described in Bou Khalil et al. (*16*). We replaced the Epon resin with LR White resin (Agar Scientific, http://www.agarscientific.com). In brief, we fixed cells for 1 h with 2.5% glutaraldehyde in a 0.1 mol/L sodium cacodylate buffer and washed with a mixture of 0.2 mol/L saccharose/0.1 mol/L sodium cacodylate. We then postfixed cells for 1 h with 1% OsO4 diluted in 0.2 mol/L potassium hexacyanoferrate (III)/0.1 mol/L sodium cacodylate solution. After washing the cells with distilled water, we gradually dehydrated them with ethanol, then gradually replaced the ethanol with LR white resin. We performed polymerization for 24 h at 60°C. We obtained ultrathin, 70-nm sections by using a UC7 ultramicrotome (Leica, https://www.leica-microsystems.com) and placed the sections onto HR25 300-mesh copper/rhodium grids (TAAB, https://www.taab.co.uk). We colored the sections with Reynolds solution. We obtained electron micrographs by using a Tecnai G2 transmission electron microscope (FEI, https://www.fei.com) operated at 200 keV and used ImageJ software (https://imagej.nih.gov) to determine particle size.

### Genome Sequencing and Assembling

We sequenced genomic DNA of the parapoxvirus by using MiSeq Technology (Illumina, https://www.illumina.com) and the paired-end strategy. We barcoded sequences and compared them with 19 other genomic projects prepared from Nextera XT DNA Sample Prep Kit (Illumina). We quantified genomic DNA by using the Qubit Assay and the High-Sensitivity Kit (Life Technologies, https://www.thermofisher.com) at a concentration of 43 ng/µL. To prepare the paired-end library, we performed a dilution to obtain 1 ng of each genome as input to prepare the paired-end library. The tagmentation step (Illumina) fragmented and tagged the DNA. Limited cycle PCR amplification (12 cycles) completed the tag adapters and introduced dual-index barcodes. After purification by using AMPure XP Beads (Beckman Coulter Inc., https://www.beckmancoulter.com), we normalized the libraries on specific beads according to the Nextera XT protocol (Illumina). We pooled normalized libraries into a single library for sequencing, then loaded the pooled single-strand library onto the reagent cartridge and then onto the instrument along with the flow cell. We performed automated cluster generation and paired-end sequencing with dual index reads in a single 39-h run for 2 × 250 bp.

We obtained total information of 10.2 Gb from 1,140,000 clusters of density/mm^2^ and established a cluster passing quality control filters at 91.2% (19,783,000 clusters). Within this run, we determined the index representation for the parapoxvirus to be 5.45%. We filtered the 1,078,648 paired-end reads according to the read qualities.

We assembled paired-end reads by using the Hybrid spades program (*17*) and only paired-end strategy in input. We obtained 1 contig of 132,823 bp with an average coverage of 190 reads/base.

### Gene Prediction and Analysis

We used Prodigal software for gene prediction (*18*). For predicted proteins that had lengths <100 amino acids, we used Phyre 2 software to predict the tridimensional fold (*19*). For the 130 initial predicted proteins, we deleted 4 predicted proteins with abnormal folds. We performed a blastp analysis (https://blast.ncbi.nlm.nih.gov/Blast.cgi?PAGE=Proteins&) of all predicted proteins against the nonredundant database at the National Center for Biotechnology Information (https://www.ncbi.nlm.nih.gov) and performed annotation by using delta-blastp results (*20*) and Interproscan version 69.0 (https://www.ebi.ac.uk). To determine average nucleotide values, we compared close phylogenetic strains by using the OrthoANI algorithm (*21*); we compared predicted proteins by using the reciprocal best hit and ProteinOrtho software (*22*) with 80% coverage, 20% identity, and 10^−2^ as an E value cutoff. The genome is available in the EMBL-EBI database (https://www.ebi.ac.uk, accession no. LR594616).

### Phylogenetic Analysis

We performed alignment of 22 complete genomes of parapoxviruses with a closely related squirrel poxvirus, by using molluscum contagiosum virus as an outgroup. We computed alignments by using MAFFT version 7 (*23*) with fast Fourier transformation, a heuristic progressive method, and manually controlled alignments in MEGA6.0 (https://www.megasoftware.net) to delete inverted repeat regions nonaligned at both ends. We conserved 208,216 positions to build a tree by using the general time-reversible model on the PhyML version 3 program (*24*) and visualized trees by using the iTol online program (*25*).

## Results

### Virus Isolation and Ultrastructure

**Figure 3:**
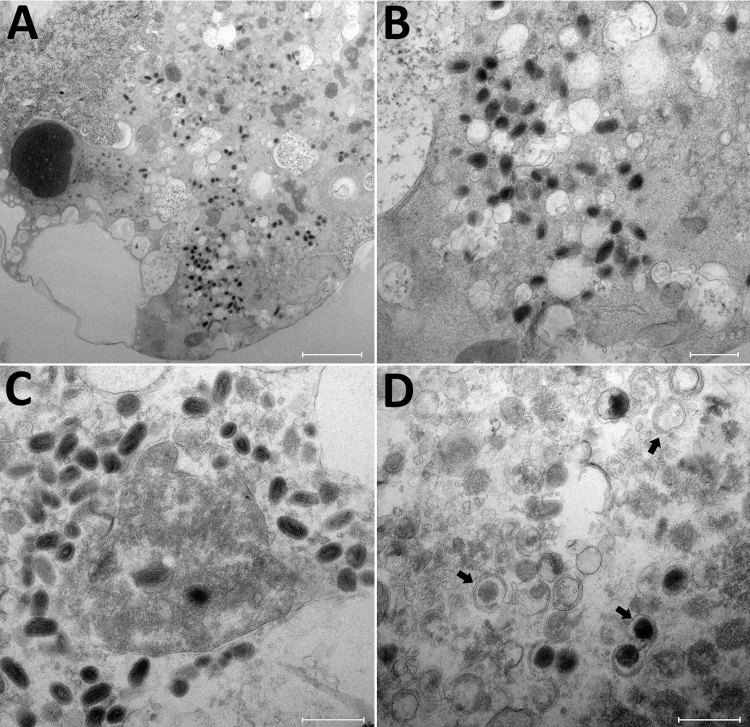
Transmission electron microscopy of OAT3.T cells infected with orf virus IHUMI-1 from a 65-year-old woman in France. A) Ultrathin section of an OAT3.Ts cell at 24 h postinfection harboring orf virus strain IHUMI-1 undergoing its replicative cycle where dense inclusion bodies could be clearly seen in the cell cytoplasm. B, C) Higher magnifications of infected cells showing typical enveloped virions. D) Ultrathin sections of an OAT3.Ts cell showing enveloped particles (arrows). Scale bars indicate 2 μm in panel A, 50 nm in panels B, C, and D.

The cutaneous biopsy specimen from the patient was found to contain parapoxvirus, which was confirmed by using a quantitative PCR. We inoculated this biopsy specimen onto OA3.Ts cells, and monitored lysis daily by using an inverted microscope. We detected a cytopathic effect at 48 h postinfection. Electron microscopy confirmed the presence of virions in cells at 24 h after reculture and by observations of ultrathin sections (Figure 3).

### Characterization of Orf Virus Genome

We obtained a linear complete genome of 132,823 bp with a guanine-cytosine–rich content of ≈64.4%. This genome is the third smallest by length among parapoxviruses, after orf virus strain NP and seal parapoxvirus (Table). We propose to name this orf virus strain IHUMI-1.

**Table:** Genomic characteristics of parapoxviruses used for analysis of an orf virus isolated from a 65-year-old woman infected during Aid-el-Fïtr, festival, France, 2017

Virus	Genome length, bp	GenBank accession no.	Source of virus	Reference
Orf virus strain PACA France 2017	132,823	LR594616	Hand nodule from human: 2017, France	This study
Orf virus strain OV-IA82	137,241	AY386263.1	Nasal secretion from lamb: 1982, Iowa	(*15*)
Orf virus strain NZ2	137,820	DQ184476.1	Sheep: New Zealand	(*26*)
Orf virus strain B029	134,104	KF837136.1	Human: Germany 1996	(*14*)
Orf virus strain OV-HN3/12	136,643	KY053526.1	Sheep: China 2012	(*27*)
Orf virus strain NA1/11	137,080	KF234407.1	Sheep: China 2011	(*27*)
Orf virus strain GO	139,866	KP010354.1	Lamb: Fujian, China, 2012	(*27*)
Orf virus strain D1701	134,038	HM133903.1	Sheep: Germany	(*28*)
Orf virus strain SJ1	139,112	KP010356.1	Lamb: Fujian, China, 2012	(*27*)
Orf virus strain YX	138,231	KP010353.1	Lamb: Fujian, China, 2012	(*27*)
Orf virus strain OV-SA00	139,962	AY386264.1	Goat kid: 2010, Texas	(*15*)
Orf virus strain NP	132,111	KP010355.1	Lamb: Fujian, China, 2011	(*27*)
Pseudocowpox virus strain VR634	145,289	GQ329670.1	Human after contact with contaminated cow: 1963, USA	(*29*)
Pseudocowpox virus strain F00.120R	133,169	GQ329669.1	Reindeer: Finland, 2009	(*29*)
Bovine papular stomatitis virus strain BV-TX09c1	135,072	KM875472.1	Domestic cow: 2009, USA	(*30*)
Bovine papular stomatitis virus strain BV-TX09c15	136,055	KM875470.1	Domestic cow: 2009, USA	(*30*)
Bovine papular stomatitis virus strain BV-TX09c5	135,635	KM875471.1	Domestic cow: 2009, USA	(*30*)
Bovine papular stomatitis virus strain BV-AR02	134,431	AY386265.1	Calf (oral lesions): Arkansas, 2004?	(*15*)
Parapoxvirus red deer/HL953	139,981	KM502564.1	Red deer (tonsil swab): Germany, 2013, subclinical infection	(*31*)
Seal parapoxvirus isolate AFK76s1	127,941	KY382358.2	Gray seal: Poland, 2015	(*1*)
Squirrel poxvirus strain red squirrel UK	148,803	HE601899.1	Red squirrel, UK: 2014, outgroup of parapoxvirus	(*32*)

Genome organization of orthopoxviruses are known to be conserved and follow the typical structure with inverted terminal repeat variations and a conserved central core genome (*33*,*34*). This structure was also suggested for parapoxviruses (*35*). We investigated synteny by using current complete genome reports. Mauve analysis of 12 complete genomes of orf virus enabled us to observe intraspecies conservation, except at the ends of some genomes (Appendix Figure 1). Moreover, analysis of synteny blocks across parapoxviruses showed the same typical organization (Appendix Figure 2). By using blast and MEGA analysis of the complete genome of orf virus IHUMI-1, we observed matches with all orf virus genomes, a squirrelpox virus genome, and a molluscum contagiosum virus genome (15% coverage and 80% identity). This region between the orf genome and molluscum contagiosum virus genome of ≈3,500 nt (positions on orf virus IHUMI-1 from 97,602 to 101,032) encodes a predicted protein essential for viruses: DNA-directed RNA polymerase subunit RPO132 (*Rbp2*).

Focusing on the 126 predicted proteins of virus strain IHUMI-1, we observed 124 best hits with different strains of orf virus, 1 hit with a hypothetical protein with *Mucor circinelloides* (with an E value of 10^−3^), and 1 hit with *Ovis aries* sheep, the natural host. The protein showing the best hit with *O. aries* sheep was annotated as the interleukin-10 precursor. The gene for this protein is found in parapoxvirus and was probably acquired from mammals and known as a potential keystone protein that reduces inflammation during the infectious cycle (*36*–*39*). Despite the position of this gene at the left start region of genomes of parapoxviruses, this protein is highly conserved. The interleukin-10 gene of orf virus IHUMI-1 shows 99% nucleotide sequence identity with other orf virus strains and 79% with *O. aries* sheep and with *Capra hircus* goats; the gene showed, as reported, numerous synonymous mutations and adaptations by orf virus (*39*).

### Virus Clusters and Distribution

**Figure 4:**
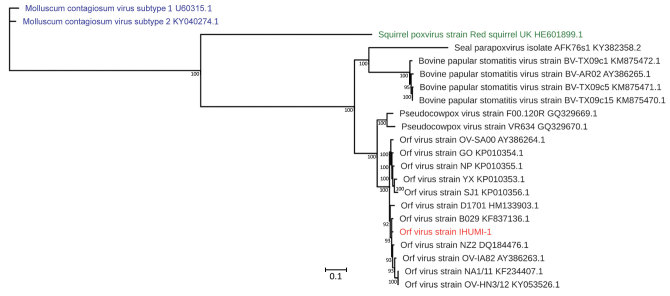
Maximum-likelihood tree based on complete sequences of orf virus IHUMI-1 from a 65-year-old woman in France (red) and 22 other viruses belonging to the family *Poxviridae*. Tree was constructed by using a general time-reversible model with 100 bootstrap replicates. All branches with bootstrap values <70 were collapsed. Numbers along branches are bootstrap values. Blue indicates 2 chordopoxviruses that served as outgroups, and green indicates a squirrel poxvirus still unclassified but related to the genus *Parapoxvirus*. GenBank accession numbers are provided for reference isolates. Scale bar indicates nucleotide substitutions per site.

**Figure 5:**
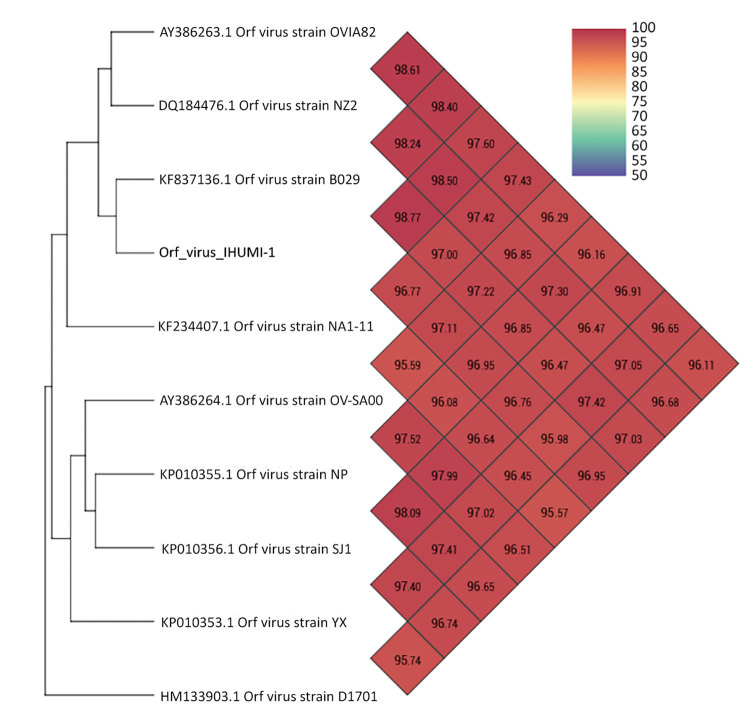
Heatmap representation of strain proximities across complete genomes of orf virus IHUMI-1 from a 65-year-old woman in France (red) and other available orf viruses. Because of the OrthoANi algorithm constraint, we deleted the complete genome of orf virus strain GO (GenBank accession no. KP010354.1) that clusters with orf virus strain NP and the complete genome of orf virus strain OV-HN3/12 (that clusters with NA1-11). GenBank accession numbers are provided for reference isolates. Values indicate percent similarity of nucleotides.

Orthopoxviruses, such as various strains of cowpox virus, circulate in Europe, and clusters are well identified with clades and subclades (*40*,*41*). Concerning parapoxviruses, a previous study of whole genomes of orf viruses showed that clusters exist and depend on whether the host is a goat or a sheep (*27*). Our phylogenetic analysis performed on whole genomes of parapoxviruses, which used the maximum-likelihood method, identified clusters with 2 different branches of orf viruses that had a common ancestor. This result is similar to that of Chi et al. (*27*) and showed 2 branches depending on whether the virus host was a goat or a sheep (Figure 4). In contrast, analysis by using the OrthoANi algorithm enabled us to separate orf viruses that originated from sheep and those that originated from goats (Figure 5). The only difference we observed was for orf virus strain D1701, which appeared to be an outgroup strain. Nevertheless, when we used the maximum-likelihood method and the OrthoANi algorithm, we found that orf virus IHUMI-1 clustered with orf virus strain B029. These 2 strains were human isolates obtained after infection from sheep.

Reciprocal best hit analysis enabled us to observe a high degree of conservation across orf virus genomes. A total of 102 proteins composed the core genomes of 12 orf viruses, and we did not detect any differences in core genomes of orf virus clusters. All virus proteins known to be essential (e.g., vascular endothelial growth factor, interleukin-10, and nuclear factor-κB inhibitor protein) (*39*,*42*,*43*) are present in the genome of orf virus strain IHUMI-1.

Protein analysis showed an absence of a predicted homolog open reading frame 119 in orf virus IHUMI-1. For his region, Chi et al. (*27*) reported numerous deletions and gap sequences (Appendix Figure 3), especially for 3 strains (NP, SJ1, and IHUMI-1). Coverage was <80% in that region for strains SJ1 and IHUMI-1, and the gene was almost completely deleted for strain NP (only 24% coverage) compared with strain OV-SA00. The consequence for orf virus IHUMI-1 is the deletion of the ORF119 gene. This deletion has been implied in cell apoptosis (*44*,*45*). Nevertheless, deletion of this gene did not affect the virus cycle and strain virulence (*46*).

## Discussion

We determined the complete genome of orf virus strain IHUMI-1 isolated from a human. This virus is the third smallest (by genome length) in the genus *Parapoxvirus*. The genome organization of orf virus IHUMI-1 is extremely similar in its synteny with those of other orf viruses that do not have a genetic inversion. Analysis of the predicted protein highlights strong protein conservation, except for the deletion in the ORF119 gene. The mitochondrial protein coded by this gene was recently described as being capable of increasing cell apoptosis (*44*,*45*). Despite this finding, our observations and a previous report showed no phenotypic modification in the virus cycle regarding this gene deletion (*46*).

Conversely, phylogenetic analysis of the entire virus genome showed clustering of orf viruses depending on the host (sheep or goats). This result was similar to those of previous analyses performed on different complete genomes by Chi et al. (*27*). Some studies did not report similar results; however, the phylogenetic trees in those studies were limited to analysis of a few genes, such as the partial B2L gene (*47*–*49*). More complete genomes are needed to confirm this trend and verify there are 2 types of orf virus. In addition, we observed clustering on the whole genome between the IHUMI-1 and B029 strains of orf virus after human infection. Further investigations using more complete genome sequences might be able to confirm if some genetically related strains have the potential capacity to cross species barriers.

Numerous strains of parapoxviruses that infect animals are believed to show variable virulence in humans (e.g., orf strain D1701). However, implication of the host immune system in the severity of orf virus disease and in its evolution have been demonstrated (*50*).

Our results highlight the necessity of obtaining more complete genomes for parapoxviruses and retracing the route of infection when humans are infected. Further investigations of parapoxviruses should address the difficulties in isolating and cultivating this fastidious virus by using nonconventional cells for diagnostic analysis. However, recent description of a seal parapoxvirus (*1*) with a high-quality genome sequence obtained directly from a clinical sample could bypass the culture problem. In contrast, isolating the viral particle will always help to improve clinical research and future innovations.

Appendix Additional information on human infection with orf virus and description of its whole genome, France, 2017.
