# Supplementary material for: Human Infection with Orf Virus and Description of Its Whole Genome, France, 2017
Source: Emerg Infect Dis. 2019 Dec;25(12):2197–204. doi: 10.3201/eid2512.181513 (PMC6874271; doi:10.3201/eid2512.181513)
Supplement: Appendix — Additional information on human infection with orf virus and description of its whole genome, France, 2017. [file 18-1513-Techapp-s1.pdf]

Article DOI: https://doi.org/10.3201/eid2512.181513

Human Infection with Orf Virus and Description of Its Whole Genome, France, 2017

Appendix

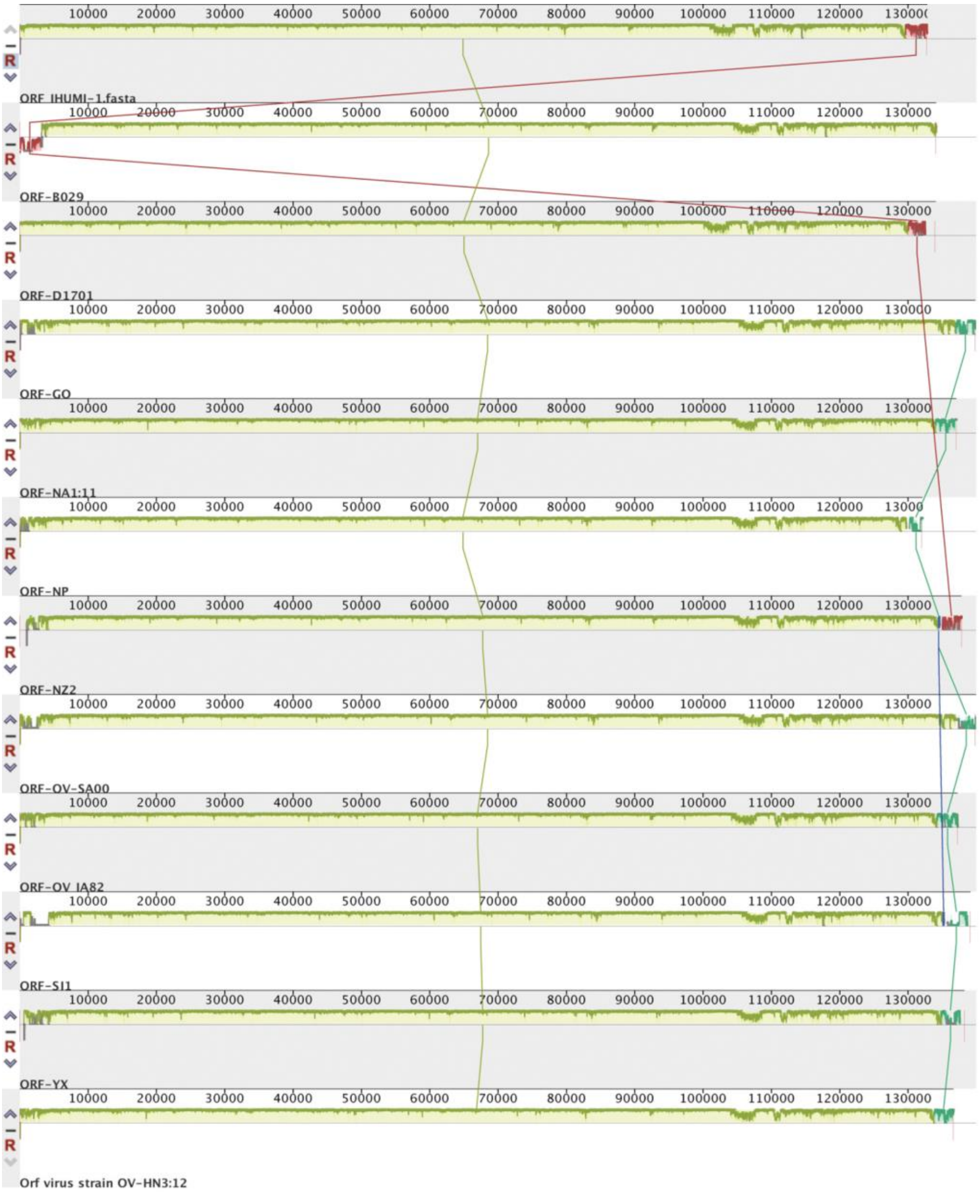

Appendix Figure 1. Mauve alignment obtained for orf virus genomes, France, 2017.

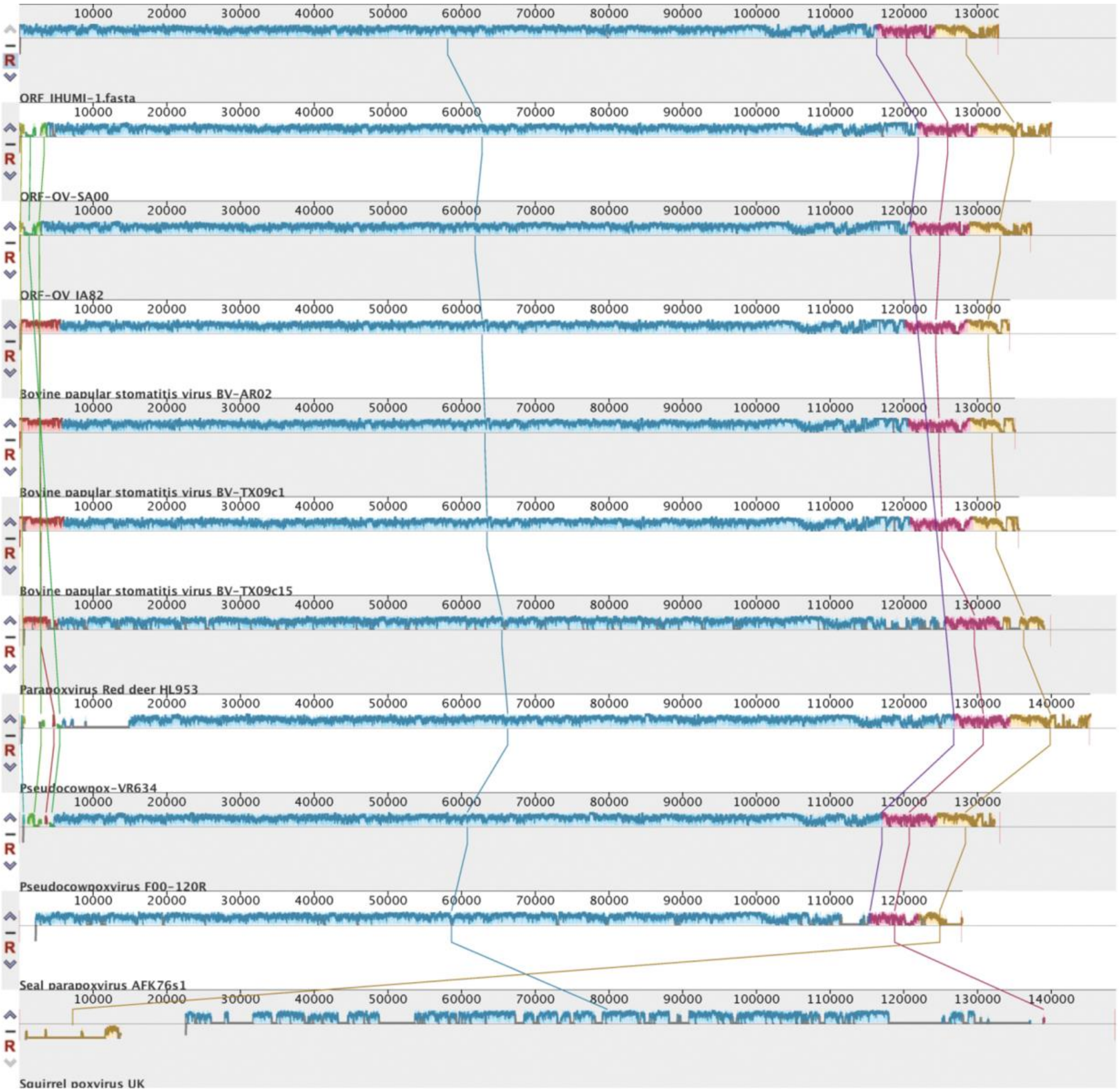

Appendix Figure 2. Mauve alignment obtained for different parapoxvirus genomes, France, 2017.

Appendix Figure 3 (below). Alignment of sequences for orf viruses, France, 2017.

Tools (/Tools/) > Multiple Sequence Alignment (/Tools/msa) > MView

Results for job mview-I20180712-133951-0236-40078163-p1m

```
Reference sequence (1): Orf_virus_OV-SA00_NC_005336.1_115015-122053
Identities normalised by aligned length.
Colored by: identity
```

```
                                                    cov    pid   1 [         .         .         .         .         :         .         .         . 80
 1 Orf_virus_OV-SA00_NC_005336.1_115015-122053 100.0% 100.0%     TACGACGAGTAGCCGCCAAAACTGAATAACTATCGGACTTCGTAAACTCGCAGACATGCCGCTGTTCCGGAAGCTCATGG
 2 Orf_virus_D1701                               98.3%  88.3%     TACGACGAGTAGCCGCCAAAACTGAATAACTATCGGGCTTCGTAAACGCGCAGACATGCCGCTGTTCCGGAAGCTCATGG
 3 Orf_virus_NA1-11                              97.9%  87.5%     TACGACGAGTAGCCGCCAAAACTGAATAACTATCGGGCTTCGTAAACGCGCAGACATGCCGCTGTTCCGGAAGCTCATGG
 4 Orf_virus_OV-HN3_12                           98.1%  87.2%     TACGACGAGTAGCCGCCAAAACTGAATAACTATCGGGCTTCGTAAACGCGCAGACATGCCGCTGTTCCGGAAGCTCATGG
 5 Orf_virus_OV-IA82                             98.6%  88.5%     TACGACGAGTAGCCGCCAAAACTGAATAACTATCGGGCTTCGTAAACGCGCAGACATGCCGCTGTTCCGGAAGCTCATGG
 6 Orf_virus_NZ2                                 98.5%  88.2%     TACGACGAGTAGCCGCCAAAACTGAATAACTATCGGGCTTCGTAAACGCGCAGACATGCCGCTGTTCCGGAAGCTCATGG
 7 Orf_virus_YX                                  98.1%  95.5%     TACGACGAGTAGCCGCCAAAACTGAATAACTATCGGACTTCGTAAACTCGCAGACATGCCGCTGTTCCGGAAGCTCATGG
 8 Orf_virus_GO                                  98.8%  96.4%     TACGACGAGTAGCCGCCAAAACTGAATAACTATCGGACTTCGTAAACTTGCAGACATGCCGCTGTTCCGGAAGCTCATGG
 9 Orf_virus_B029                                91.8%  81.7%     TACGACGAGTAGCCGCCAAAACTGAATAACTACCGGACTTCGTAAACGCGCAGACATGCCGCTGTTCCGGAAGCTCATGG
10 Orf_virus_IHUMI-1                             78.5%  70.9%     TACGACGAGTAGCCGCCAAAACTGAATAACTATCGGACTTCGTAAACTCGCAGACATGCCGCTGTTCCGGAAGCTCATGG
11 Orf_virus_SJ1                                 77.2%  74.2%     TACGACGAGTAGCCGCCAAAACTGAATAACTATCGGACTTCGTAAACTCGCAGACATGCCGCTGTTCCGGAAGCTCATGG
12 Orf_virus_NP                                  24.1%  23.5%     TACGACGAGTAGCCGCCAAAACTGAATAACTATCGGACTTCGTAAACTCGCAGACATGCCGCTGTTCCGGAAGCTCATGG
   consensus/100%                                                TACGACGAGTAGCCGCCAAAACTGAATAACTAsCGGuCTTCGTAAACssGCAGACATGCCGCTGTTCCGGAAGCTCATGG
   consensus/90%                                                 TACGACGAGTAGCCGCCAAAACTGAATAACTATCGGuCTTCGTAAACsCGCAGACATGCCGCTGTTCCGGAAGCTCATGG
   consensus/80%                                                 TACGACGAGTAGCCGCCAAAACTGAATAACTATCGGuCTTCGTAAACsCGCAGACATGCCGCTGTTCCGGAAGCTCATGG
   consensus/70%                                                 TACGACGAGTAGCCGCCAAAACTGAATAACTATCGGuCTTCGTAAACsCGCAGACATGCCGCTGTTCCGGAAGCTCATGG

                                                    cov    pid  81           .         1         .         .         .         .         :         . 160
 1 Orf_virus_OV-SA00_NC_005336.1_115015-122053 100.0% 100.0%     TCTCCCGCGCCCTCGTCAAGGAATGTTTGACTCTGGACTTCCGGCAGGGCGAGCGTCTCCCCACCCGATGCTTCCTCCCG
 2 Orf_virus_D1701                               98.3%  88.3%     TCTCTCGCGCCCTCGTCAAGGAATGTCTGACTCTGGACTTCCGGCAGGGCGAGCGTCTGCCCACGCGATGCTTCCTCCCG
 3 Orf_virus_NA1-11                              97.9%  87.5%     TTTCGCGCTCCCTGGTCAAGGAATGTCTGACTCTGGACTTCCGGCAGGGCGAGCGTCTCCCCACGCGATGCTTCCTCCCA
 4 Orf_virus_OV-HN3_12                           98.1%  87.2%     TTTCGCGCTCCCTGGTCAAGGAATGTCTGACTCTGGACTTCCGGCAGGGCGAGCGTCTCCCCACGCGATGCTTCCTCCCA
 5 Orf_virus_OV-IA82                             98.6%  88.5%     TTTCGCGCTCCCTGGTCAAGGAATGTCTGACTCTGGACTTCCGGCAGGGCGAGCGTCTCCCCACCCGATGCTTCCTCCCG
 6 Orf_virus_NZ2                                 98.5%  88.2%     TTTCGCGCTCCCTGGTCAAGGAATGTCTGACTCTGGACTTCCGGCAGGGCGAGCGTCTCCCTACGCGATGCTTCCTCCCG
 7 Orf_virus_YX                                  98.1%  95.5%     TCTCCCGCGCCCTCGTCAAGGAATGTCTGACTCTGGACTTCCGGCAGGGCGAGCGTCTCCCCACCCGATGCTTCCTCCCG
 8 Orf_virus_GO                                  98.8%  96.4%     TCTCCCGCGCCCTCGTCAAGGAATGTCTGACTCTGGACTTCCGGCAGGGCGAGCGTCTCCCCACCCGATGCTTCCTCCCG
 9 Orf_virus_B029                                91.8%  81.7%     TTTCGCGCTCCCTGGTCAAGGAATGTCTGACTCTGGACTTCCGGCAGGGCGAGCGTCTCCCCACGCGATGCTTCCTCCCG
10 Orf_virus_IHUMI-1                             78.5%  70.9%     TTTCGCGCTCCCTGGTCAAAGAATGTCTGACTCTGGACTTCCGGCAGGGCGAGCGTCTGCCCACGCGATGCTTCCTCCCG
11 Orf_virus_SJ1                                 77.2%  74.2%     TCTCCCGCGCCCTCGTTAAGGAATGTCTGACTCTGGACTTCCGGCAGGGCGAGCGTCTCCCCACCCGATGCTTCCTCCCG
12 Orf_virus_NP                                  24.1%  23.5%     TCTCCCGCGCCCTCGTCAAGGAATGTCTGACTCTGGACTTCCGGCAGGGCGAGCGTCTCCCCACCCGATGCTTCCTCCCG
   consensus/100%                                                TsTCsCGCsCCCTsGTsAAuGAATGTsTGACTCTGGACTTCCGGCAGGGCGAGCGTCTsCCsACsCGATGCTTCCTCCCu
   consensus/90%                                                 TsTCsCGCsCCCTsGTCAAGGAATGTCTGACTCTGGACTTCCGGCAGGGCGAGCGTCTsCCCACsCGATGCTTCCTCCCu
   consensus/80%                                                 TsTCsCGCsCCCTsGTCAAGGAATGTCTGACTCTGGACTTCCGGCAGGGCGAGCGTCTCCCCACsCGATGCTTCCTCCCG
   consensus/70%                                                 TsTCsCGCsCCCTsGTCAAGGAATGTCTGACTCTGGACTTCCGGCAGGGCGAGCGTCTCCCCACsCGATGCTTCCTCCCG

                                                    cov    pid 161           .         .         .         2         .         .         .         . 240
 1 Orf_virus_OV-SA00_NC_005336.1_115015-122053 100.0% 100.0%     GTGCCCGCGGGGACGACATTCCACAGAGTCTGCGACACCTCGCCGCTGACGAACGAAGTCTCCAGGCACGTGCAGGAGCC
 2 Orf_virus_D1701                               98.3%  88.3%     GTGCCCGCGGGGACGACATTCCACAGAGTCTGCGACACCTCGCCGCTGACAAACGAAGTCTCCCGGCACGTGCAGGAGCC
 3 Orf_virus_NA1-11                              97.9%  87.5%     GTGCCCGCTGGGACGACATTCCACAGAGTCTGCGACACTTCGCCGCTGACGGACGAAGTCTCCCGGCACGTGCAGGAGCC
 4 Orf_virus_OV-HN3_12                           98.1%  87.2%     GTGCCCGCTGGGACGACATTCCACAGAGTCTGCGACACTTCGCCGCTGACGGACGAAGTCTCCCGGCACGTGCAGGAGCC
 5 Orf_virus_OV-IA82                             98.6%  88.5%     GTGCCCGCGGGGACGACATTCCACAGAGTCTGCGACACCTCGCCGCTGACGGACGAAGTATCCCGGCACGTGCAGGAGCC
 6 Orf_virus_NZ2                                 98.5%  88.2%     GTGCCCGCGGGGACGACATTCCACAGAGTCTGCGACACCTCGCCGCTGACGGACGAAGTCTCCCGGCACGTGCAGGAGCC
 7 Orf_virus_YX                                  98.1%  95.5%     GTGCCCGCGGGGACGACATTCCACAGAGTCTGCGACACCTCGCCGCTGACGAACGAAGTCTCCAGGCACGTGCAGGAGCC
 8 Orf_virus_GO                                  98.8%  96.4%     GTGCCCGCGGGGACGACATTCCACAGAGTCTGCGACACCTCGCCGCTGACGAACGAAGTCTCCCGGCACGTGCAGGAGCC
 9 Orf_virus_B029                                91.8%  81.7%     GTGCCCGCGGGGACGACATTCCACAGAGTCTGCGACACCTCGCCGCTGACGGACGAAGTCTCCCGGCACGTGCAGGAGCC
10 Orf_virus_IHUMI-1                             78.5%  70.9%     GTGCCCGCAGGGACAACCTTCCACAGAGTCTGCGACACCTCGCCGCTGACGGACGAAGTCTCCCGGCACGTGCAGGAGCC
11 Orf_virus_SJ1                                 77.2%  74.2%     GTGCCCGCGGGGACGACATTCCACAGAGTCTGCGACACCTCGCCGCTGACGAACGAAGTCTCCCGGCACGTGCAGGAGCC
12 Orf_virus_NP                                  24.1%  23.5%     GTGCCCGCGGGGACGACATTCCACAGAGTCTGCGACACCTCGCCGCTGACAAACGAAGTCTCCCGGCACGTGCAGGAGCC
   consensus/100%                                                GTGCCCGCsGGGACuACsTTCCACAGAGTCTGCGACACsTCGCCGCTGACuuACGAAGTsTCCsGGCACGTGCAGGAGCC
   consensus/90%                                                 GTGCCCGCsGGGACGACATTCCACAGAGTCTGCGACACsTCGCCGCTGACuuACGAAGTCTCCsGGCACGTGCAGGAGCC
   consensus/80%                                                 GTGCCCGCuGGGACGACATTCCACAGAGTCTGCGACACCTCGCCGCTGACGuACGAAGTCTCCCGGCACGTGCAGGAGCC
   consensus/70%                                                 GTGCCCGCGGGGACGACATTCCACAGAGTCTGCGACACCTCGCCGCTGACGuACGAAGTCTCCCGGCACGTGCAGGAGCC

                                                    cov    pid 241           :         .         .         .         .         3         .         . 320
 1 Orf_virus_OV-SA00_NC_005336.1_115015-122053 100.0% 100.0%     CGTCATGGGCACCGGACGGGTCCAGTACTACTACTTCGAGAGCGGCCAGGGCATGATCGGCGACAACGCGGGCACGCCGC
 2 Orf_virus_D1701                               98.3%  88.3%     CGTCATGGGCACCGGACGGGTCCAGTACTACTACTTCGAGAGCGGCCAGGGCATGATCGGCGACAACGCGGGCATGCCGC
 3 Orf_virus_NA1-11                              97.9%  87.5%     CGTCATGGGCACCGGACGAGTCCAGTACTACTACTTCGAGAGCGGCCAGGGCATGATCGGCGACAACGCGGGCATGCCGC
 4 Orf_virus_OV-HN3_12                           98.1%  87.2%     CGTCATGGGCACCGGACGAGTCCAGTACTACTACTTCGAGAGCGGCCAGGGCATGATCGGCGACAACGCGGGCATGCCGC
 5 Orf_virus_OV-IA82                             98.6%  88.5%     CGTCATGGGCACCGGACGGGTCCAGTACTACTACTTCGAGAGCGGGCAGGGCATGATCGGCGACAACGCGGGCATGTCGC
 6 Orf_virus_NZ2                                 98.5%  88.2%     CGTCATGGGCACCGGACGGGTCCAGTACTACTACTTCGAGAGCGGCCAGGGCATGATCGGCGACAACGCGGGCATGGCGC
 7 Orf_virus_YX                                  98.1%  95.5%     CGTCATGGGCACCGGACGGGTCCAGTACTACTACTTCGAGAGCGGCCAGGGCATGATCGGCGACAACGCGGGCACGCCGC
 8 Orf_virus_GO                                  98.8%  96.4%     CGTCATGAGCACCGGAAGGGTCCAGTACTACTACTTCGAGAGCGGCCAGGGCATGATCGGCGACAACGCGGGAATGCCGC
 9 Orf_virus_B029                                91.8%  81.7%     CGTCATGGGCACCGGACGGGTCCAGTACTACTACTTCGAGAGCGGGCAGGGCATGATCGGCGACAACGCGGGCATGGCGC
10 Orf_virus_IHUMI-1                             78.5%  70.9%     CGTCATGGGCACCGGACGGGTCCAGTACTACTACTTCGAGAGCGGCCAGGGCATGATCGGCGACAACGCGGGCATGCCGC
11 Orf_virus_SJ1                                 77.2%  74.2%     CGTCATGGGCACCGGACGGGTCCAGTACTACTACTTCGAGAGCGGCCAGGGCATGATCGGCGACAACGCGGGCACGCCGC
12 Orf_virus_NP                                  24.1%  23.5%     CGTCGCATGCACCGGACGGGTCCAGTACTACTACTTCGAGAGCGGCCAGGGCATGATCGGCGACAACGCGGGCA------
   consensus/100%                                                CGTCususGCACCGGAsGuGTCCAGTACTACTACTTCGAGAGCGGsCAGGGCATGATCGGCGACAACGCGGGsA......
   consensus/90%                                                 CGTCATGuGCACCGGACGuGTCCAGTACTACTACTTCGAGAGCGGsCAGGGCATGATCGGCGACAACGCGGGCAsGsCGC
   consensus/80%                                                 CGTCATGGGCACCGGACGGGTCCAGTACTACTACTTCGAGAGCGGCCAGGGCATGATCGGCGACAACGCGGGCAsGsCGC
   consensus/70%                                                 CGTCATGGGCACCGGACGGGTCCAGTACTACTACTTCGAGAGCGGCCAGGGCATGATCGGCGACAACGCGGGCAsGsCGC

                                                    cov    pid 321           .         .         :         .         .         .         .         4 400
```

```
 1 Orf_virus_OV-SA00_NC_005336.1_115015-122053 100.0% 100.0%      GCATGCTCGTGTGCACGCGGTCGGCGTACAACGGCGGCGACGTCGTCGTGCGGTCCACGCGGAGCAGAGCAGACAAAACC
 2 Orf_virus_D1701                              98.3%  88.3%      G---GCTCGTGTGCACGCGCTCGGCGTACAACGGCGGCGACGTCGTCGTGCGGTCCACGCGGAGCAGAGCGGACAAGACC
 3 Orf_virus_NA1-11                             97.9%  87.5%      GCATGCTCGTGTGCACGCGCTCGGCGTACAACGGCGGCGACGTCGTCGTGCGGTCCACGCGGAGCAGAGCAGACAAGACC
 4 Orf_virus_OV-HN3_12                          98.1%  87.2%      GCATGCTCGTGTGCACGCGCTCGGCGTACAACGGCGGCGACGTCGTCGTGCGGTCCACGCGGAGCAGAGCAGACAAGACC
 5 Orf_virus_OV-IA82                            98.6%  88.5%      GCATGCTCGTGTGCACGCGCTCGGCGTACAACGGCGGCGACGTCGTCGTGCGGTCCACGCGGAGCAGAGCAGACAAGACC
 6 Orf_virus_NZ2                                98.5%  88.2%      GCATGCTCGTGTGCACGCGGTCGGCGTACAACGGCGGCGACGTCGTCGTGCGGTCCACGCGGAGCAGAGCAGACAAGACC
 7 Orf_virus_YX                                 98.1%  95.5%      GCATGCTCGTGTGCACGCGGTCGGCGTACAACGGCGGCGACGTCGTCGTGCGGTCCACGCGGAGCAGAGCAGACAAAACC
 8 Orf_virus_GO                                 98.8%  96.4%      GCATGCTCGTGTGCACGCGGTCGGCGTACAACGGCGGCGACGTCGTCGTGCGGTCCACGCGGAGCAGAGCAGACAAAACC
 9 Orf_virus_B029                               91.8%  81.7%      GCATGCTCGTGTGCACGCGGTCGGCGTACAACGGCGGCGACGTCGTCGTGCGGTCCACGCGGAGCAGAGCAGACAAGACT
10 Orf_virus_IHUMI-1                            78.5%  70.9%      GCATGCTCGTGTGCACGCGCTCGGCGTACAACGGCGGCGACGTCGTCGTGCGGTCCACGCGGAGCAGAGCAGACAAGACC
11 Orf_virus_SJ1                                77.2%  74.2%      GCATGCTCGTGTGCACGCGGTCGGCGTACAACGGCGGCGACGTCGTCGTGCGGTCCACGCGGAGCAGAGCAGACAAAACC
12 Orf_virus_NP                                 24.1%  23.5%      --------------------------------------------------------------------------------
   consensus/100%                                                 ................................................................................
   consensus/90%                                                  G...GCTCGTGTGCACGCGsTCGGCGTACAACGGCGGCGACGTCGTCGTGCGGTCCACGCGGAGCAGAGCuGACAAuACs
   consensus/80%                                                  GCATGCTCGTGTGCACGCGsTCGGCGTACAACGGCGGCGACGTCGTCGTGCGGTCCACGCGGAGCAGAGCAGACAAuACC
   consensus/70%                                                  GCATGCTCGTGTGCACGCGsTCGGCGTACAACGGCGGCGACGTCGTCGTGCGGTCCACGCGGAGCAGAGCAGACAAuACC

                                                  cov    pid 401         .         .         .         .         :         .         .         . 480
 1 Orf_virus_OV-SA00_NC_005336.1_115015-122053 100.0% 100.0%      GTGGTCGCGCCCTGCCAGGGCATGGTGCTGCTGCTGAGCCCCTTCTGCGCCTTCGACATCACGCCGGTGGAGAGCGGCTC
 2 Orf_virus_D1701                              98.3%  88.3%      GTGGTCGCGCCCTGCCAGGGCATGGTGCTGCTGCTGAGCCCCTTCTGCGCCTTCGACATCACGCCGGTGGAGAGCGGCTC
 3 Orf_virus_NA1-11                             97.9%  87.5%      GTGGTCGCGCCCTGCCAGGGCATGGTGCTGCTGCTGAGCCCCTTCTGCGCCTTCGACATCACGCCGGTGGAGAGCGGCTC
 4 Orf_virus_OV-HN3_12                          98.1%  87.2%      GTGGTCGCGCCCTGCCAGGGCATGGTGCTGCTGCTGAGCCCCTTCTGCGCCTTCGACATCACGCCGGTGGAGAGCGGCTC
 5 Orf_virus_OV-IA82                            98.6%  88.5%      GTGGTCGCGCCCTGCCAGGGCATGGCGCTGCTGCTGAGCCCCTTCTGCGCCTTCGACATCACGCCGGTGGAGAGCGGCTC
 6 Orf_virus_NZ2                                98.5%  88.2%      GTGGTCGCGCCCTGCCAGGGCATGGCGCTGCTGCTGAGCCCCTTCTGCGCCTTCGACATCACGCCGGTCGAGAGCGGCTC
 7 Orf_virus_YX                                 98.1%  95.5%      GTGGTCGCGCCCTGCCAGGGCATGGTGCTGTTGCTGAGTCCCTTCTGCGCCTTCGACATCACGCCGGTCGAGAGCGGCTC
 8 Orf_virus_GO                                 98.8%  96.4%      GTGGTCGCGCCCTGCCAGGGCATGGTGCTGTTGCTGAGTCCCTTCTGCGCCTTCGACATCACGCCGGTCGAGAGCGGCTC
 9 Orf_virus_B029                               91.8%  81.7%      GTGGTCGCGCCCTGCCAGGGCATGGCGCTGCTGCTGAGCCCCTTCTGCGCCTTCGACATCACGCCGGTGGAGAGCGGCTC
10 Orf_virus_IHUMI-1                            78.5%  70.9%      GTGGTCGCGCCCTGCCAGGGCATGGCGCTGCTGCTGAGCCCCTTCTGCGCCTTCGACATCACGCCGGTGGAGAGCGGCTC
11 Orf_virus_SJ1                                77.2%  74.2%      GTGGTCGCGCCCTGCCAGGGCATGGTGCTGCTGCTGAGCCCCTTCTGCGCCTTCGACATCACGCCGGTGGAGAGCGGCTC
12 Orf_virus_NP                                 24.1%  23.5%      --------------------------------------------------------------------------------
   consensus/100%                                                 ................................................................................
   consensus/90%                                                  GTGGTCGCGCCCTGCCAGGGCATGGsGCTGsTGCTGAGsCCCTTCTGCGCCTTCGACATCACGCCGGTsGAGAGCGGCTC
   consensus/80%                                                  GTGGTCGCGCCCTGCCAGGGCATGGsGCTGsTGCTGAGsCCCTTCTGCGCCTTCGACATCACGCCGGTsGAGAGCGGCTC
   consensus/70%                                                  GTGGTCGCGCCCTGCCAGGGCATGGsGCTGCTGCTGAGCCCCTTCTGCGCCTTCGACATCACGCCGGTsGAGAGCGGCTC

                                                  cov    pid 481         .         5         .         .         .         .         :         . 560
 1 Orf_virus_OV-SA00_NC_005336.1_115015-122053 100.0% 100.0%      CGCGATATTCGCGGAGGTCACGGTCGGCGCGCCCAGCATGGACCACGTCGAGGCGGTCGCCGGCGACGGCGACGCGGCCG
 2 Orf_virus_D1701                              98.3%  88.3%      CGCGATATTCGCGGAGGTCATCGTCACCGCGCCCAGCATGGACCACGTCGAGGCGGTCACCGGCACGGGCGAGGCGGCCG
 3 Orf_virus_NA1-11                             97.9%  87.5%      CGCGATATTTGCGGAGGTCATCGTCACCGCGCCCAGCATGGACCACGTCGAGGCGGTCACCGGCACGGGCGAGGCGGCCG
 4 Orf_virus_OV-HN3_12                          98.1%  87.2%      CGCGATATTTGCGGAGGTCATCGTCACCGCGCCCAGCATGGACCACGTCGAGGCGGTCACCGGCACGGGCGAGGCGGCCG
 5 Orf_virus_OV-IA82                            98.6%  88.5%      CGCGATATTCGCGGAGGTCATCGTCACTTCGCCCAGCATGGACCACGTCGAGGCGGTCACCGGCACGGGCGAGGCGGCCG
 6 Orf_virus_NZ2                                98.5%  88.2%      CGCGATATTCGCGGAGGTCATCGTCACCGCGCCCAGCATGGACTACGTCGAGGCGGTCACCGGCACGGGCGAGGCGGCCG
 7 Orf_virus_YX                                 98.1%  95.5%      CGCGATATTCGCGGAGGTCACGGTCGGCGCGCCCAGCATGGACCACGTCGAGGCGGTCGCCGGCGACGGCGACGCGGCCG
 8 Orf_virus_GO                                 98.8%  96.4%      CGCGATATTCGCGGAGGTCACGGTCGGCGCGCCCAGCATGGACCACGTCGAGGCGGTCGCCGGAGACGGCGACGCGGCCG
 9 Orf_virus_B029                               91.8%  81.7%      CGCGATATTCGCGGAGGTCATCGTCACCGCGCCCAGCATGGACCACGTCGAGGCGGTCACCGGCACGGGCGAGGCGGCCG
10 Orf_virus_IHUMI-1                            78.5%  70.9%      CGCGATATTCGCGGAGGTCATCGTCACCGCGCCCAGCATGGACCACGTCGAGGCGGTCACCAGCACGGGCGAGGCGGCCG
11 Orf_virus_SJ1                                77.2%  74.2%      CGCGATATTCGCGGAGGTCACGGTCGGCGCGCCCAGCATGGACCACGTCGAGGCGGTCGCCGGTGACGGCGACGCGGCCG
12 Orf_virus_NP                                 24.1%  23.5%      --------------------------------------------------------------------------------
   consensus/100%                                                 ................................................................................
   consensus/90%                                                  CGCGATATTsGCGGAGGTCAssGTCusssCGCCCAGCATGGACsACGTCGAGGCGGTCuCCuGsussGGCGAsGCGGCCG
   consensus/80%                                                  CGCGATATTsGCGGAGGTCAssGTCusCGCGCCCAGCATGGACCACGTCGAGGCGGTCuCCGGsussGGCGAsGCGGCCG
   consensus/70%                                                  CGCGATATTCGCGGAGGTCAssGTCusCGCGCCCAGCATGGACCACGTCGAGGCGGTCuCCGGCussGGCGAsGCGGCCG

                                                  cov    pid 561         .         .         .         6         .         .         .         . 640
 1 Orf_virus_OV-SA00_NC_005336.1_115015-122053 100.0% 100.0%      TGCGGATATTCAACTCGCACCACCCGCTCTGGCCGCGACACGGCTCAAACGTCTGCTTCGCGCTGCGGTTGCTGCGAGAC
 2 Orf_virus_D1701                              98.3%  88.3%      TGCGGATATTCAACTCGCACCACCCGCTCTGGCCGCGACACGGCTCGAACGTCTGCTTCGCGCTGCGGTTGCTGCGAGAC
 3 Orf_virus_NA1-11                             97.9%  87.5%      TGCGGATATTCAACTCGCACCACCCGCTCTGGCCGCGACACGGCTCGAACGTCTGCTTCGCGCTGCGGTTGCTGCGAGAC
 4 Orf_virus_OV-HN3_12                          98.1%  87.2%      TGCGGATATTCAACTCGCACCACCCGCTCTGGCCGCGACACGGCTCGAACGTCTGCTTCGCGCTGCGGTTGCTGCGAGAC
 5 Orf_virus_OV-IA82                            98.6%  88.5%      TGCGGATATTCAACTCGCACCACCCGCTCTGGCCGCGACACGGCTCGAACGTCTGCTTCGCGCTGCGGTTGCTGCGAGAC
 6 Orf_virus_NZ2                                98.5%  88.2%      TGCGGATATTCAACTCGCACCACCCGCTCTGGCCGCGACACGGCTCGAACGTCTGCTTCGCGCTGCGGTTGCTGCGAGAC
 7 Orf_virus_YX                                 98.1%  95.5%      TGCGGATATTCAACTCGCACCACCCGCTCTGGCCGCGACACGGCTCGAACGTCTGCTTCGCGCTGCGGTTGCTGCGAGAC
 8 Orf_virus_GO                                 98.8%  96.4%      TGCGGATATTCAACTCGCACCACCCGCTCTGGCCGCGACACGGCTCGAACGTCTGCTTCGCGCTGCGGTTGCTGCGAGAC
 9 Orf_virus_B029                               91.8%  81.7%      TTCGGATATTCAACTCGCACCACCCGCTCTGGCCGCGACACGGCTCGAACGTCTGCTTCGCGCTGCGGTTGCTGCGAGAC
10 Orf_virus_IHUMI-1                            78.5%  70.9%      TGCGGATATTCAACTCGCACCACCCGCTCTGGCCGCGACACGGCTCGAACGTCTGCTTCGCGCTGCGGTTGCTGCGAGAC
11 Orf_virus_SJ1                                77.2%  74.2%      TGCGGATATTCAACTCGCACCACCCGCTCTGGCCGCGACACGGCTCGAACGTCTGCTTCGCGCTGCGGTTGCTGCGAGAC
12 Orf_virus_NP                                 24.1%  23.5%      --------------------------------------------------------------------------------
   consensus/100%                                                 ................................................................................
   consensus/90%                                                  TsCGGATATTCAACTCGCACCACCCGCTCTGGCCGCGACACGGCTCuAACGTCTGCTTCGCGCTGCGGTTGCTGCGAGAC
   consensus/80%                                                  TGCGGATATTCAACTCGCACCACCCGCTCTGGCCGCGACACGGCTCGAACGTCTGCTTCGCGCTGCGGTTGCTGCGAGAC
   consensus/70%                                                  TGCGGATATTCAACTCGCACCACCCGCTCTGGCCGCGACACGGCTCGAACGTCTGCTTCGCGCTGCGGTTGCTGCGAGAC

                                                  cov    pid 641         :         .         .         .         .         .         7         . 720
 1 Orf_virus_OV-SA00_NC_005336.1_115015-122053 100.0% 100.0%      GCGCGCACGGGCGAGCGCGTGGTCGAGCAGATGTTCATAGACGGGCGCTGGCACACCGTGCTGAGGACGTCCTGCGGCAA
 2 Orf_virus_D1701                              98.3%  88.3%      GCGCGCACGGGCGAGCGCGTGGTCGAGCAGATGTTCATAGACGGGCGCTGGCACACCGTGCTGAGGACGTCCTGCGGCAA
 3 Orf_virus_NA1-11                             97.9%  87.5%      GTGCGCACGGGCGAGCGCGTGGTCGAGCAGATGTTCATGGACGGGCGCTGGCACACCGTGCTGAGGACGTCCTGCGGCAA
 4 Orf_virus_OV-HN3_12                          98.1%  87.2%      GTGCGCACGGGCGAGCGCGTGGTCGAGCAGATGTTCATGGACGGGCGCTGGCACACCGTGCTGAGGACGTCCTGCGGCAA
 5 Orf_virus_OV-IA82                            98.6%  88.5%      GTGCGCACGGGCGAGCGCGTGGTCGAGCAGATGTTCATGGACGGGCGCTGGCACACCGTGCTGAGGACGTCCTGCGGCAA
 6 Orf_virus_NZ2                                98.5%  88.2%      GTGCGCACGGGCGAGCGCGTGGTCGAGCAGATGTTCATGGACGGGCGCTGGCACACCGTGCTGAGGACGTCCTGCGGCAA
 7 Orf_virus_YX                                 98.1%  95.5%      GCGCGCACGGGCGAGCGCGTGGTCGAGCAGATGTTCATAGACGGGCGCTGGCACACCGTGCTGAGGACGTCCTGCGGCAA
 8 Orf_virus_GO                                 98.8%  96.4%      GCGCGCACGGGCGAGCGCGTGGTCGAGCAGATGTTCATAGACGGGCGCTGGCACACCGTGCTGAGGACGTCCTGCGGCAA
 9 Orf_virus_B029                               91.8%  81.7%      GTGCGCACGGGCGAGCGCGTGGTCGAGCAGATGTTCATGGACGGGCGCTGGCACACCGTGTTGAGGACGTCCTGCGGCAA
10 Orf_virus_IHUMI-1                            78.5%  70.9%      GTGCGCACGGGCGAGCGCGTGGTCGAGCAGATGTTCATGGACGGGCGCTGGCACACCGTGCTGAGGACGTCCTGCGGCAA
11 Orf_virus_SJ1                                77.2%  74.2%      GCGCGCACGGGCGAGCGCGTGGTCGAGCAGATGTTCATAGACGGGCGCTGGCACACCGTGCTGAGGACGTCCTGCGGCAA
12 Orf_virus_NP                                 24.1%  23.5%      --------------------------------------------------------------------------------
   consensus/100%                                                 ................................................................................
   consensus/90%                                                  GsGCGCACGGGCGAGCGCGTGGTCGAGCAGATGTTCATuGACGGGCGCTGGCACACCGTGsTGAGGACGTCCTGCGGCAA
   consensus/80%                                                  GsGCGCACGGGCGAGCGCGTGGTCGAGCAGATGTTCATuGACGGGCGCTGGCACACCGTGCTGAGGACGTCCTGCGGCAA
   consensus/70%                                                  GsGCGCACGGGCGAGCGCGTGGTCGAGCAGATGTTCATuGACGGGCGCTGGCACACCGTGCTGAGGACGTCCTGCGGCAA

                                                  cov    pid 721         .         .         :         .         .         .         .         8 800
 1 Orf_virus_OV-SA00_NC_005336.1_115015-122053 100.0% 100.0%      CAAGGTCTGCGTGCCCGCCGACCTCGTGGGCCAGACGAACCTCGAGGAGGTGCCCTTCTGCGACGTGACGCCCGATATCA
 2 Orf_virus_D1701                              98.3%  88.3%      CAAGGTCTGCGTGCCTGCCGACCTCGTGGGCCATACGAACCTCGAGGAGGTGCCCTTCTGCGACGTGACGCCCGAAATCA
 3 Orf_virus_NA1-11                             97.9%  87.5%      CAAGGTCTGCGTGCCTGCCGACCTCGTGGGCCAGACGAACCTCGAGGAGGTGCCCTTCTGCGACGTGACGCCCGAGATCA
 4 Orf_virus_OV-HN3_12                          98.1%  87.2%      CAAGGTCTGCGTGCCTGCCGACCTCGTGGGCCAGACGAACCTCGAGGAGGTGCCCTTCTGCGACGTGACGCCCGAGATCA
 5 Orf_virus_OV-IA82                            98.6%  88.5%      CAAGGTCTGCGTGCCCGCCGACCTCGTGGGCCAGACGAACCTCGAGGAGGTGCCCTTCTGCGACGTGACGCCCGAGATCA
 6 Orf_virus_NZ2                                98.5%  88.2%      CAAGGTCTGCGTGCCCGCCGACCTCGTGGGCCAGACGAACCTCGAGGAGGTGCCCTTCTGCGACGTGACGCCCGAGATCA
 7 Orf_virus_YX                                 98.1%  95.5%      CAAGGTCTGTGTGCCCGCCGACCTCGTGGGCCAGACGAACCTCGAGGAGGTGCCCTTCTGCGACGTGACGCCCGATATCA
 8 Orf_virus_GO                                 98.8%  96.4%      CAAGGTTTGCGTGCCCGCAGACCTCGTGGGCCAGACGAACCTCGAGGAGGTGCCCTTCTGCGACGTGACGCCCGATATCA
 9 Orf_virus_B029                               91.8%  81.7%      CAAGGTCTGCGTGCCCGCCGACCTCGTGGGCCAGACGAACCTCGAGGAGGTGCCCTTCTGCGACGTGACGCCCGAGATCG
10 Orf_virus_IHUMI-1                            78.5%  70.9%      CAAGGTCTGCGTGCCCGCCGACCTCGTGGGCCAGACGAACCTCGAGGAGGTGCCCTTCTGCGACGTGACGCCCGAGATCA
11 Orf_virus_SJ1                                77.2%  74.2%      CAAGGTCTGCGTGCCCGCAGACCTCGTGGGCCAGACGAGCCTCGAGGAGGTGCCCTTCTGCGACGTAACGCCCGAGATCA
12 Orf_virus_NP                                 24.1%  23.5%      --------------------------------------------------------------------------------
   consensus/100%                                                 ................................................................................
   consensus/90%                                                  CAAGGTsTGsGTGCCsGCsGACCTCGTGGGCCAsACGAuCCTCGAGGAGGTGCCCTTCTGCGACGTuACGCCCGAsATCu
   consensus/80%                                                  CAAGGTCTGCGTGCCsGCsGACCTCGTGGGCCAGACGAACCTCGAGGAGGTGCCCTTCTGCGACGTGACGCCCGAsATCA
   consensus/70%                                                  CAAGGTCTGCGTGCCsGCCGACCTCGTGGGCCAGACGAACCTCGAGGAGGTGCCCTTCTGCGACGTGACGCCCGAsATCA

                                                  cov    pid 801         .         .         .         .         :         .         .         . 880
 1 Orf_virus_OV-SA00_NC_005336.1_115015-122053 100.0% 100.0%      TGCGCCGCGCGCTGGCGATCGACCCGCCGTACGAGGCCGTGGCGCACCCGCGCCGCTGCGTGTACGGGGCCATGGACGTC
 2 Orf_virus_D1701                              98.3%  88.3%      TGCGCCGCGCGCTGGCGATCGACCCGCCGTACGAGGCCGTGGCGCACCCGCACCGCTGCGTGTACGGCGCCATGGACGTC
 3 Orf_virus_NA1-11                             97.9%  87.5%      TGCGCCGCGCACTGGCGATCGACCCGCCGTACGAGGCCGTGGCGCACCCGCACCGCTGCGTGTACGGCGCCATGGACGTC
```

```
 4 Orf_virus_OV-HN3_12                                 98.1%  87.2%        TGCGCCGCGCACTGGCGATCGACCCGCCGTACGAGGCCGTGGCGCACCCGCACCGCTGCGTGTACGGCGCCATGGACGTC
 5 Orf_virus_OV-IA82                                   98.6%  88.5%        TGCGCCGCGCACTGGCGATCGACCCGCCGTACGAGGCCGTGGCGCACCCGCGCCGCTGCGTGTACGGCGCCATGGACGTC
 6 Orf_virus_NZ2                                       98.5%  88.2%        TGCGCCGCGCGCTGGCGATCGACCCGCCGTACGAGGCCGTGGCGCACCCGCACCGCTGCGTGTACGGCGCCATGGACGTC
 7 Orf_virus_YX                                        98.1%  95.5%        TGCGCCGCGCGCTGGCGATCAACCCGCCGTACGAGGCCGTGGCGCACCCGCGCCGCTGCGTGTACGGCGCTATGGACGTC
 8 Orf_virus_GO                                        98.8%  96.4%        TGCGCCGCGCGCTGGCGATCAACCCGCCGTACGAGGCCGTGGCGCACCCGCGCCGCTGCGTGTACGGCGCCATGGACGTC
 9 Orf_virus_B029                                      91.8%  81.7%        TGCGCCGCGCGCTGGCGATCGACCCGCCGTACGAGGCCGTGGCGTACCCGCACCGCTGCGTGTACGGCGCCATGGACGTC
10 Orf_virus_IHUMI-1                                   78.5%  70.9%        TGCGCCGCGCGCTGGCGATCGACCCGCCGTACGAAGCCGTGGCGCACCCGCACCGCTGCGTGTACGGCGCCATGGACGTC
11 Orf_virus_SJ1                                       77.2%  74.2%        TGCGCCGCGCGCTGGCGATCAACCCGCCGTACGAGGCCGTGGCGCACCCGCGCCGCTGCGTGTACGGCGTTATGGACGTC
12 Orf_virus_NP                                        24.1%  23.5%        --------------------------------------------------------------------------------
   consensus/100%                                                          ................................................................................
   consensus/90%                                                           TGCGCCGCGCuCTGGCGATCuACCCGCCGTACGAuGCCGTGGCGsACCCGCuCCGCTGCGTGTACGGsGssATGGACGTC
   consensus/80%                                                           TGCGCCGCGCuCTGGCGATCuACCCGCCGTACGAGGCCGTGGCGCACCCGCuCCGCTGCGTGTACGGCGCsATGGACGTC
   consensus/70%                                                           TGCGCCGCGCuCTGGCGATCuACCCGCCGTACGAGGCCGTGGCGCACCCGCuCCGCTGCGTGTACGGCGCCATGGACGTC

                                                        cov    pid  881    .         9         .         .         .         .         :         . 960
 1 Orf_virus_OV-SA00_NC_005336.1_115015-122053        100.0% 100.0%        CGGTGCGCGAACGAGTACCTCGTGTACTGCACCTTCAAGACGGAGCCGGCGCGGCGGAGCACGTCCTCGCCGGGCCC-GG
 2 Orf_virus_D1701                                     98.3%  88.3%        CGGTGCGCGAACGAGTACCTTGTGTACTGCACCTTCAAAACGGAGCCGGCGCGGCGCAACACGTCCTCGCCGGGCCCTAG
 3 Orf_virus_NA1-11                                    97.9%  87.5%        CGGTGCGCGAACGAGTACCTCGTGTACTGCACCTTCAAGACGGAGCCGGCGCGGCGCAGCACGTCCTCGCCGGGCCC-GG
 4 Orf_virus_OV-HN3_12                                 98.1%  87.2%        CGGTGCGCGAACGAGTACCTCGTGTACTGCACCTTCAAGACGGAGCCGGCGCGGCGCAGCACGTCCTCGCCGGGCCC-GG
 5 Orf_virus_OV-IA82                                   98.6%  88.5%        CGGTGCGCGAACGAGTACCTCGTGTACTGCACCTTCAAGACGGAGCCGGCGCGGCGCAGCACGTCCTCGCCGGGCCC-GG
 6 Orf_virus_NZ2                                       98.5%  88.2%        CGGTGCGCGAACGAGTACCTCGTGTACTGCACCTTCAAGACGGAGCCGACACGGCGCAGCACGTCCTCGCCGGGCCC-GG
 7 Orf_virus_YX                                        98.1%  95.5%        CGGTGCGCGAACGAGTACCTCGTGTACTGCACCTTCAAGACGGAGCCGGCGCGGCGCAGTACGTCCTCGCCGGGTCC-GG
 8 Orf_virus_GO                                        98.8%  96.4%        CGGTGCGCGAACGAGTACCTCGTGTACTGTACCTTCAAGACGGAGCCGGCGCGGCGCAGCACGTCCTCGCCGGGCCC-GG
 9 Orf_virus_B029                                      91.8%  81.7%        CGGTGCGCGAACGAGTACCTCGTGTACTGCACCTTCAAGACGGAGCCGGCGCGGCGCAGCACGTCCTCGCCGGGCCC-GG
10 Orf_virus_IHUMI-1                                   78.5%  70.9%        CGGTGCGCGAACGAGTACCTCGTGTACTGCACCTTCAAGACGGAGCCGACACGGCGCAGCACGTCCTCGCCGGGCCC-GG
11 Orf_virus_SJ1                                       77.2%  74.2%        CGGTGCGCGAACGAGTACCTCGTGTACTGCACCTTCAAGACGGAGCCGGCGCGGCGCAGCACGTCCTCGCCGGGCCC-GG
12 Orf_virus_NP                                        24.1%  23.5%        --------------------------------------------------------------------------------
   consensus/100%                                                          ................................................................................
   consensus/90%                                                           CGGTGCGCGAACGAGTACCTsGTGTACTGsACCTTCAAuACGGAGCCGuCuCGGCGsAusACGTCCTCGCCGGGsCC.uG
   consensus/80%                                                           CGGTGCGCGAACGAGTACCTCGTGTACTGCACCTTCAAGACGGAGCCGuCuCGGCGCAGCACGTCCTCGCCGGGCCC.GG
   consensus/70%                                                           CGGTGCGCGAACGAGTACCTCGTGTACTGCACCTTCAAGACGGAGCCGGCGCGGCGCAGCACGTCCTCGCCGGGCCC.GG

                                                        cov    pid  961    .         .         .         0         .         .         .         . 1040
 1 Orf_virus_OV-SA00_NC_005336.1_115015-122053        100.0% 100.0%        ACGGCCCCCTGTCGCCCGCGACTCCGTCGGCCTCGCAGCCCGCGGCCGCGCGCGCCCCGACGACGCCGCAGGAAGTGACC
 2 Orf_virus_D1701                                     98.3%  88.3%        ACGCCCCCCTGTCGCCCGCGACTCCGTCAACCTCGCGGGCCGCGGCCGCGCGCGCCCCGACAACGCCGCAGGAAGTGACC
 3 Orf_virus_NA1-11                                    97.9%  87.5%        ACGGCCCCTTGTCGCCCGCGACTCCGTCGACCTCGCGGGCCGTGGCCGCGCGCGCCCCGACGACGCCGCAGGAAGTGACC
 4 Orf_virus_OV-HN3_12                                 98.1%  87.2%        ACGGCCCCTTGTCGCCCGCGACTCCGTCGACCTCGCGGGCCGTGGCCGCGCGCGCCCCGACGACGCCGCAGGAAGTGACC
 5 Orf_virus_OV-IA82                                   98.6%  88.5%        ACGGCCCCCTGTCGCCCGCGACTCCGTCGACCTCGCGGGCCGCGGCTGCGCGCGCCCCCACGACGCCGCAGGAAGTGGCC
 6 Orf_virus_NZ2                                       98.5%  88.2%        ACAGCCCGCTGTCGCCCGCGACTCCGTCGACCTCGCGGGCCGCGGCCGCGCGCGTCCCCACGACGCCGCAGGAAGTGGCC
 7 Orf_virus_YX                                        98.1%  95.5%        ACGGCCCCCTGTCGCCCGCGACT---TTGATCTCGCAGGCCGCGGCCGCGCGCGCCCCGACGACGCCGCAGGAAGTGACC
 8 Orf_virus_GO                                        98.8%  96.4%        ACGGCCCCCTGTCGCCCGCGACTCCGTCGACTTCGCAGGCCGCGGCCGCGCGCGCCCCAACGACGCCGCAGGAAGTGACA
 9 Orf_virus_B029                                      91.8%  81.7%        ACGGCCCCCTGTCGCCCGCGACTCCGTCGACCTCGCAGGCCGCGGCCGCGCGCGCCCCGACGACGCCGCAGGAAGTGGCC
10 Orf_virus_IHUMI-1                                   78.5%  70.9%        ACAGCCCGCTGTCGCCCGCGACTCCGTCAACCTCGCAGGCCGCGGCCGCGCGCACCCCCACGACGCCTCAGGAAGTGACC
11 Orf_virus_SJ1                                       77.2%  74.2%        ACGGCCCCCTGTCGCCCGCGACTCCGTCGACTTCGCAGGCCGCGGCCGCGCGCGCCCCGACGACGCCGCAGGAAGTGACC
12 Orf_virus_NP                                        24.1%  23.5%        --------------------------------------------------------------------------------
   consensus/100%                                                          ................................................................................
   consensus/90%                                                           ACusCCCssTGTCGCCCGCGACT...TsuussTCGCuGsCCGsGGCsGCGCGCusCCCsACuACGCCsCAGGAAGTGuCs
   consensus/80%                                                           ACuGCCCssTGTCGCCCGCGACTCCGTCuACsTCGCuGGCCGsGGCCGCGCGCGCCCCsACGACGCCGCAGGAAGTGuCC
   consensus/70%                                                           ACGGCCCCCTGTCGCCCGCGACTCCGTCGACCTCGCuGGCCGCGGCCGCGCGCGCCCCsACGACGCCGCAGGAAGTGuCC

                                                        cov    pid 1041    :         .         .         .         .         1         .         . 1120
 1 Orf_virus_OV-SA00_NC_005336.1_115015-122053        100.0% 100.0%        TCGCCGACCACGAAGCTCGTGGAGACCTGTCTGCGCGACGCCCTCGACTGACT-TGACC--AAGGA-CCCACCGTCCACT
 2 Orf_virus_D1701                                     98.3%  88.3%        TCGC---CCACGAGGCTCGTGGAGACCTGTCTGCGCGACGCCCTCGACTGACCCTAACCTGAAGGACCCCACCGCCCACT
 3 Orf_virus_NA1-11                                    97.9%  87.5%        TCGCCGACAACGAAGCTCGTGGAGACCTGTCTGCGCGACGCCCTCGACGGACTCTGACCCGAAGGA-CCCCAC-CTCACT
 4 Orf_virus_OV-HN3_12                                 98.1%  87.2%        TCGCCGACAACGAAGCTCGTGGAGACCTGTCTGCGCGACGCCCTCGACGGACTCTGACCCGAAGGA-CCCCAC-CTCACT
 5 Orf_virus_OV-IA82                                   98.6%  88.5%        TCGCCGACCACGAGGCTCGTGGAGACCTGCCTGCGCGACGCCCTCGACGGACTCTGACCCGAAGGA-CCCACCGTCCACT
 6 Orf_virus_NZ2                                       98.5%  88.2%        TCGCCGACCACGAGGCTCCTGGAGACCTGTCTGCGCGACGCCCTCGACGGACTCTGACCCGAAGGA-CCCACCGTCCACT
 7 Orf_virus_YX                                        98.1%  95.5%        TCGCCGACCACGAAGCTCGTGGAGACCTGTCTGCGCGACGCCCTCGACTGACT-TGACCCAAGGAC-CCCACCGTCCACT
 8 Orf_virus_GO                                        98.8%  96.4%        TCGCCGACCACGAAGCTCGTGGAGACCTGTCTGCGCGACGCCCTCGAC------TGACCCAAAGGA-CCCCACCTCCACT
 9 Orf_virus_B029                                      91.8%  81.7%        TCGCCGACCACGAGGCTCGTGGAGACCTGTCTGCGCGACGCCCTCGACGGACTCTGACCCGAAGGA-CCCCACCTCCACT
10 Orf_virus_IHUMI-1                                   78.5%  70.9%        TCGCCAACCACGAGGCTCGTAGAGACCTGTCTGCGCGACGCCCTCGACGGACTCTGACCCGAAGGA-CCCACCGTCCACT
11 Orf_virus_SJ1                                       77.2%  74.2%        TCGCCGACCACGAAGCTCGTGGAGACCTGTCTGCGCGACGCCCTCGAC------TGACCCGAGGGA-CCCACCGTCCACT
12 Orf_virus_NP                                        24.1%  23.5%        --------------------------------------------------------------------------------
   consensus/100%                                                          ................................................................................
   consensus/90%                                                           TCGC...CsACGAuGCTCsTuGAGACCTGsCTGCGCGACGCCCTCGAC......TuACC..AuGus.CCCssC.ssCACT
   consensus/80%                                                           TCGCCuACsACGAuGCTCGTGGAGACCTGTCTGCGCGACGCCCTCGAC......TGACCsuAuGGA.CCCssC.ssCACT
   consensus/70%                                                           TCGCCGACCACGAuGCTCGTGGAGACCTGTCTGCGCGACGCCCTCGACsGACs.TGACCCuAAGGA.CCCssCssCCACT

                                                        cov    pid 1121    .         .         :         .         .         .         .        2 1200
 1 Orf_virus_OV-SA00_NC_005336.1_115015-122053        100.0% 100.0%        CACATTCCACTGCCAGACAACAGACTCAAGCTTTTTCTGCATCTACCTCACCGATAATTGAATTGTTATAGGACAAACAG
 2 Orf_virus_D1701                                     98.3%  88.3%        CACATTCCACGGCCAGACAACAGACGCAAGCTTTTTTTGCATCTACCTCGCTGATAATTGAATTGTTATAGTACAAACAG
 3 Orf_virus_NA1-11                                    97.9%  87.5%        CACGCACCACTTCCAGACGACAGACTCAGGCTTTTTCTGCATCTACCTCACTGATAATTGAATTGTTATAGGACAAACAG
 4 Orf_virus_OV-HN3_12                                 98.1%  87.2%        CACGCACCACTTCCAGACGACAGACTCAGGCTTTTTCTGCATCTACCTCGCTGATAATTGAATTGTTATAGGACAAACAG
 5 Orf_virus_OV-IA82                                   98.6%  88.5%        CACATTCCACTGCCAGAC----AACTCAAGCTTTTTCTGCATCTACCTCGCTAATAATTGAATTGTTATAGTACAAACAG
 6 Orf_virus_NZ2                                       98.5%  88.2%        CACATTCCACTGCCAGAC----AACTCAAGCTTTTTCTGCATCTACCTCGCTAATAATTGAATTGTTATAGGACAAACAG
 7 Orf_virus_YX                                        98.1%  95.5%        CACGTTCCACTGCCAGACAACAGACTCAAGCTTTTTCTGCATCTACCTCACCGATAATTGAATTGTTATAGGACAAACAG
 8 Orf_virus_GO                                        98.8%  96.4%        CACATTCCACTGCCAGACAACAGACTCAAGCTTTTTCTGCATCTACCTCACCGATAATTGAATTGTTATAGGACAAACAG
 9 Orf_virus_B029                                      91.8%  81.7%        CACGCACCACTGCCAGACAACAGACTCAGGCTTTTTCTGCATCTACCTCGCTGATAATTGAATTGTTATAGGACAAACAG
10 Orf_virus_IHUMI-1                                   78.5%  70.9%        CACATTCCACTGCCAGAC----AACGCAAGCTTTTTCTGCATCTACCTCGCTAATAATTGAATTGTTATAGTACAAACAG
11 Orf_virus_SJ1                                       77.2%  74.2%        CACGTTCCACTGCCAGACAACAGACTCAAGCTTTTTCTGCATCTACCTCACCGATAATTGAATTGTTATAGGACAAACAG
12 Orf_virus_NP                                        24.1%  23.5%        --------------------------------------------------------------------------------
   consensus/100%                                                          ................................................................................
   consensus/90%                                                           CACussCCACssCCAGAC....uACsCAuGCTTTTTsTGCATCTACCTCuCsuATAATTGAATTGTTATAGsACAAACAG
   consensus/80%                                                           CACussCCACTsCCAGAC....uACsCAuGCTTTTTCTGCATCTACCTCuCsuATAATTGAATTGTTATAGsACAAACAG
   consensus/70%                                                           CACussCCACTGCCAGAC....uACTCAuGCTTTTTCTGCATCTACCTCuCsuATAATTGAATTGTTATAGsACAAACAG

                                                        cov    pid 1201    .         .         .         .         :         .         .         . 1280
 1 Orf_virus_OV-SA00_NC_005336.1_115015-122053        100.0% 100.0%        GCGCACTCGAGCACAATGGCGTGTCTTATCGAATGGGTCGACTCCATCTTCAACCGACGCCACCGTAATTTCGGGCCGGA
 2 Orf_virus_D1701                                     98.3%  88.3%        GCGCACTCAAGCACAATGGCGTGTTTTCTCGAATTGTTAGACTCCATCTTCAACCGACGCCACCGTAATTTCAGGCCGGA
 3 Orf_virus_NA1-11                                    97.9%  87.5%        GTGCACTCGAGCACAATGGCGTGTTTTCTCGAATTGTTAGACTCCATCTTCAACAGACACCACCGTAATTTCAGGCCGGA
 4 Orf_virus_OV-HN3_12                                 98.1%  87.2%        GCGCACTCGAGCACAATGGCGTGTTTTCTCGAATTGTTAGACTCTATCTTCAACCGACACCACCGTAATTTCGGGCCGGA
 5 Orf_virus_OV-IA82                                   98.6%  88.5%        GCGCACTCGAGCACAATGGCGTGTTTTATCGAATTGTTAGACTCCATCTTCAACCGACACCACCGTAATTTCGGGCCGGA
 6 Orf_virus_NZ2                                       98.5%  88.2%        GCGCACTCGAGCACAATGGCGTGTTTTATCGAATTGTTAGACTCCATCTTCAACCGACGCCACCGTAATTTCGGGCCGGA
 7 Orf_virus_YX                                        98.1%  95.5%        GCGCACTCGAGCACAATGGCGTGTCTTATCGAATGGGTCGACTCCATCTTCAACCGACACCACCGTAATTTTGGGCCGGA
 8 Orf_virus_GO                                        98.8%  96.4%        GCGCACTCGAGCACAATGGCGTGTCTTATCGAATGGGTCGACTCCATCTTCAACCGACACCACCGTAATTTTGGGCCGGA
 9 Orf_virus_B029                                      91.8%  81.7%        GCGCACTCGAGCACAATGGCGTGTTTTATCGAATTGTTAGACTCCATCTTCAACCGACGCCACCGTAATTTCAGGCCGGA
10 Orf_virus_IHUMI-1                                   78.5%  70.9%        GCGCACTCGAGCACAATGGCGTGTTTTATCGAATTGTTAGACTCCATCTTCAACCGACACCACCGTAATTTCAGGCCGGA
11 Orf_virus_SJ1                                       77.2%  74.2%        GCGCACTCGAGCACAATGGCGTGTCTTATCGAATGGGTCGACTCCATCTTCAACCGACGCCACCGTAATTTCGGGCCGGA
12 Orf_virus_NP                                        24.1%  23.5%        --------------------------------------------------------------------------------
   consensus/100%                                                          ................................................................................
   consensus/90%                                                           GsGCACTCuAGCACAATGGCGTGTsTTsTCGAATsGsTsGACTCsATCTTCAACsGACuCCACCGTAATTTsuGGCCGGA
   consensus/80%                                                           GCGCACTCGAGCACAATGGCGTGTsTTsTCGAATsGsTsGACTCCATCTTCAACCGACuCCACCGTAATTTsuGGCCGGA
   consensus/70%                                                           GCGCACTCGAGCACAATGGCGTGTsTTsTCGAATsGsTsGACTCCATCTTCAACCGACuCCACCGTAATTTCuGGCCGGA

                                                        cov    pid 1281    .         3         .         .         .         .         :         . 1360
 1 Orf_virus_OV-SA00_NC_005336.1_115015-122053        100.0% 100.0%        AGACATGTACAGGCCCTCCGACCCCACCCCGCCCTCCAAATCTCGCACTCCCCGCACCCCGCA---------CCAACACA
 2 Orf_virus_D1701                                     98.3%  88.3%        AGACATGTACAGGCCCTCTGACGCCACCCCGCCCTCTCAATATCGCACGCCTCGCACTCCCCGCACCCCGCACCAACACA
 3 Orf_virus_NA1-11                                    97.9%  87.5%        AGACATGTACAGGCCCTCTGACG---CCCCGCCCCCCAAATCTCACACGCCTCGCACTCCCCG---------------CA
 4 Orf_virus_OV-HN3_12                                 98.1%  87.2%        AGACATGTACAGGCCCTCTGACG---CCCCGCCCCCCAAATCTCACACGCCTCGCACTCCCCG---------------CA
 5 Orf_virus_OV-IA82                                   98.6%  88.5%        GGACATGTACAGGCCCTCTGACG---CCCCGCCCCCCAAATCTCACACGCCTCGCACTCCCCG---------------CA
 6 Orf_virus_NZ2                                       98.5%  88.2%        GGACATGTACAGGCCCTCTGACG---CCCCGCCCCCCAAATCGCACACGCCTCGCACTCCCCG---------------CA
```

```
 7 Orf_virus_YX                                      98.1%  95.5%       AGACATGTACAGGCCCTCCGACCCCACCCCGCCCTCCAAATCTCACACTCCCCGCACCCTGCA---------CCAACACA
 8 Orf_virus_GO                                      98.8%  96.4%       AGACATGTACAGGCCCTCCGACCCCACCCCGCCCTCCAAATCTCGCACTCCCCGCACCCTGCA---------CCAACACA
 9 Orf_virus_B029                                    91.8%  81.7%       GGACATGTACAGGCCCTCTGACG---CCCCGCCCCCCAAATCTCACACGCCTCGCACTCCCCG---------------CA
10 Orf_virus_IHUMI-1                                 78.5%  70.9%       GGACATGTACAGGCCCTCTGACG---CCCCGCCCCCCAAATCTCACACGCCTCGCACCCCCCG---------------CA
11 Orf_virus_SJ1                                     77.2%  74.2%       AGACATGTACAGGCCCTCCGACCCCACCCCGCCCTCCAAATCTCGCACGCCTCGCACCCCGCA---------CCAACACA
12 Orf_virus_NP                                      24.1%  23.5%       --------------------------------------------------------------------------------
   consensus/100%                                                       ................................................................................
   consensus/90%                                                        uGACATGTACAGGCCCTCsGACs...CCCCGCCCsCssAATssCuCACsCCsCGCACsCssCu...............CA
   consensus/80%                                                        uGACATGTACAGGCCCTCsGACs...CCCCGCCCsCCAAATCTCuCACsCCsCGCACsCssCu...............CA
   consensus/70%                                                        uGACATGTACAGGCCCTCsGACs...CCCCGCCCsCCAAATCTCuCACsCCsCGCACsCCsCu...............CA

                                                       cov    pid 1361         .         .         .         4         .         .         .         . 1440
 1 Orf_virus_OV-SA00_NC_005336.1_115015-122053 100.0% 100.0%       CCCCGCGGAACCGGTGTCCTGAACGTCCGCGGCGACACAACTCTTCTCCCATCTGCGGTGCTTGTGTGGACTCCCTGCCG
 2 Orf_virus_D1701                                   98.3%  88.3%       CCCCGCGGAACCAGTGTTCCGGACACCCGCGGCGACACAACTCCTCTCCCATCTGCGGTGCTTATGTGGACTCCCTGCCG
 3 Orf_virus_NA1-11                                  97.9%  87.5%       CCCCGCGGACCCAGTGTCCCGGACACCCGCGGCGACAAAACTCCTCTCCCATCTACGGTGCTTATGTGGACTCCCTGCCG
 4 Orf_virus_OV-HN3_12                               98.1%  87.2%       CCCCGCGGACCCAGTGTCCCGGACACCCGCGGCGACAAAACTCCTCTCCCATCTACGGTGCTTATGTGGACTCCCTGCCG
 5 Orf_virus_OV-IA82                                 98.6%  88.5%       CCCCGCGGACCCAGTGTCCCGGACACCCGCGGCGACAAAGCTCCTCTCCCATCTACGGTGCTTATGTGGACTCCCTGCCG
 6 Orf_virus_NZ2                                     98.5%  88.2%       CCCCGCGGACCCAGTGTCCCGGACACCCGCGGCGACAAAGCTCCTCTCCCATCTACGGCGCTTATGTGGACTCCCTGCCG
 7 Orf_virus_YX                                      98.1%  95.5%       CCCCGCGGAACCAGTGTCCTGAACGTCCGCGGCGACACAACTCTTCTCCCATCTGCGGTGCTTGTGTGGACTCCCTGCCG
 8 Orf_virus_GO                                      98.8%  96.4%       CCCCGCGGAACCAGTGTCCTGAACGTCCGCGGCGACACAACTCTTCTCCCATCTGCGGTGCTTGTGTGGACTCCCTGCCG
 9 Orf_virus_B029                                    91.8%  81.7%       CCCCGCGGACCCAGTGTTCCGGACACCCGCAGCGACAAAGCTCCTCTCCCATCTACGGTGCTTATGTGGACTCCCTGCCG
10 Orf_virus_IHUMI-1                                 78.5%  70.9%       CCCCGCGGACCCAGTGTCCCGGACACCCGCGGCGACACAACTCCTCTCCCATCTACGGTGCTTATGTGGACTCCCTGCCG
11 Orf_virus_SJ1                                     77.2%  74.2%       CCCCGCGGAACCAGTGTCCTGAACGCCCGCGGCGACACAACTCTTCTCCCATCTGCGGTGCTTGTGTGGACTCCCTGCCG
12 Orf_virus_NP                                      24.1%  23.5%       --------------------------------------------------------------------------------
   consensus/100%                                                       ................................................................................
   consensus/90%                                                        CCCCGCGGAsCCuGTGTsCsGuACusCCGCuGCGACAsAuCTCsTCTCCCATCTuCGGsGCTTuTGTGGACTCCCTGCCG
   consensus/80%                                                        CCCCGCGGAsCCAGTGTsCsGuACusCCGCGGCGACAsAuCTCsTCTCCCATCTuCGGTGCTTuTGTGGACTCCCTGCCG
   consensus/70%                                                        CCCCGCGGAsCCAGTGTCCsGuACusCCGCGGCGACAsAuCTCsTCTCCCATCTuCGGTGCTTuTGTGGACTCCCTGCCG

                                                       cov    pid 1441         :         .         .         .         .         5         .         . 1520
 1 Orf_virus_OV-SA00_NC_005336.1_115015-122053 100.0% 100.0%       AGGAACAGAAAGCGGTTCCAGCATCGACACAGTTGTCCCGGAGATTACGAGCAGTGTCAACTCTCGGAAACCATCAGCCT
 2 Orf_virus_D1701                                   98.3%  88.3%       AGGAACAGAAAGCGGTTCTGGCATCAACACAGTTGTCCCGGAGATTACGAGCAGTGTCAACTCCCGAACACCATCAGCCT
 3 Orf_virus_NA1-11                                  97.9%  87.5%       AGGAACAGAAAGCGGTTCCAGAATCAACACAGTTGTCCCGAGGATTACGAGCGGTGTCAACTATCGGACACCATCAGCCT
 4 Orf_virus_OV-HN3_12                               98.1%  87.2%       AGGAACAGAAAGCGGTTCCAGAATCAACACAGTTGTCCCGAGGATTACGAGCGGTGTCAACTATCGGACACCATCAGCCT
 5 Orf_virus_OV-IA82                                 98.6%  88.5%       AGGAACAGAAAGCGGTTCCAGAATCAACACAGTTGTCCCGGAGATTACGAGCGGTGTCAACTCCAGGACACTATCAGCCT
 6 Orf_virus_NZ2                                     98.5%  88.2%       AGGAACAGAAAGCGGTTCCAGAATCAACACAGTTGTCCCGGAGATTACGAGCGGTGTCAACTCTCGGACACCATCAGCCT
 7 Orf_virus_YX                                      98.1%  95.5%       AGGAACAGAAAGCGGTTCCAGCATCGACACAGTTGTCCCGGAGATTACGAGCAGTGTCAACTCTCGGAAACCATCAGCCT
 8 Orf_virus_GO                                      98.8%  96.4%       AGGAACAGAAAGCGGTTCCAGCATCGACACAGTTGTCCCGGAAATTACGAGCAGTGTCAACTCTCGGAAACCATCAGCCT
 9 Orf_virus_B029                                    91.8%  81.7%       AGGAACAGAAAGCGGTTCCAGAATCAACACAGTTGTCCCGGAGATTACGAGCGGTGTCAACTCTCGGACACCATCAGCCT
10 Orf_virus_IHUMI-1                                 78.5%  70.9%       AGAAACAGAAAGCGGTTCCAGAATCAACACAGTTGTCCCGGAGATTACGAGCGGTGTCAACTCTCGGACACCATCAGCCT
11 Orf_virus_SJ1                                     77.2%  74.2%       AGGAACAGAAAGCGGTTCCAGCATCGACACAGTTGTCCCGGAGATTACGAGCAGTGTCGACTCTCGGAAACCATCAGCCT
12 Orf_virus_NP                                      24.1%  23.5%       --------------------------------------------------------------------------------
   consensus/100%                                                       ................................................................................
   consensus/90%                                                        AGuAACAGAAAGCGGTTCsuGsATCuACACAGTTGTCCCGuuuATTACGAGCuGTGTCuACTsssGuAsACsATCAGCCT
   consensus/80%                                                        AGGAACAGAAAGCGGTTCCAGsATCuACACAGTTGTCCCGuuGATTACGAGCuGTGTCAACTssCGGAsACCATCAGCCT
   consensus/70%                                                        AGGAACAGAAAGCGGTTCCAGsATCuACACAGTTGTCCCGGAGATTACGAGCuGTGTCAACTCTCGGAsACCATCAGCCT

                                                       cov    pid 1521         .         .         :         .         .         .         .         6 1600
 1 Orf_virus_OV-SA00_NC_005336.1_115015-122053 100.0% 100.0%       GGAGGCGACGCTACTCACGGTTACAACGACTTCCATCTCCAGCATCTCCAGCATATACAGCTCTAGTAGCTCAGACTCTA
 2 Orf_virus_D1701                                   98.3%  88.3%       GGATGCGACGCTACTCACAGTTACATTGACGTC---------TATCTCCAGCATATCCAGCTCTAGTAGCTCAGACTCTA
 3 Orf_virus_NA1-11                                  97.9%  87.5%       GGAGGCGACGCTACTCACGGTTACAGTGACGTCCATCTCCAGCATCTCCAGCATATCCAGCTCTAGTAGCTCAGACTCTA
 4 Orf_virus_OV-HN3_12                               98.1%  87.2%       GGAGGCGACGCTACTCACGGTTACAGTGACGTCCATCTCCAGCATCTCCAGCATATCCAGCTCTAGTAGCTCAGACTCTA
 5 Orf_virus_OV-IA82                                 98.6%  88.5%       GGAGGCGACGCTACTCACGGTTACCTCGACTTC---------CATCTCCAGCATATCCAGCTCTAGTAGCTCAGACTCTA
 6 Orf_virus_NZ2                                     98.5%  88.2%       GGATGCGACGCTACTCACGGTTACAGTGACGTC---------CATCTCCAGCATATCCAGCTCTAGTAGCTCAGACTCTA
 7 Orf_virus_YX                                      98.1%  95.5%       GGAGGCGACGCTACTCACGGTTACAACGACTTCCATCTCCAGCATCTCCAGCATATACAGCTCTAGTAGCTCAGACTCTA
 8 Orf_virus_GO                                      98.8%  96.4%       GGAGGCGACGCTACTCACGGTTACAACGACTTCTATCTCCAGCATCTCCAGCATATACAGCTCTAGTAGCTCAGACTCTA
 9 Orf_virus_B029                                    91.8%  81.7%       GGATGCGACGCTACTCACGGTTACAGTGACGTC---------CATCTCCAGCATATCCAGCTCCAGTAGCTCAGACTCTA
10 Orf_virus_IHUMI-1                                 78.5%  70.9%       GGATGCGACGCTACTCACGGTTACAGTGACGTC---------CATCTCCAGCATATCCAGCTCTAGTAGCTCAGACTCTA
11 Orf_virus_SJ1                                     77.2%  74.2%       GGAGGCGACGCTACTCACGGTTACAACGACTTCCATCTCCAGCATCTCCAGCATATACAGCTCTAGTAGCTCAGACTCTA
12 Orf_virus_NP                                      24.1%  23.5%       --------------------------------------------------------------------------------
   consensus/100%                                                       ................................................................................
   consensus/90%                                                        GGAsGCGACGCTACTCACuGTTACsssGACsTC.........sATCTCCAGCATATsCAGCTCsAGTAGCTCAGACTCTA
   consensus/80%                                                        GGAsGCGACGCTACTCACGGTTACAssGACsTC.........CATCTCCAGCATATsCAGCTCTAGTAGCTCAGACTCTA
   consensus/70%                                                        GGAsGCGACGCTACTCACGGTTACAusGACsTC.........CATCTCCAGCATATsCAGCTCTAGTAGCTCAGACTCTA

                                                       cov    pid 1601         .         .         .         .         :         .         .         . 1680
 1 Orf_virus_OV-SA00_NC_005336.1_115015-122053 100.0% 100.0%       TCTCTTTGGGGCAGTGCAGACTGTCCATGGTGTCTGCGACATCG------ACCTCCACGACCTTCTCCTCCTCGGAATGA
 2 Orf_virus_D1701                                   98.3%  88.3%       GCTCTCTGGGGCAGTGCAGACTGTCCATGGTGTCCGCGACATCG------ACCTCCACGACCCTATCCTCCTCGGAATGA
 3 Orf_virus_NA1-11                                  97.9%  87.5%       GCTCTCTGGGGCAGTGCAGACTGTCCATGGTGTCCGCGACATCGACCTCCACCTCCACGACCTTCTCCTCCTCGGAATGA
 4 Orf_virus_OV-HN3_12                               98.1%  87.2%       GCTCTCTGGGGCAGTGCAGACTGTCCATGGTGTCCGCGACATCGACCTCCACCTCCACGACCTTCTCCTCCTCGGAATGA
 5 Orf_virus_OV-IA82                                 98.6%  88.5%       GCTCATTGGGGCAGTGCAGACTGTCCATTGTGTCCGCGACATCG------ACCTCCACGACCTTCTCCTACTCGTCCTGA
 6 Orf_virus_NZ2                                     98.5%  88.2%       TCTCTCTGGGGCAGTGCAGACTGTCCATGGTGTCCGCAACATCAACCTCCACCTCCACAACCTTCTCCTCCTCGGAATGA
 7 Orf_virus_YX                                      98.1%  95.5%       GCTCTCTGAGGCAGTGCAGACTGTCCATGGTGTCCGCGACATCG------ACTTCCACGACATTTTCCTCCTCGGAATGA
 8 Orf_virus_GO                                      98.8%  96.4%       GCTCTCTGAGGCAGTGCAGACTGTCCATGGTGTCCGCGACATCG------ACCTCCACGACATTTTCCTCCTCGGAATGA
 9 Orf_virus_B029                                    91.8%  81.7%       TCTCTCTGGGGCAGTGCAGACTGTCCATGGTGTCCGCGACATCGACCTCCACCTCCACGACCTTCTCCTCCTCGGAATGA
10 Orf_virus_IHUMI-1                                 78.5%  70.9%       TCTCTCTGGGGCAGTGCAGACTGTCCATGGTGTCCGCGACATCGACCTCCACCTCCACGACCTTCTCCTCCTCGGAATGA
11 Orf_virus_SJ1                                     77.2%  74.2%       TCTCTTTGGGGCAGTGCAGACTGTCCATGGTGTCTGCGACATCG------ACTTCCACGACATTTTCCTCCTCAGAATGA
12 Orf_virus_NP                                      24.1%  23.5%       --------------------------------------------------------------------------------
   consensus/100%                                                       ................................................................................
   consensus/90%                                                        sCTCssTGuGGCAGTGCAGACTGTCCATsGTGTCsGCuACATCu......ACsTCCACuACssTsTCCTsCTCusssTGA
   consensus/80%                                                        sCTCTsTGuGGCAGTGCAGACTGTCCATGGTGTCsGCGACATCG......ACsTCCACGACsTTsTCCTCCTCGGAATGA
   consensus/70%                                                        sCTCTsTGGGGCAGTGCAGACTGTCCATGGTGTCCGCGACATCG......ACCTCCACGACsTTsTCCTCCTCGGAATGA

                                                       cov    pid 1681         .         7         .         .         .         .         :         . 1760
 1 Orf_virus_OV-SA00_NC_005336.1_115015-122053 100.0% 100.0%       ACGCTACACTTATTTTTGTATAATAGTTTGTATTGAACCTTAGAGACATCCACAAATAGTTAGGAAGCATGAGTAGTTCA
 2 Orf_virus_D1701                                   98.3%  88.3%       GCACCACACTTATTTTTGTATA--AGTTTGTATTGAACCTTAGAGACATCCACAAATAGTTAGGAAGCATGAGTAGTTCA
 3 Orf_virus_NA1-11                                  97.9%  87.5%       GCGCCACACTTATTTTTGTATAATAGTTTGTATTGAACCTTAGAGACATCCACAAATAGTTAGGAAGCATGAGTAGTTCA
 4 Orf_virus_OV-HN3_12                               98.1%  87.2%       GCGCCACACTTATTTTTGTATAATAGTTTGTATTGAACCTTAGAGACATCCACAAATAGTTAGGAAGCATGAGTAGTTCA
 5 Orf_virus_OV-IA82                                 98.6%  88.5%       GCGCCACACTTATTTTTGTATAATAGTTTGTATTGAACCTTAGAGACATCCACAAATAGTTAGGAAGCATGAGTAGTTCA
 6 Orf_virus_NZ2                                     98.5%  88.2%       GCGCCACACTTATTTTTGTATAATAGTTTGTATTGAACCTTAGAGACATCCACAAATAGTTAGGAAGCATGAGTAGTTCA
 7 Orf_virus_YX                                      98.1%  95.5%       ACGCTACACTTATTTTTGTATAATAGTTTGTATTGAACCTTAGATACATCTACAAATAGTTAGGAAGCATGAGTAGTTCA
 8 Orf_virus_GO                                      98.8%  96.4%       ACGCTACACTTATTTTTGTATAATAGTTTGTATTGAACCTTAGATACATCTACAAATAGTTAGGAAGCATGAGTAGTTCA
 9 Orf_virus_B029                                    91.8%  81.7%       GCACCACACTTATTTTTGTATAATAGTTTGTATTGAACCTTAGAGACATCCACAAATAGTTAGGAAGCATGAGTAGTTCA
10 Orf_virus_IHUMI-1                                 78.5%  70.9%       GCGCCACACTTATTTTTGTATAATAGTTTGTATTGAACCTTAGAGACATCCACAAATAGTTAGGAAGCATGAGTAGTTCA
11 Orf_virus_SJ1                                     77.2%  74.2%       ACGCTACGCTTATTTTTGTATAATAGTTTGTATTGAACCTTAGATACATCTACAAATAGTTAAGAAGCATGAGTAGTTCA
12 Orf_virus_NP                                      24.1%  23.5%       --------------------------------------------------------------------------------
   consensus/100%                                                       ................................................................................
   consensus/90%                                                        uCuCsACuCTTATTTTTGTATA..AGTTTGTATTGAACCTTAGAsACATCsACAAATAGTTAuGAAGCATGAGTAGTTCA
   consensus/80%                                                        uCuCsACACTTATTTTTGTATAATAGTTTGTATTGAACCTTAGAsACATCsACAAATAGTTAGGAAGCATGAGTAGTTCA
   consensus/70%                                                        uCGCsACACTTATTTTTGTATAATAGTTTGTATTGAACCTTAGAsACATCsACAAATAGTTAGGAAGCATGAGTAGTTCA

                                                       cov    pid 1761         .         .         .         8         .         .         .         . 1840
 1 Orf_virus_OV-SA00_NC_005336.1_115015-122053 100.0% 100.0%       AGTAACGAGCCCAC------CCCTAAGCCCAAGCCCCCTGCTCCCATGACTCAGGAGGAGTTTAACAAAGAAGTAGAGAA
 2 Orf_virus_D1701                                   98.3%  88.3%       AGTAGCGAAACCACACCTAAGCCCAAGCCTATCCCTGCTCCCCCCATGACTCAGGAGGAGTTCAACAGAGAAGTAGAGAA
 3 Orf_virus_NA1-11                                  97.9%  87.5%       AGTAGCGAGACCAC------GCCCAAGCCTATCCCTGCTCCTCCCATGACTCAGGAGGAGTTCAACAAAGAAGTAAAGAA
 4 Orf_virus_OV-HN3_12                               98.1%  87.2%       AGTAGCGAGACCAC------GCCCAAGCCTATCCCTGCTCCTCCCATGACTCAGGAGGAGTTCAACAAAGAAGTAAAGAA
 5 Orf_virus_OV-IA82                                 98.6%  88.5%       AGTAGCGAGACCACCCCTAAGCCCAAGCCCATCCCTGCTCCTCCCATGACTCAGGAGGAGTTTAACAAAGAAGTGAAGAA
 6 Orf_virus_NZ2                                     98.5%  88.2%       AGTAGCGAGACCACCCCTAAGCCCAAGCCCATCCCTGCTCCTCCCATGACTCAGGAGGAGTTTAACAAAGAAGTGAAGAA
 7 Orf_virus_YX                                      98.1%  95.5%       AGTAACGAGACCAC------CCCTAAGCCC---CCTGCTCCTCCCATGACCCAGGATGAGTTTAACAAAGAAGTAGAGAA
 8 Orf_virus_GO                                      98.8%  96.4%       AGTAACGAGACCAC------CCCTAAGCCC------CCTTCTCCCATGACCCAGGAGGAGTTTAACAAAGAAGTAGAGAA
 9 Orf_virus_B029                                    91.8%  81.7%       AGTAGCGAGACTAC------GCCCAAGCCTATCCCTGCTCCTCCCATGACTCAGGAGGAGTTTAACAAAGAAGTAGAGAA
```

```
10 Orf_virus_IHUMI-1                               78.5%  70.9%       AGTAGCGAGACTAC------GCCCAAGCCCATCCCTGCTCCTCCCATGACTCAGGAGGAGTTTAACAAAGAAGTGAAGAA
11 Orf_virus_SJ1                                   77.2%  74.2%       AGTAACGAGACCAC------CCCTAAGCCC---CCTGCTCCTCCCATGACCCAGGATGAGTTTAACAAAGAAGTAGAGAA
12 Orf_virus_NP                                    24.1%  23.5%       --------------------------------------------------------------------------------
   consensus/100%                                                     ................................................................................
   consensus/90%                                                      AGTAuCGAusCsAC......sCCsAAGCCs......sCTsCsCCCATGACsCAGGAsGAGTTsAACAuAGAAGTuuAGAA
   consensus/80%                                                      AGTAuCGAGACsAC......sCCsAAGCCs...CCssCTsCsCCCATGACsCAGGAsGAGTTsAACAAAGAAGTuuAGAA
   consensus/70%                                                      AGTAuCGAGACCAC......sCCsAAGCCs...CCTGCTCCTCCCATGACsCAGGAGGAGTTsAACAAAGAAGTuuAGAA

                                                    cov    pid  1841  :       .         .         .         .         9         .         .  1920
 1 Orf_virus_OV-SA00_NC_005336.1_115015-122053    100.0% 100.0%       ACGAAGAGAACAAAAAAAGGAAAAATCTAGAACCGTTGAACGTGAGTCAGAAACCGTAACTGTGTCTGCCGACGGAACAG
 2 Orf_virus_D1701                                 98.3%  88.3%       ATGGGAAGAAAAGAAAAGGAAAAATCTAGAATCGTTGAACGTGAGGCGGAAACCGTAACTGTGTCTGCCGACGGAACAG
 3 Orf_virus_NA1-11                                97.9%  87.5%       ACGGGAAGAAAGGAAAAGGGAAAATCTAGAACCGTTGAACGTGAGTCAGAAACCGTAACTGTATCTTCCGACGGATCAG
 4 Orf_virus_OV-HN3_12                             98.1%  87.2%       ACGGGAAGAAAGGAAAAGGAAAAATCTAGAACCGTTGAACGTGAGTCAGAAACCGTAACTGTATCTTCCGACGGATCAG
 5 Orf_virus_OV-IA82                               98.6%  88.5%       ACGAAAAGAACAGAAAAAGGAAAAATCTAGAACCGTTGAACGTGAGTCAGAAACCGTAACTGTATCTTCCGACGGATCAG
 6 Orf_virus_NZ2                                   98.5%  88.2%       ACGAAAAGAACAGAAAAAGGAAAAATCTAGAACCGTTGAACGTGAGTCAGAAACCGTAACTGTATCTTCCGACGGATCAG
 7 Orf_virus_YX                                    98.1%  95.5%       ACGGGAAGAAAGGAAAAGGGAAAATCTAGAACCGTTGAACGTGAGTCAGAAACCGTAACTGTGTCTGGTGACGGAACAG
 8 Orf_virus_GO                                    98.8%  96.4%       ACGGGAAGAAAGGAAAAGGGAAAATCTAGAACCGTTGAACGTGAGTCAGAAACCGTAACTGTGTCTGGTGACGGAACAG
 9 Orf_virus_B029                                  91.8%  81.7%       ACGGGAAGAAAAGAAAAAGGAAAAACATAGAACCGTTGAACATGAGTTAGAAACCGTAACTGTGTCTGCCGACGGAACAG
10 Orf_virus_IHUMI-1                               78.5%  70.9%       ACGAAAAGAACAGAAAAAGGAAAAATCTAGAACCGTTGAACATGAGTTAGAAACCGTAACTGTATCTGCCAACGGAACAG
11 Orf_virus_SJ1                                   77.2%  74.2%       ACGAAGAGAACAAAAAAAGGAAAAATCTAGAACCGTTGAACGTGAGTCAGAAACCGTAACTGTGTCTGGCGACGGAACAG
12 Orf_virus_NP                                    24.1%  23.5%       --------------------------------------------------------------------------------
   consensus/100%                                                     ................................................................................
   consensus/90%                                                      AsGuuuAGAAsuuAAAAuGGAAAAAssTAGAAsCGTTGAACuTGAGssuGAAACCGTAACTGTuTCTsssuACGGAsCAG
   consensus/80%                                                      ACGuuuAGAAsuuAAAAuGGAAAAATCTAGAACCGTTGAACuTGAGTsAGAAACCGTAACTGTuTCTsssGACGGAsCAG
   consensus/70%                                                      ACGuuAAGAAsuGAAAAuGGAAAAATCTAGAACCGTTGAACGTGAGTCAGAAACCGTAACTGTuTCTsssGACGGAsCAG

                                                    cov    pid  1921  .         .         :         .         .         .         .         0  2000
 1 Orf_virus_OV-SA00_NC_005336.1_115015-122053    100.0% 100.0%       AGAAAACAAGGACCTACGAGCGCGAGTCTGTGAAAACAACCGAATCAGAAAAGAACAACAATCCGTCAACC---AATGAT
 2 Orf_virus_D1701                                 98.3%  88.3%       AGAGAACAAGGACCTACGAGCGCGAGTCTGAAAAAACAACTGAAACAGAAAAGAACAACTATCCGTCAACC---AATGAT
 3 Orf_virus_NA1-11                                97.9%  87.5%       AGATAAAAAAGACTTACGAGCGCGAGTCTGAGAGAAGAACCGAAACAGAAAAGAACAAC---ACGTCAACC---GATGAT
 4 Orf_virus_OV-HN3_12                             98.1%  87.2%       AGATAAAAAAGACTTACGAGCGCGAGTCTGAGAGAAGAACCGAAACAGAAAAGAACAAC---ACGTCAACC---GATGAT
 5 Orf_virus_OV-IA82                               98.6%  88.5%       AGATAAAAAAGACTTACGAGCGCGAGTCTGAGAGAACAACCGAAACAGAAAAGAACAAC---ACGTCAACCGATGATGAT
 6 Orf_virus_NZ2                                   98.5%  88.2%       AGATAAAAAAGACTTACGAGCGCGAGTCTGAGAGAACAACCGAAACAGAAAAGAACAAC---ACGTCAACC---GATGAT
 7 Orf_virus_YX                                    98.1%  95.5%       AGAAAACAAGGACCTACGAGCGCGAGTCTGAGAAAACAACCGAAACAGAAAAGAACAACAATCCGTCAACC---AATGAT
 8 Orf_virus_GO                                    98.8%  96.4%       AGAAAACAAGGACCTACGAGCGCGAGTCTGAGAAAACAACCGAAACAGAAAAGAACAACAATCCGTCAACC---AATGAT
 9 Orf_virus_B029                                  91.8%  81.7%       AGAGAACAAGGATCTACGAGCGCGAGTTCTGAAAGAACAACCGAAACAGAAAAGAACAAC---ACGTCAACC------AAT
10 Orf_virus_IHUMI-1                               78.5%  70.9%       AGATAAAAAAGACTTACGAGCGCGAGTCTGAGAGAACAACCGAAACAGAAAAGAACAAC---ACGTCAACC---GATGAT
11 Orf_virus_SJ1                                   77.2%  74.2%       AGAAAACAAGGATCTACGAGCGCGAGTCTGAGAGAACAACCGAAAAAGAAACGAACAACAATCCGTCAACC---AATGAT
12 Orf_virus_NP                                    24.1%  23.5%       --------------------------------------------------------------------------------
   consensus/100%                                                     ................................................................................
   consensus/90%                                                      AGAsAAsAAuGAssTACGAGCGCGAsTCTGsuAuAAsAACsGAAssAGAAAsGAACAAC...sCGTCAACC......uAT
   consensus/80%                                                      AGAsAAsAAuGAssTACGAGCGCGAGTCTGAuAuAAsAACCGAAACAGAAAAGAACAAC...sCGTCAACC...uATGAT
   consensus/70%                                                      AGAsAAsAAuGACsTACGAGCGCGAGTCTGAGAuAACAACCGAAACAGAAAAGAACAAC...sCGTCAACC...uATGAT

                                                    cov    pid  2001  .         .         .         .         :         .         .         .  2080
 1 Orf_virus_OV-SA00_NC_005336.1_115015-122053    100.0% 100.0%       AATAAGGACAAAGTGACCGACAATCAGCCAAAAGAAGACGATAAGAAACTTGAGGAAACTCCCAAGGACAGTGAGAAGCC
 2 Orf_virus_D1701                                 98.3%  88.3%       AATAAGCAAAATACTTCTGTAGAGAAACCAGAGGAAACT------AAGCCTGCTTCTACTCCTGAAG---GTGAGAAG--
 3 Orf_virus_NA1-11                                97.9%  87.5%       AATAAGCAGAACACCCCTGTAGAGAAACCAGAGGAAACC------AAGCCTGCTTCTACTCCTGAAG---GTGAGAAG--
 4 Orf_virus_OV-HN3_12                             98.1%  87.2%       AATAAGCAGAACACCCCTGTAGAGAAACCAGAGGAAACC------AAGCCTGCTTCTACTCCTGAAG---GTGAGAAG--
 5 Orf_virus_OV-IA82                               98.6%  88.5%       AATAAGCAGAACACCCCTGTAGAGAAACCAGAGGAAACT------AAGCCTGCTTCTACTCCTGAAG---GTGAGAAG--
 6 Orf_virus_NZ2                                   98.5%  88.2%       AATAAGCAGAACACCCCTGTAGAGAAACCAGAGGAAACC------AAGCCTGCTTCTACTCCTGAAG---GTGTGAAG--
 7 Orf_virus_YX                                    98.1%  95.5%       AATAAGGACAAAGTGACCGACAACCAGCCAAAAGAAGACGATAAAAAACCTGAGGAAACT---AAGGACAGTGAGAAGCC
 8 Orf_virus_GO                                    98.8%  96.4%       AATAAGGACAAAGTGACCGACAATCAGCCAAAAGAAGACGATAAGAAACCTGAGGAAACT---AAGGACAGTGAGAAGCC
 9 Orf_virus_B029                                  91.8%  81.7%       GATAAGCAGAACACCCCTGTAGAGAAACCAGAGGAAACT------AAGCCTGCTTCTACTCCTGAAG---GTGATAAG--
10 Orf_virus_IHUMI-1                               78.5%  70.9%       AATAAGCAGAATACTCCTGTAGAGAAACCAGAAGAAACT------AATCCTGCTTCTACTCCTAAAG---GTGAGAAG--
11 Orf_virus_SJ1                                   77.2%  74.2%       AATAAGGACAAAGTGACCGACAATCAGCCAAAAGAAGACGATAAGAAACTTGAGGAACCTCCTAAGGACGGTGAGAAGCC
12 Orf_virus_NP                                    24.1%  23.5%       --------------------------------------------------------------------------------
   consensus/100%                                                     ................................................................................
   consensus/90%                                                      uATAAGsAsAAsusssCsGssuAssAuCCAuAuGAAuss......AAsCsTGssssssCT...uAuG...GTGssAAG..
   consensus/80%                                                      AATAAGsAsAAsusssCsGssuAssAuCCAuAuGAAuss......AAuCsTGsssssACT...uAuG...GTGAGAAG..
   consensus/70%                                                      AATAAGsAsAAsusssCsGssuAssAuCCAuAuGAAuss......AAuCCTGsssssACTCCsuAuG...GTGAGAAG..

                                                    cov    pid  2081  .         1         .         .         .         .         :         .  2160
 1 Orf_virus_OV-SA00_NC_005336.1_115015-122053    100.0% 100.0%       TACACCAACTGAAAAGCCCGCAAACACCTCTTCTGGTGA---------TAATAAGAAAGAGGGGGAAGAAGCAACCCTTG
 2 Orf_virus_D1701                                 98.3%  88.3%       ----CCAGCCGAAACTCCTG------CCCCGACTACTGACCCCCAACCCACCACACAACCGCCTGCAGAATCAAACTCTG
 3 Orf_virus_NA1-11                                97.9%  87.5%       ----CCAGCCGAAACTCCTG------CCCCGACCACTGACCCCCAACCCACTACACAACCACCCGCAGAATCAAACTCTG
 4 Orf_virus_OV-HN3_12                             98.1%  87.2%       ----CCAGCCGAAACTCCTG------CCCCGACCACTGACCCCCAACCCACTACACAACCACCCGCAGAATCAAACTCTG
 5 Orf_virus_OV-IA82                               98.6%  88.5%       ----CCAGCTGAAACTCCTG------CCCCGACTACTGACCCCAACCCACTACACAACCACCCGCAGAATCAGGCCCTG
 6 Orf_virus_NZ2                                   98.5%  88.2%       ----CCAGCCGAGACTCCTG------CCCCGACTACTGACCCCCAACCTACTACACAACCACCCGCAGAATCAAACCCTG
 7 Orf_virus_YX                                    98.1%  95.5%       TACACCAACTGAAAAGCCCGCAGACACCTCTTCTGGTGA---------TAATAAGAAAGAGGGGGAAGAAACAACCCTTA
 8 Orf_virus_GO                                    98.8%  96.4%       TACGCCAACTGAAAAGCCCGCAGACACCTCTTCTGGTGA---------TAATAAGAAAGAGGGGGGAGAAGCAACCCTTG
 9 Orf_virus_B029                                  91.8%  81.7%       ----CCAGCCGAAACTCCTG------CCCCGACTACTAACCCCCAACCCACTACACAACCACCCGCAGAATCGAACCCTG
10 Orf_virus_IHUMI-1                               78.5%  70.9%       ----CCAGCCGAGACTCCTG------CCCCGACTACTGACCCCCAACCCACTACACAACCACCCGCAGAATCAAACCCTG
11 Orf_virus_SJ1                                   77.2%  74.2%       TACACCAACTGAAAAGCCCGCAGACACCTCTTCTGGTGA---------TAATAAGAAAGAGGGGGAAGAAGCAACCCTTG
12 Orf_virus_NP                                    24.1%  23.5%       --------------------------------------------------------------------------------
   consensus/100%                                                     ................................................................................
   consensus/90%                                                      ....CCAuCsGAuAssCCsG......CCsCssCsusTuA.........sAssAsusAAsusssGsAGAAsCuusCssTu
   consensus/80%                                                      ....CCAuCsGAuAssCCsG......CCsCssCsusTGA.........sAsTAsusAAssusssGsAGAAsCAAsCssTG
   consensus/70%                                                      ....CCAuCsGAAAssCCsG......CCsCssCTusTGA.........sAsTAsusAAssusssGsAGAAsCAAsCssTG

                                                    cov    pid  2161  .         .         .         2         .         .         .         .  2240
 1 Orf_virus_OV-SA00_NC_005336.1_115015-122053    100.0% 100.0%       AGAGTCAACCCACGCCTGCACC-TACTCAGCCATCAAACAGCGA--------------AGCAC-----------------
 2 Orf_virus_D1701                                 98.3%  88.3%       GAAGTCAACCCACACCCGCTCCAGAACCAACCCCAGAATCT------------------GAGCCTACACCTGCATCAAAA
 3 Orf_virus_NA1-11                                97.9%  87.5%       GAAGTCAACCCACACCTGCTCCAGAACCAACCCCCGCACCTGAACCTACTCCGGCTCCAGAGCCTACACCTGCATCAGAG
 4 Orf_virus_OV-HN3_12                             98.1%  87.2%       GAAGTCAACCCACACCTGCTCCAGAACCAACCCCCGCACCTGAACCTACTCCGGCTCCAGAGCCTACACCTGCATCAGAG
 5 Orf_virus_OV-IA82                               98.6%  88.5%       GAAGTCAACCCACACCTGTTCCAGAACCAACCCCCGCACCT------------------GAGCCTGCACCGG------AA
 6 Orf_virus_NZ2                                   98.5%  88.2%       GAAGTCAACCCGCACCTGCTTCAGAACCAACCCCCGCACCT------------------GAGCCTGCACCGGAAC-----
 7 Orf_virus_YX                                    98.1%  95.5%       AAAGTCAACCCACGCCTGCACC-TACTCAACCATCAAACAGCGA--------------AGCAC-----------------
 8 Orf_virus_GO                                    98.8%  96.4%       AGAGTCAACCCACGCCTGCACC-TACTCAGCCATCAAACAGCGA--------------AGCAC-----------------
 9 Orf_virus_B029                                  91.8%  81.7%       GAAGTCAACCCACACCTGCTTCAGAACCAACCCCCGCACCT------------------GAGCCTGCACCGG------AA
10 Orf_virus_IHUMI-1                               78.5%  70.9%       GAAGTCAACCCACACCTGCTTCAGAACCAACCCCCGCACCT------------------GAGCCTGCATCAGTAACTCAA
11 Orf_virus_SJ1                                   77.2%  74.2%       AGAGTCAACCCACGCCTGTACC-TACTCAGCCATCAAACAGCGA--------------AGCACCCACTCAACCTCCAAGC
12 Orf_virus_NP                                    24.1%  23.5%       --------------------------------------------------------------------------------
   consensus/100%                                                     ................................................................................
   consensus/90%                                                      uuAGTCAACCCuCuCCsGsssC.sAssCAuCCssssusAsss.................GsuC.................
   consensus/80%                                                      uuAGTCAACCCACuCCTGsssC.sAssCAuCCssCusACss..................GsuC.................
   consensus/70%                                                      uuAGTCAACCCACuCCTGCssC.sAssCAuCCssCusACss..................GsuC.................

                                                    cov    pid  2241  :         .         .         .         .         3         .         .  2320
 1 Orf_virus_OV-SA00_NC_005336.1_115015-122053    100.0% 100.0%       ----------CCACTCAACCTCCAAGCAGTGAGACTCCACCCACT-CAACCTCCAAGC----AG--TACACCCT------
 2 Orf_virus_D1701                                 98.3%  88.3%       CCCACTCCTGCCACTGAGCCCGC---AGC--AACCGAACCCACTTCAGAACCAAAGC--CAAG--TTCTGTCCCTGAGC
 3 Orf_virus_NA1-11                                97.9%  87.5%       CCCACTCCTGCCACTCAGCCTGC-ATCAGT--AACTCAACCCGCTCCAACACCAGAGC--CAAG--TCCAGCCCCTGAGC
 4 Orf_virus_OV-HN3_12                             98.1%  87.2%       CCCACTCCTGCCACTCAGCCTGC-ATCAGT--AACTCAACCCGCTCCAACACCAGAGC--CAAG--TCCAGCCCCTGAGC
 5 Orf_virus_OV-IA82                               98.6%  88.5%       CCCACTCCTGCCACTCAGCCTGC-ATCAGT--AACTCAACCCGCTCCAACACCAGAGC--CAAG--TCCAGCCCCTGAAA
 6 Orf_virus_NZ2                                   98.5%  88.2%       ----------CCACTCAGCCTGC-ATCAGT--AACTCAACCCGCTCCAACACCAGAGC--CAAG--TCCAGCCCCTAAGC
 7 Orf_virus_YX                                    98.1%  95.5%       ----------CCACTCAACCTCCAAGCAGTGAGACTCCATCCACT-CAACCCCCAAGC--AGTGAG--------------
 8 Orf_virus_GO                                    98.8%  96.4%       ----------CCACTCAACCTCCAAGCAGTGAGACTCCATCCACT-CAACCTCCGAGC----AG----------------
 9 Orf_virus_B029                                  91.8%  81.7%       CCCACTCCTGCCACTCAGCCTGC-ATCAGT--AACTCAACCCGCTCCAACACCAGAGC--CAAC--CCCCGCACCTGAGC
10 Orf_virus_IHUMI-1                               78.5%  70.9%       CCTGCTCCTGCCACTCAGCCTGC-ATCAGT--AACCGAACCCACTCCAACACCAGAGC--CAAG--TCCAGCCCCTAAGC
11 Orf_virus_SJ1                                   77.2%  74.2%       AGTACACCATCCACTCAACCTCCAAGCAGTGAGACTCCACCCACT-CAACCTCCAAGCAGTGAGACTCCATCCACTCAAC
12 Orf_virus_NP                                    24.1%  23.5%       --------------------------------------------------------------------------------
```

```
    consensus/100%                                                    ................................................................................
    consensus/90%                                                     ..........CCACTsAuCCssC....AGs..uACsssAsCCuCT.CAusssCsuAGC....ss................
    consensus/80%                                                     ..........CCACTCAuCCTsC.AsCAGT..uACsssAsCCuCT.CAACssCsuAGC....AG................
    consensus/70%                                                     ..........CCACTCAuCCTsC.AsCAGT..uACTCsACCCuCT.CAACssCsuAGC..suAG..ssCsssss......

                                                      cov    pid 2321          .         .         :         .         .         .         .         4 2400
  1 Orf_virus_OV-SA00_NC_005336.1_115015-122053 100.0% 100.0%      ------------------------------------CCACTCAACCTCCAAGCAGTACACCATCCACTGAAC--------
  2 Orf_virus_D1701                              98.3%  88.3%      CTACTCCGGCCTCTGATCCAACCCCTGCA------------------CCAGAACTTACACCAGCCGCAGAAC--------
  3 Orf_virus_NA1-11                             97.9%  87.5%      CTACTCCGGCTCCTGAACCAACCCCTGCACCAGAACCCACTCCCGCTCCAGAACCTACACCATCCACAGAACCGACTCCT
  4 Orf_virus_OV-HN3_12                          98.1%  87.2%      CTACTCCGGCTCCTGAACCAACCCCTGCACCAGAACCCACTCCCGCTCCAGAACCTACACCATCCACAGAACCGACTCCT
  5 Orf_virus_OV-IA82                            98.6%  88.5%      CTACTCCGGCTTCCGAACCAACCCCTGCACCAGAACCCACTCCCGCTCCAAAACCTACACCAGCCACAGAACCGACTCCT
  6 Orf_virus_NZ2                                98.5%  88.2%      CTACTCCGGCTTCTGAACCAACCCCAGCATCTGAGCCTACTTCTGCTCCAGAACCTACACCATCCGCAGAACCAACTCCT
  7 Orf_virus_YX                                 98.1%  95.5%      --------------------------------------------------------ACTCCATCCACTCAAC--------
  8 Orf_virus_GO                                 98.8%  96.4%      -------------------------------------------------------TACACCATCCACTCAAC--------
  9 Orf_virus_B029                               91.8%  81.7%      CTACTCCGGCTTCCGAAC------------------CCACTCCTGCTCCAGAACCTACACCAGCCGCAGAACCGAGTCCT
 10 Orf_virus_IHUMI-1                            78.5%  70.9%      CTACTCCGGCTTCTGAACCAACCCCAGCATCTGAGCCTACTTCTGCTCCAGAACCTACACCATCCGCAGAACCAACTCCT
 11 Orf_virus_SJ1                                77.2%  74.2%      CTCCATCCACTCAACCTCCATCCACTCAACCTCCATCCACTC---------AACCT---CCATCCACTCAACCT------
 12 Orf_virus_NP                                 24.1%  23.5%      --------------------------------------------------------------------------------
    consensus/100%                                                 ................................................................................
    consensus/90%                                                  ...........................................................CCAsCCuCssAAC........
    consensus/80%                                                  .......................................................TACsCCAsCCuCssAAC........
    consensus/70%                                                  ...................................................usssTACACCAsCCuCssAAC........

                                                      cov    pid 2401          .         .         .         .         :         .         .         . 2480
  1 Orf_virus_OV-SA00_NC_005336.1_115015-122053 100.0% 100.0%      -------------------------------CTACTCCAGAGCCAGCTCCTTCTACCGAACCAACAACCAACGCCAACGG
  2 Orf_virus_D1701                              98.3%  88.3%      ----CAACTGTAGAAGCACCACC---------TGCTACAGCACCAACTTCCGAAACCCAACCAGCCACCAACAGCAATCC
  3 Orf_virus_NA1-11                             97.9%  87.5%      CAACCAACCGTAGAAACACCACC------ATCTGCTACAGCACCAACTACCGAAGCCCAGCCA---ACCAA---CAATCC
  4 Orf_virus_OV-HN3_12                          98.1%  87.2%      CAACCAACCGTAGAAACACCACC------ATCTGCTACAGCACCAACTACCGAAGCCCAGCCA---ACCAA---CAATCC
  5 Orf_virus_OV-IA82                            98.6%  88.5%      CAACCAACCGTAGAAACAACACC------ATCTGCTCCAGCACCAACTCCCGAGGCCCAACCACCCGCCAA---CAATCC
  6 Orf_virus_NZ2                                98.5%  88.2%      CAACCAACTGTAGAAACACCACC------ATCTGCTCCAGCACCAACTCCCGAGGCCCAACCACCCGCCAACAGCAATCC
  7 Orf_virus_YX                                 98.1%  95.5%      -------------------------------CTACTCCAGAGCCAGCTCCTTCTACCGAACCAACAACCAACGCCAACGG
  8 Orf_virus_GO                                 98.8%  96.4%      -------------------------------CTACTCCAGAGCCAGCTCCTTCTACCGAACCAACAACCAACGCCAACGG
  9 Orf_virus_B029                               91.8%  81.7%      CAACCAACCGTAGAAACAACACC------ATCTGCACCAGCACCAACTACCGAAGCCCAGCCA---ACCAA---CAATCC
 10 Orf_virus_IHUMI-1                            78.5%  70.9%      CAACCAACTGTAGAAACACCACC------ATCTGCTCCAGCACCAACTCCCGAGGCCCAACCACCCGCCAACAGCAATCC
 11 Orf_virus_SJ1                                77.2%  74.2%      ---CCAAGCAGTGAGACTCCATCCACTGAACCTACTCCAGAGCCAGCTCCTTCCACCGAACCAACAACCAACGCCAACGG
 12 Orf_virus_NP                                 24.1%  23.5%      --------------------------------------------------------------------------------
    consensus/100%                                                 ................................................................................
    consensus/90%                                                  ................................TuCssCAGsuCCAuCTsCssssuCCsAuCCA...uCCAA...CAAsss
    consensus/80%                                                  ...............................CTuCTsCAGsuCCAuCTsCssssuCCsAuCCA...uCCAA...CAAsss
    consensus/70%                                                  ...............................CTuCTsCAGsuCCAuCTsCssssuCCsAuCCA...uCCAA...CAAsss

                                                      cov    pid 2481          .         5         .         .         .         .         :         . 2560
  1 Orf_virus_OV-SA00_NC_005336.1_115015-122053 100.0% 100.0%      TACACCTGCCGCCACTACTTCAGAAACCACTGCCTAAATGAGTCCGTAAGCATTTCAGAGTAACGTACACTAGTAAGCGA
  2 Orf_virus_D1701                              98.3%  88.3%      CGCTACCGAA---ACCACTGGTGCCAGCACCTCCTAAATTAGTCCGTAAGCATTTCAGAGTAACGTACCTTAGCAAGCGC
  3 Orf_virus_NA1-11                             97.9%  87.5%      CACTACTGAAACCACCACTGGTACCAGCACTTCTTAAATGAGACC-TAAGCATTTCAGAGTAAC----CGTAGTAAGCGA
  4 Orf_virus_OV-HN3_12                          98.1%  87.2%      CACTACTGAAACCACCACTGGTACCAGCACTTCTTAAATGAGACC-TAAGCATTTCAGAGTAAC----CGTAGTAAGCGA
  5 Orf_virus_OV-IA82                            98.6%  88.5%      CACTACTGAAACTACCACTGGTACCAGCACCTCCTAAGTGAGTACGTAAGCATTTCGGAGTAACGT--CGTAGCAAGCGC
  6 Orf_virus_NZ2                                98.5%  88.2%      CACTACTGAAACTACCACTGGTACCAGCACCTCCTAAGTGAGTACGTAAGCATTTCGGAGTAACGT--CGTAGCAAGCGC
  7 Orf_virus_YX                                 98.1%  95.5%      TACACCTGCCGCCACTACTTCAGAAACCACTGCCTAAATGAGTCCGCAAGCATTTCA---TAACGTA-CGTAGTAAGCGA
  8 Orf_virus_GO                                 98.8%  96.4%      TACACCTGCCGCCACTACTTCAGAAACCACTGCCTAAATGAGTCCGCAAGCATTTCA---TAACGTA-CGTAGTAAGCGA
  9 Orf_virus_B029                               91.8%  81.7%      CACTACTGAAACTACCACTGGTACCAGCACTTCTTAAGTGAGTCCGTAAGCATTTCAGAGTAACGT--CGTAGCAAGCGC
 10 Orf_virus_IHUMI-1                            78.5%  70.9%      CACTACTGAAACTACCACTGGTACCAGCACCTCCTAAGTGAGTACGTAAGCATTTCAGAGTAACGT--CGTAGCAAGCGC
 11 Orf_virus_SJ1                                77.2%  74.2%      TACACCTGCCGCCACTACTTCAGAAACCACTGCCTAAATGAGTCTGTAAGCATTTCAGAGTAACGT--ACTAGTAAGCGA
 12 Orf_virus_NP                                 24.1%  23.5%      --------------------------------------------------------------------------------
    consensus/100%                                                 ................................................................................
    consensus/90%                                                  suCssCsGss...ACsACTsssussAsCACssCsTAAuTsAGsss.sAAGCATTTCu...TAAC....ssTAGsAAGCGs
    consensus/80%                                                  sACssCTGssuCsACsACTsssussAsCACssCsTAAuTGAGssC.sAAGCATTTCu...TAAC....ssTAGsAAGCGs
    consensus/70%                                                  sACssCTGssuCsACsACTsssussAsCACssCsTAAuTGAGTsCGTAAGCATTTCAGAGTAACGT..CsTAGsAAGCGs

                                                      cov    pid 2561          .         .         .         6         .         .         .         . 2640
  1 Orf_virus_OV-SA00_NC_005336.1_115015-122053 100.0% 100.0%      TAGTCCGCCGCGAGCGGTTCTCGCAAGTTTTTTCGGGTAAAAAGCGTACACCGTCGCCTTGTCGCGGCGGTGTACGCTTT
  2 Orf_virus_D1701                              98.3%  88.3%      TAGTCCGCCGCGAGCGGTTCTCGCAAGTTTTTTCGGGTAAAAAGCGTACACCGTCGCCTTGTCGCGGCGGTGTACGCTTT
  3 Orf_virus_NA1-11                             97.9%  87.5%      TAGTCCGCCGCGAGCGGTTCTCGCAAGTTTTTTCGGGTAAAAAGCGTACACCGTCGCCTCGTCGCGGCGGTGTACGCTTT
  4 Orf_virus_OV-HN3_12                          98.1%  87.2%      TAGTCCGCCGCGAGCGGTTCTCGCAAGTTTTTTCGGGTAAAAAGCGTACACCGTCGCCTCGTCGCGGCGGTGTACGCTTT
  5 Orf_virus_OV-IA82                            98.6%  88.5%      TAGTCCGCCGCGAGCGGTTCTTGCAAGTTTTTTCGGGTAAAAAGCGTACACCGTCGCCTTGTAGCGGCGGTGTACGCTTT
  6 Orf_virus_NZ2                                98.5%  88.2%      TAGTCCGCCGCGAGCGGTTCTCGCAAGTTTTTTCGGGTAAAAAGCGTACACCGTCGCCTTGTCGCGGCGGTGTACGCTTT
  7 Orf_virus_YX                                 98.1%  95.5%      TAGTCCGCCGCGAGCGGTTCTCGCAAG-TTTTTCGGGTAAAAAGCGTACACCGTCGCCTTGTCGCGGCGGTGTACGCTTT
  8 Orf_virus_GO                                 98.8%  96.4%      TAGTCCGCCGCGAGCGGTTCTCGCAAG-TTTTTCGGGTAAAAAGCGTACACCGTCGCCTTGTCGCGGCGGTGTACGCTTT
  9 Orf_virus_B029                               91.8%  81.7%      TAGTCCGCCGCGAGCGGTTCTCGCAAGTTTTTTCGGGTAAAAAGCGTACACCGTCGCCTTGTCGCGGCGGTGTACGCTTT
 10 Orf_virus_IHUMI-1                            78.5%  70.9%      TAGTCCGCCGCGAGCGGTTCTCGCAAGTTTTTTCGGGTAAAAAGCGTACACCGTCGCCTTGTAGCGGCGGTGTACGCTTT
 11 Orf_virus_SJ1                                77.2%  74.2%      TAGTCCGCCGCGAGCGGTTCTCGCAAG-TTTTTCGGGTAAAAAGCGTACACCGTCGCCTTGTTGCGGCGGTGTACGCTTT
 12 Orf_virus_NP                                 24.1%  23.5%      --------------------------------------------------------------------------------
    consensus/100%                                                 ................................................................................
    consensus/90%                                                  TAGTCCGCCGCGAGCGGTTCTsGCAAG.TTTTTCGGGTAAAAAGCGTACACCGTCGCCTsGTsGCGGCGGTGTACGCTTT
    consensus/80%                                                  TAGTCCGCCGCGAGCGGTTCTCGCAAG.TTTTTCGGGTAAAAAGCGTACACCGTCGCCTsGTsGCGGCGGTGTACGCTTT
    consensus/70%                                                  TAGTCCGCCGCGAGCGGTTCTCGCAAG.TTTTTCGGGTAAAAAGCGTACACCGTCGCCTTGTsGCGGCGGTGTACGCTTT

                                                      cov    pid 2641          :         .         .         .         .         7         .         . 2720
  1 Orf_virus_OV-SA00_NC_005336.1_115015-122053 100.0% 100.0%      TTTCACGCCCTTTTTGC-AAATTTAAATTGTACCTGCGCTGGCTCTAGGAGAGATGGCGTGCCTCCGGGTGTTCCTGGCG
  2 Orf_virus_D1701                              98.3%  88.3%      TTTCACGCCCTTTTTGCAAAATTTAAATTGTACCCGCGCCGGCTCTAGGAGAGATGGCGTGCCTCAGAGTGTTTTTGGCG
  3 Orf_virus_NA1-11                             97.9%  87.5%      TTTCACGCCCTTTTTGCAAAATTTAAATTGTACCCGCGCCGGCTCTAGGAAAGATGGCGTGCCTCAGAGTGTTCCTGGCG
  4 Orf_virus_OV-HN3_12                          98.1%  87.2%      TTTCACGCCCTTTTTGCAAAATTTAAATTGTACCCGCGCCGGCTCTAGGAAAGATGGCGTGCCTCAGAGTGTTCCTGGCG
  5 Orf_virus_OV-IA82                            98.6%  88.5%      TTTCACGCCCTTTTTGCAAAATTTAAATTGTACCCGCGCCGGCTCTAGGAAAGATGGCGTGCCTCAGGGTGTTCTTGGCG
  6 Orf_virus_NZ2                                98.5%  88.2%      TTTCACGCCCTTTTTGCAAAATTTAAATTGTACCCGCGCCGGCTCTAGGAAAGATGGCGTGCCTCAGAGTGTTCCTGGCG
  7 Orf_virus_YX                                 98.1%  95.5%      TTTCACGCCCTTTTTGC-AAATTTAAATTGTACCTGCGCTGGCTCTAAGAGAGATGGCGTGCATCCGGGTGTTCCTGGCG
  8 Orf_virus_GO                                 98.8%  96.4%      TTTCACGCCCTTTTTGC-AAATTTAAATTGTACCTGCACTGGCTCTAGGAGAGATGGCGTGCCTCCGGGTGTTCCTAGCG
  9 Orf_virus_B029                               91.8%  81.7%      TTTCACGCCCTTTTTGCAAAATTTAAATTGTACCCGCGCCGGCTATAGGAAAGATGGCGTGTCTCAGGGTGTTCCTGGCG
 10 Orf_virus_IHUMI-1                            78.5%  70.9%      TTTCACGCCCTTTTTGCAAAATTTAAATTGTACCCGCGCCGGCTCTAGGAGAGATGGCGTGCCTCAGAGTGTTCCTGGCG
 11 Orf_virus_SJ1                                77.2%  74.2%      TTTCACGCCCTTTTTGCAAAATTTAAATTGTACCCGCGCTGGCTCTAAGAGAGATGGCGTGCCTCCGGGTGTTCCTGGCG
 12 Orf_virus_NP                                 24.1%  23.5%      --------------------------------------------------------------------------------
    consensus/100%                                                 ................................................................................
    consensus/90%                                                  TTTCACGCCCTTTTTGC.AAATTTAAATTGTACCsGCuCsGGCTsTAuGAuAGATGGCGTGssTCsGuGTGTTssTuGCG
    consensus/80%                                                  TTTCACGCCCTTTTTGC.AAATTTAAATTGTACCsGCGCsGGCTCTAuGAuAGATGGCGTGCCTCsGuGTGTTCsTGGCG
    consensus/70%                                                  TTTCACGCCCTTTTTGC.AAATTTAAATTGTACCsGCGCsGGCTCTAGGAuAGATGGCGTGCCTCsGuGTGTTCCTGGCG

                                                      cov    pid 2721          .         .         :         .         .         .         .         8 2800
  1 Orf_virus_OV-SA00_NC_005336.1_115015-122053 100.0% 100.0%      GTGTTCGCGCTGTGCGGAAGCGTGCACTCGGCGCAATGGATCGGCGAGCGCGACTTCTGCATGGCCCACGCGCAGGACGT
  2 Orf_virus_D1701                              98.3%  88.3%      GTGCTCGCGCTGTGCGGGAGCGTGCACTCGGCGCGATGGATCGGCGAGCGCGACTTCTGCATGGCCCACGCGCAGGACGT
  3 Orf_virus_NA1-11                             97.9%  87.5%      GTGCTCGCGCTGTGCGGGAGCGTGCACTCGGCGCAATGGATCGGCGAGCGCGACTTCTGCATGGCCCACGCACAGGACGT
  4 Orf_virus_OV-HN3_12                          98.1%  87.2%      GTGCTCGCGCTGTGCGGGAGCGTGCACTCGGCGCAATGGATCGGCGAGCGCGACTTCTGCATGGCCCACGCACAGGACGT
  5 Orf_virus_OV-IA82                            98.6%  88.5%      GTGCTCGCGCTGTGCGGGAGCGTGCACTCGGCGCAATGGATCGGCGAGCGCGACTTCTGCACGGCCCACGCACAGGACGT
  6 Orf_virus_NZ2                                98.5%  88.2%      GTGCTCGCGCTGTGCGGGAGCGTGCACTCGGCGCAATGGATCGGCGAGCGCGACTTCTGCACGGCCCACGCGCAGGACGT
  7 Orf_virus_YX                                 98.1%  95.5%      GTGTTCGCGCTGTGCGGAAGCGTGCACTCGGCGCAATGGATCGGCGAGCGCGACTTTTGCATGGCCCACGCGCAGGACGT
  8 Orf_virus_GO                                 98.8%  96.4%      GTGTTCGCGCTGTGCGGAAGCGTGCACTCGGCGCAATGGATCGGCGAGCGCGACTTCTGCATGGCCCACGCGCAGGACGT
  9 Orf_virus_B029                               91.8%  81.7%      GTGCTCGCGCTGTGCGGGAGCGTGCACTCGGCGCAATGGATCGGCGAGCGCGACTTCTGCACGGCCCACGCGCAGGACGT
 10 Orf_virus_IHUMI-1                            78.5%  70.9%      GTGCTCGCGCTGTGCGGGAGCGTGCACTCGGCGCAATGGATCGGCGAGCGCGACTTCTGCACGGCCCACGCGCAGGACGT
 11 Orf_virus_SJ1                                77.2%  74.2%      GTGTTCGCGCTGTGCGGAAGCGTGCACTCGGCGCAATGGATCGGCGAGCGCGACTTCTGCATGGCCCACGCGCAGGACGT
 12 Orf_virus_NP                                 24.1%  23.5%      --------------------------------------------------------------------------------
    consensus/100%                                                 ................................................................................
    consensus/90%                                                  GTGsTCGCGCTGTGCGGuAGCGTGCACTCGGCGCuATGGATCGGCGAGCGCGACTTsTGCAsGGCCCACGCuCAGGACGT
    consensus/80%                                                  GTGsTCGCGCTGTGCGGuAGCGTGCACTCGGCGCAATGGATCGGCGAGCGCGACTTCTGCAsGGCCCACGCuCAGGACGT
```

```
   consensus/70%                                                    GTGsTCGCGCTGTGCGGuAGCGTGCACTCGGCGCAATGGATCGGCGAGCGCGACTTCTGCAsGGCCCACGCuCAGGACGT

                                                      cov    pid 2801         .         .         .         .         :         .         .         . 2880
 1 Orf_virus_OV-SA00_NC_005336.1_115015-122053 100.0% 100.0%       CTTCGCGCGGCTGCAAGTGTGGATGCGCATCGACCGGAACGTGACCGCTGCGGATAACAACTCGGCCTGTGCGCTGGCGA
 2 Orf_virus_D1701                              98.3%  88.3%       CTTCGCGCGGCTGCAGGTGTGGATGCGCATCGACCGGAACGTGACCGCCGCGGACAACAGCTCGGCCTGCGCGCTGGCGA
 3 Orf_virus_NA1-11                             97.9%  87.5%       CTTCGCGCGGCTGCAGGTGTGGATGCGCATCGACCGGAACGTAACCGCCGCGGACAACAGCTCGGCCTGCGCGCTGGCGA
 4 Orf_virus_OV-HN3_12                          98.1%  87.2%       CTTCGCGCGGCTGCAGGTGTGGATGCGCATCGACCGGAACGTAACCGCCGCGGACAACAGCTCGGCCTGCGCGCTGGCGA
 5 Orf_virus_OV-IA82                            98.6%  88.5%       CTTCGCGCGGCTGCAGGTGTGGATGCGCATCGACCGGAACGTGACCGCCGCGGACAACAGCTCGGCCTGCGCGCTGGCGA
 6 Orf_virus_NZ2                                98.5%  88.2%       CTTCGCGCGGCTGCAGGTGTGGATGCGCATTGACCGGAACGTGACCGCCGCGGACAACAGCTCGGCCTGCGCGCTGGCGA
 7 Orf_virus_YX                                 98.1%  95.5%       ATTCGCGCGGCTGCAAGTGTGGATGCGCGTCGACCGGAACGTGACTGCTGCGGATAACAACTCGGCCTGCGCGCTGGCGA
 8 Orf_virus_GO                                 98.8%  96.4%       ATTCGCGCGGCTGCAAGTGTGGATGCGCGTCGACCGGAACGTGACTGCTGCGGATAACAACTCGGCCTGCGCGCTGGCGA
 9 Orf_virus_B029                               91.8%  81.7%       CTTCGCGCGGCTGCAGGTGTGGATGCGCATCGACCGGAACGTGACCGCCGCGGACAACAGCTCGGCCTGCGCGCTGGCGA
10 Orf_virus_IHUMI-1                            78.5%  70.9%       CTTCGCGCGGCTGCAGGTGTGGATGCGCATCGACCGGAACGTGACAGCCGCGGACAACAGCTCGGCTTGCGCGCTGGCGA
11 Orf_virus_SJ1                                77.2%  74.2%       CTTCGCGCGGCTGCAAGTGTGGATGCGCATCGACCGTAACGTGACCGCTGCGGATAACAACTCGGCCTGCGCGCTGGCGA
12 Orf_virus_NP                                 24.1%  23.5%       --------------------------------------------------------------------------------
   consensus/100%                                                   ................................................................................
   consensus/90%                                                    sTTCGCGCGGCTGCAuGTGTGGATGCGCuTsGACCGsAACGTuACsGCsGCGGAsAACAuCTCGGCsTGsGCGCTGGCGA
   consensus/80%                                                    sTTCGCGCGGCTGCAuGTGTGGATGCGCuTCGACCGGAACGTuACsGCsGCGGAsAACAuCTCGGCCTGCGCGCTGGCGA
   consensus/70%                                                    CTTCGCGCGGCTGCAuGTGTGGATGCGCATCGACCGGAACGTGACsGCsGCGGAsAACAuCTCGGCCTGCGCGCTGGCGA

                                                      cov    pid 2881         .         9         .         .         .         .         :         . 2960
 1 Orf_virus_OV-SA00_NC_005336.1_115015-122053 100.0% 100.0%       TAGAGACGCCGCCAAGCAACTTCGACGCGGACGTCTACGTCGCCGCGGCCGGCATAAACGTCAGCGTGTCCGCGATCAAC
 2 Orf_virus_D1701                              98.3%  88.3%       TAGAGACGCCGCCGAGCAACTTCGACGTGGACGTCTACGTCGCCGCGGCCGGCATAAACGTCAGCGTGTCCGCGATCAAC
 3 Orf_virus_NA1-11                             97.9%  87.5%       TAGAAACGCCGCCGAGCAACTTCGACGCGGACGTCTACGTCGCCGCGGCCGGCATAAACGTCAGCGTGTCCGCAATCAAC
 4 Orf_virus_OV-HN3_12                          98.1%  87.2%       TAGAAACGCCGCCGAGCAACTTCGACGCGGACGTCTACGTCGCCGCGGCCGGCATAAACGTCAGCGTGTCCGCAATCAAC
 5 Orf_virus_OV-IA82                            98.6%  88.5%       TAGAGACGCCGCCGAGCAACTTCGACGCGGACGTCTACGTCGCCGCGGCCGGCATAAACGTCAGCGTGTCCGCGATCAAC
 6 Orf_virus_NZ2                                98.5%  88.2%       TAGAGACGCCGCCGAGCAACTTCGACGCGGACGTCTACGTCGCCGCGGCCGGCATAAACGTCAGCGTGTCCGCGATCAAC
 7 Orf_virus_YX                                 98.1%  95.5%       TAGAGACGCCGCCGAGCAACTTCGACGCGGACGTCTACGTCGCCGCGGCCGGCATAAACGTCAGCGTGTCCGCGATCAAC
 8 Orf_virus_GO                                 98.8%  96.4%       TAGAGACGCCGCCGAGCAACTTCGACGCGGACGTCTACGTCGCCGCGGCCGGCATAAACGTCAGCGTGTCCGCGATCAAC
 9 Orf_virus_B029                               91.8%  81.7%       TAGAAACACCGCCGAGCAACTTCGACGCGGACGTCTACGTCGCCGCGGCCGGCATAAACGTCAGCGTGGCCGCGATCAAC
10 Orf_virus_IHUMI-1                            78.5%  70.9%       TAGAGACGCCGCCGAGCAACTTCGACGCGGACGTCTACGTCGCCGCGGCCGGCATAAACGTCAGCGTGTCCGCTATCAAC
11 Orf_virus_SJ1                                77.2%  74.2%       TAGAGACGCCGCCGAGCAACTTCGACGCGGACGTCTACGTCGCCGCGGCCGGCATAAACGTCAGCGTGTCCGCGATCAAC
12 Orf_virus_NP                                 24.1%  23.5%       --------------------------------------------------------------------------------
   consensus/100%                                                   ................................................................................
   consensus/90%                                                    TAGAuACuCCGCCuAGCAACTTCGACGsGGACGTCTACGTCGCCGCGGCCGGCATAAACGTCAGCGTGsCCGCsATCAAC
   consensus/80%                                                    TAGAuACGCCGCCGAGCAACTTCGACGCGGACGTCTACGTCGCCGCGGCCGGCATAAACGTCAGCGTGTCCGCuATCAAC
   consensus/70%                                                    TAGAuACGCCGCCGAGCAACTTCGACGCGGACGTCTACGTCGCCGCGGCCGGCATAAACGTCAGCGTGTCCGCuATCAAC

                                                      cov    pid 2961         .         .         .         0         .         .         .         . 3040
 1 Orf_virus_OV-SA00_NC_005336.1_115015-122053 100.0% 100.0%       TGCGGCTTCTTCAGCATGCGCCAGGTAGAGACAACGTACGACACGGCACGCCGGCAGATGTACGTGTACATGGACACCTG
 2 Orf_virus_D1701                              98.3%  88.3%       TGCGGCTTCTTCAACATGCGCCAGGTAGAGACAACGTACAACACGGCACGCCGGCAGATGTACGTCTACATGGACACCTG
 3 Orf_virus_NA1-11                             97.9%  87.5%       TGCGGCTTCTTCAACATGCGCCAGGTAGAGACGACGTACAACACGGCACGCCGGCAGATGTACGTGTACATGGACACCTG
 4 Orf_virus_OV-HN3_12                          98.1%  87.2%       TGCGGCTTCTTCAACATGCGCCAGGTAGAGACGACGTACAACACGGCACGCCGGCAGATGTACGTGTACATGGACACCTG
 5 Orf_virus_OV-IA82                            98.6%  88.5%       TGCGGCTTCTTCAACATGCGCCAAGTAGAGACAACGTACAACACGGCACGCCGGCAGATGTACGTGTACATGGACTCTTG
 6 Orf_virus_NZ2                                98.5%  88.2%       TGCGGCTTCTTCAACATGCGCCAGGTAGAGACGACGTACAACACGGCACGCCGGCAGATGTACGTGTACATGGACTCCTG
 7 Orf_virus_YX                                 98.1%  95.5%       TGCGGCTTCTTCAGCATGCGCCAGGTAGAGACAACGTACGACACGGCACGCCGGCAGATGTACGTGTACATGGACACCTG
 8 Orf_virus_GO                                 98.8%  96.4%       TGCGGCTTCTTCAGCATGCGCCAGGTAGAGACAACGTACAACACAGCACGCCGGCAGATGTACGTGTACATGGACACCTG
 9 Orf_virus_B029                               91.8%  81.7%       TGCGGCTTCTTCAACATGCGCCAGGTAGAGACGACGTACAACACGGCACGCCGGCAGATGTACGTGTACATGGACTCCTG
10 Orf_virus_IHUMI-1                            78.5%  70.9%       TGCGGCTTCTTCAACATGCGCCAAGTAGAGACGACGTACAACACGGCACGCCGGCAGATGTACGTGTACATGGACTCCTG
11 Orf_virus_SJ1                                77.2%  74.2%       TGCGGCTTCT----------------------------------------------------------------------
12 Orf_virus_NP                                 24.1%  23.5%       --------------------------------------------------------------------------------
   consensus/100%                                                   ................................................................................
   consensus/90%                                                    TGCGGCTTCT......................................................................
   consensus/80%                                                    TGCGGCTTCTTCAuCATGCGCCAuGTAGAGACuACGTACuACACuGCACGCCGGCAGATGTACGTsTACATGGACsCsTG
   consensus/70%                                                    TGCGGCTTCTTCAuCATGCGCCAuGTAGAGACuACGTACuACACGGCACGCCGGCAGATGTACGTGTACATGGACsCCTG

                                                      cov    pid 3041         :         .         .         .         .         1         .         . 3120
 1 Orf_virus_OV-SA00_NC_005336.1_115015-122053 100.0% 100.0%       GGACCCTTGGGTGCTCGACGACCCCCAGCCGCTCTTCAGCCAGGAGTACGAAAACGAAACGCTGCCGTACCTGCTGGAGG
 2 Orf_virus_D1701                              98.3%  88.3%       GGACCCCTGGGTGCTCAACGACCCCCAGCCGCTCTTCAGCCAGGAGCACGAAAACGAAACGCTGCCGTACCTGCTGGAGG
 3 Orf_virus_NA1-11                             97.9%  87.5%       GGACCCCTGGGTGCTCGACGACCCCCAGCCGCTCTTCAGCCAGGAGTACGAAAACGAAACGCTGCCGTACCTGCTGGAGG
 4 Orf_virus_OV-HN3_12                          98.1%  87.2%       GGACCCCTGGGTGCTCGACGACCCCCAGCCGCTCTTCAGCCAGGAGTACGAAAACGAAACGCTGCCGTACCTGCTGGAGG
 5 Orf_virus_OV-IA82                            98.6%  88.5%       GGACCCCTGGATGCTCGACGACCCCCAGCCGCTCTTCAGCCAGGAGTACGAAAACGAAACGCTGCCGTACCTGCTGGAGG
 6 Orf_virus_NZ2                                98.5%  88.2%       GGACCCTTGGGTGATCGACGACCCCCAGCCGCTCTTCAGCCAGGAGTACGAAAACGAAACGCTTCCGTACCTGCTGGAGG
 7 Orf_virus_YX                                 98.1%  95.5%       GGACCCCTGGGTGCTCGACGACCCCCAGCCGCTCTTCAGCCAGGAGTACGAAAACGAAACGCTGCCGTACCTGCTGGAGG
 8 Orf_virus_GO                                 98.8%  96.4%       GGACCCCTGGGTGCTCGACGACCCCCAGCCGCTCTTCAGCCAGGAGTACGAAAACGAAACGCTGCCGTACCTGCTGGAGG
 9 Orf_virus_B029                               91.8%  81.7%       GGACCCTTGGGTGATCGACGACCCCCAGCCGCTCTTCAGCCAGGAGTACGAAAACGAAACGCTGCCGTACCTGCTGGAGG
10 Orf_virus_IHUMI-1                            78.5%  70.9%       GGACCCTTGGGTGATCGACGACCCCCAGCCGCTCTTCAGCCAGGAGTACGAAAACGAAACGCTGCCGTACCTGCTGGAGG
11 Orf_virus_SJ1                                77.2%  74.2%       --------------------------------------------------------------------------------
12 Orf_virus_NP                                 24.1%  23.5%       --------------------------------------------------------------------------------
   consensus/100%                                                   ................................................................................
   consensus/90%                                                    ................................................................................
   consensus/80%                                                    GGACCCsTGGuTGsTCuACGACCCCCAGCCGCTCTTCAGCCAGGAGsACGAAAACGAAACGCTsCCGTACCTGCTGGAGG
   consensus/70%                                                    GGACCCsTGGGTGsTCGACGACCCCCAGCCGCTCTTCAGCCAGGAGTACGAAAACGAAACGCTGCCGTACCTGCTGGAGG

                                                      cov    pid 3121         .         .         :         .         .         .         .         2 3200
 1 Orf_virus_OV-SA00_NC_005336.1_115015-122053 100.0% 100.0%       TTCTGGAGCTAGCGAGGCTGTACATTCGCGTGGGCTGCACGGTGCCCGGAGAGCAGCCCTTTGAGGTGATCACGGGGATC
 2 Orf_virus_D1701                              98.3%  88.3%       TTCTAGAGCTAGCGAGGCTGTACATTCGCGTGGGCTGCACGGTGCCTGGAGAGCAGCCCTTTGAGGTAATCCCGGGGACC
 3 Orf_virus_NA1-11                             97.9%  87.5%       TTCTGGAGCTAGCGAGGCTGTACATTCGCGTGGGCTGCACGGTGCCCGGAGAGCAGCCCTTTGAGGTGATCCCGGGGATC
 4 Orf_virus_OV-HN3_12                          98.1%  87.2%       TTCTGGAGCTAGCGAGGCTGAACATTCGCGTGGGCTGCACGGTGCCCGGAGAGCAGCCCTTTGAGGTGATCCCGGGGATC
 5 Orf_virus_OV-IA82                            98.6%  88.5%       TTCTGGAGCTAGCGAGGCTGTACATTCGCGTGGGCTGCACGGTGCCCGGAGAGCAGCCCTTTGAGGTGATCCCGGGGATC
 6 Orf_virus_NZ2                                98.5%  88.2%       TTCTGGAGCTAGCGAGGCTGTACATTCGCGTGGGCTGCACGGTGCCCGGAGAGCAGCCCTTCGAGGTGATCCCGGGGATC
 7 Orf_virus_YX                                 98.1%  95.5%       TTCTGGAGCTAGCGAGGCTGTACATTCGCGTGGGCTGCACGGTGCCCGGAGAGCAGCCCTTTGAGGTGATCACGGGGATC
 8 Orf_virus_GO                                 98.8%  96.4%       TTCTGGAGCTAGCGAGGCTGTACATTCGCGTGGGCTGCACGGTGCCCGGAGAGCAGCCCTTTGAGGTGATCACGGGGATC
 9 Orf_virus_B029                               91.8%  81.7%       TTCTGGAGCTAGCGAGGCTGTACATTCGCGTGGGCTGCACGGTGCCCGGAGAGCAGCCCTTCGAGGTGATCCCGGGGATC
10 Orf_virus_IHUMI-1                            78.5%  70.9%       TTCTGGAGCTAGCGAGGCTGTACATTCGCGTGGGCTGCACGGTGCCCGGAGAGCAGCCCTTCGAGGTGATCCCGGGGATC
11 Orf_virus_SJ1                                77.2%  74.2%       --------------------------------------------------------------------------------
12 Orf_virus_NP                                 24.1%  23.5%       --------------------------------------------------------------------------------
   consensus/100%                                                   ................................................................................
   consensus/90%                                                    ................................................................................
   consensus/80%                                                    TTCTuGAGCTAGCGAGGCTGsACATTCGCGTGGGCTGCACGGTGCCsGGAGAGCAGCCCTTsGAGGTuATCsCGGGGAsC
   consensus/70%                                                    TTCTGGAGCTAGCGAGGCTGTACATTCGCGTGGGCTGCACGGTGCCCGGAGAGCAGCCCTTsGAGGTGATCsCGGGGATC

                                                      cov    pid 3201         .         .         .         .         :         .         .         . 3280
 1 Orf_virus_OV-SA00_NC_005336.1_115015-122053 100.0% 100.0%       GACTACCCTCACACCAGCATGGAGTTTCTCCAGCACGTTCTACGGCCAAACCGCCGGTTCGCTCCAGCGAAGCTGCACAT
 2 Orf_virus_D1701                              98.3%  88.3%       GACTACCCTCACACCGGCATGGAGTTTCTCCAGCACGTTCTACGGCCAAACCGCCGGTTCGCTCCAGCGAAGCTGCACAT
 3 Orf_virus_NA1-11                             97.9%  87.5%       GACTACCCTCACACCGGCATGGAGTTTCTCCAGCACGTTCTACGGCCAAACCGCCGGTTTGCTCCAGCGAAGCTGCACAT
 4 Orf_virus_OV-HN3_12                          98.1%  87.2%       GACTACCCTCACACTGGCATGGAGTTTCTCCAGCACGTTCTACGGCCAAACCGCCGGTTTGCTCCAGCGAAGCTGCACAT
 5 Orf_virus_OV-IA82                            98.6%  88.5%       GACTACCCCCACACCGGCATGGAGTTTCTCCAGCACGTTCTACGGCCGAACCGCCGGTTCGCTCCGGCGAAGCTGCACAT
 6 Orf_virus_NZ2                                98.5%  88.2%       GACTACCCGCACACCGGCATGGAGTTTCTCCAGCACGTTCTACGGCCGAACCGCCGGTTCGCTCCGGCGAAGCTGCACAT
 7 Orf_virus_YX                                 98.1%  95.5%       GACTACCCTCACACCAGCATGGAGTTTCTCCAGCACGTTCTACGGCCAAACCGCCGGTTCGCTCCAGCGAAGCTGCACAT
 8 Orf_virus_GO                                 98.8%  96.4%       GACTACCCTCACACCAGCATGGAGTTTCTCCAGCACGTTCTACGGCCAAACCGCCGGTTCGCTCCAGCGAAGCTGCACAT
 9 Orf_virus_B029                               91.8%  81.7%       GACTACCCGCACACTGGCATGGAGTTTCTACAGCACGTTCTACGGCCGAACCGCCGGTTCGCTCCGGCGAAGCTGCACAT
10 Orf_virus_IHUMI-1                            78.5%  70.9%       GACTACCCGCACACCGGCATGGAGTTTCTCCAGCACGTTCTACGGCCGAACCGCCGGTTCGCTCCGGCGAAGCTGCACAT
11 Orf_virus_SJ1                                77.2%  74.2%       --------------------------------------------------------------------------------
12 Orf_virus_NP                                 24.1%  23.5%       --------------------------------------------------------------------------------
   consensus/100%                                                   ................................................................................
   consensus/90%                                                    ................................................................................
   consensus/80%                                                    GACTACCCsCACACsuGCATGGAGTTTCTsCAGCACGTTCTACGGCCuAACCGCCGGTTsGCTCCuGCGAAGCTGCACAT
   consensus/70%                                                    GACTACCCsCACACsuGCATGGAGTTTCTCCAGCACGTTCTACGGCCuAACCGCCGGTTsGCTCCuGCGAAGCTGCACAT

                                                      cov    pid 3281         .         3         .         .         .         .         :         . 3360
```

```
 1 Orf_virus_OV-SA00_NC_005336.1_115015-122053 100.0% 100.0%      GGACCTCGAAGTGGACTACCGGTGCGTGAGCGCCGTTTACGTGAAGGCGTTCCTGCAGGACGCCTGTAGCGCCCGCAAGG
 2 Orf_virus_D1701                              98.3%  88.3%      GGACCTCGAGGTGGACTACCGGTGCGTGAGCGCCGTCCACGTGAAGGCGTTCCTGCAGGACGCCTGTAGCGCCCGCAAGG
 3 Orf_virus_NA1-11                             97.9%  87.5%      GGACCTCGAGGTGGACCACCGGTGCGTGATCGCCGTCCACGTGAAGGCGTTCCTGCAGGACGCCTGTAGCGCCCGCAAGG
 4 Orf_virus_OV-HN3_12                          98.1%  87.2%      GGACCTCGAGGTGGACCACCGGTGCGTGATCGCCGTCCACGTGAAGGCGTTCCTGCAGGACGCCTGTAGCGCCCGCAAGG
 5 Orf_virus_OV-IA82                            98.6%  88.5%      GGACCTCGAGGTGGACCACCGGTGCGTGAGCGCCGTCCACGTGAAGGCGTTCCTGCAGGACGCCTGTAGCGCCCGCAAGG
 6 Orf_virus_NZ2                                98.5%  88.2%      GGACCTCGAGGTGGACCACCGGTGCGTGAGCGCCGTCCACGTGAAGGCGTTCCTGCAGGACGCCTGTAGCGCCCGCAAGG
 7 Orf_virus_YX                                 98.1%  95.5%      GGACCTCGAGGTGGACTACCGGTGCGTGAGCGCCGTCCACGTGAAGGCGTTCCTGCAGGACGCCTGTAGCGCCCGCAAGG
 8 Orf_virus_GO                                 98.8%  96.4%      GGACCTCGAGGTGGACCACCGGTGCGTGAGCGCCGTCCACGTGAAGGCGTTCCTGCAGGACGCCTGTAGCGCCCGCAAGG
 9 Orf_virus_B029                               91.8%  81.7%      GGACCTCGAGGTGGACCACCGGTGCGTGAGCGCCGTCCACGTGAAGGCGTTCCTGCAGGACGCCTGTAGCGCCCGCAAGG
10 Orf_virus_IHUMI-1                            78.5%  70.9%      GGACCTCGAGGTGGACCACCGGTGCGTGAGCGCCGTCCACGTGAAGGCGTTCCTGCAGGACGCCTGTAGCGCCCGCAAGG
11 Orf_virus_SJ1                                77.2%  74.2%      --------------------------------------------------------------------------------
12 Orf_virus_NP                                 24.1%  23.5%      --------------------------------------------------------------------------------
   consensus/100%                                                ................................................................................
   consensus/90%                                                 ................................................................................
   consensus/80%                                                 GGACCTCGAuGTGGACsACCGGTGCGTGAsCGCCGTssACGTGAAGGCGTTCCTGCAGGACGCCTGTAGCGCCCGCAAGG
   consensus/70%                                                 GGACCTCGAGGTGGACsACCGGTGCGTGAsCGCCGTCCACGTGAAGGCGTTCCTGCAGGACGCCTGTAGCGCCCGCAAGG

                                                  cov    pid 3361        .         .         .         4         .         .         .         . 3440
 1 Orf_virus_OV-SA00_NC_005336.1_115015-122053 100.0% 100.0%      CGCGGACGCCGCTCTACTTCGCGGGGCATGGCTCCAATCATCCAGATCGCCGGCC-AAAAAACCCAGTACCGCGCCCTCA
 2 Orf_virus_D1701                              98.3%  88.3%      CGCGGACGCCACTCTACTTTGCGGGGCATGGCTCCAACCATCCAGATCGCCGGCCAAAAAAACCCAGTACCGCGCCCTCA
 3 Orf_virus_NA1-11                             97.9%  87.5%      CGCGGACGCCACTCTACTTCGCGGGGCATGGCTGCAACCATCCAGATCGCCGGCC-AAAAAACCCAGTACCGCGCCCTCA
 4 Orf_virus_OV-HN3_12                          98.1%  87.2%      CGCGGACGCCACTCTACTTCGCGGGGCATGGCTGCAACCATCCAGATCGCCGGCC-AAAAAACCCAGTACCGCGCCCTCA
 5 Orf_virus_OV-IA82                            98.6%  88.5%      CGCGGACGCCACTCTACTTCGCGGGGCATGGCTGCAACCATCCAGATCGCCGGCC-AAAAAACCCAGTACCGCGCCCTCA
 6 Orf_virus_NZ2                                98.5%  88.2%      CGCGGACGCCACTCTACTTCGCGGGGCATGGCTGCAACCATCCAGATCGCCGGCC-AAAAAACCCAGTACCGCGCCCTCA
 7 Orf_virus_YX                                 98.1%  95.5%      CGCGGACGCCGCTCTACTTCGCGGGGCATGGCTCCAACCATCCAGATCGCCGGCC-AAAAAACCCAGTACCGCGCCCTCA
 8 Orf_virus_GO                                 98.8%  96.4%      CGCGGACGCCGCTCTACTTCGCGGGGCATGGCTCCAACCATCCAGATCGCCGGCC-AAAAAACCCAGTACCGCGCCCTCA
 9 Orf_virus_B029                               91.8%  81.7%      CGCGGACGCCACTCTACTTCGCGGGGCATGGCTGCAACCATCCAGATCGCCGGCC-AAAAAACCCAGTACCGCGCCCTCA
10 Orf_virus_IHUMI-1                            78.5%  70.9%      CGCGGACGCCACTCTACTTCGCGGGGCATGGCTGCAACCATCCAGATCGCCGGCC-AAAAAACCCAGTACCGCGCCCTCA
11 Orf_virus_SJ1                                77.2%  74.2%      --------------------------------------------------------------------------------
12 Orf_virus_NP                                 24.1%  23.5%      --------------------------------------------------------------------------------
   consensus/100%                                                ................................................................................
   consensus/90%                                                 ................................................................................
   consensus/80%                                                 CGCGGACGCCuCTCTACTTsGCGGGGCATGGCTsCAAsCATCCAGATCGCCGGCC.AAAAAACCCAGTACCGCGCCCTCA
   consensus/70%                                                 CGCGGACGCCuCTCTACTTCGCGGGGCATGGCTsCAACCATCCAGATCGCCGGCC.AAAAAACCCAGTACCGCGCCCTCA

                                                  cov    pid 3441        :         .         .         .         .         5         .         . 3520
 1 Orf_virus_OV-SA00_NC_005336.1_115015-122053 100.0% 100.0%      GCATGTATCGTCGCCGATGTCCAGGAAGTGCCTCATGCAGACGGCGCGCTGAGGGCGCTCACCGCGCTGACGGCGGCCGT
 2 Orf_virus_D1701                              98.3%  88.3%      GCATGTGTCGTCGCCGATGTCCAGGAAGTGCTGCATGCAGACAGCGCGCTGAGGGCGCTCACCGCGCTGACAGCGGTCGT
 3 Orf_virus_NA1-11                             97.9%  87.5%      GCATGTATCGTCGCCGATCTCCAGGAAGTGCAGCATGCAGACGGCGCGCTGAGGGCGCTCACCGCGCTGACGGCAGCCGT
 4 Orf_virus_OV-HN3_12                          98.1%  87.2%      GCATGTATCGTCGCCGATCTCCAGGAAGTGCAGCATGCAGACGGCGCGCTGAGGGCGCTCACCGCGCTGACGGCAGCCGT
 5 Orf_virus_OV-IA82                            98.6%  88.5%      GCACGTGTCGTCACCGATCTCCAGGAAGTGCAGCATGCAGACGGCGCGCTGAGGGCGCTCACCGCGCTGACGGCGGCCGT
 6 Orf_virus_NZ2                                98.5%  88.2%      GCACGTATCGTCGCCGATCTCCAGGAAGTGCAGCATGCAGACGGCGCGCTAAGGGCGCTCACCGCGCTGACGGCGGCCGT
 7 Orf_virus_YX                                 98.1%  95.5%      GCATGTATCGTCACCGATGTCCAGGAAGTGCCTCATGCAGACGGCGCGCTGAGGGCGCTCACCGCGCTGACGGCGGCCGT
 8 Orf_virus_GO                                 98.8%  96.4%      GCATGTATCGTCGCCGATGTCCAGGAAGTGCCTCATGCAGACGGCGCGCTGAGGGCGCTCACCGCGCTGACGGCGGCCGT
 9 Orf_virus_B029                               91.8%  81.7%      GCACGTATCGTCGCCGATCTCCAGGAAGTGCAGCATGCAGACGGCGCGCTGAGGGCGCTCACCGCGCTGGCGGCGGCCGT
10 Orf_virus_IHUMI-1                            78.5%  70.9%      GCACGTATCGTCGCCGATCTCCAGGAAGTGCAGCATGCAGACGGCGCGCTGAGGGCGCTCACCGCGCTGGCGGCGGCCGT
11 Orf_virus_SJ1                                77.2%  74.2%      --------------------------------------------------------------------------------
12 Orf_virus_NP                                 24.1%  23.5%      --------------------------------------------------------------------------------
   consensus/100%                                                ................................................................................
   consensus/90%                                                 ................................................................................
   consensus/80%                                                 GCAsGTuTCGTCuCCGATsTCCAGGAAGTGCssCATGCAGACuGCGCGCTuAGGGCGCTCACCGCGCTGuCuGCuGsCGT
   consensus/70%                                                 GCAsGTuTCGTCuCCGATsTCCAGGAAGTGCssCATGCAGACGGCGCGCTGAGGGCGCTCACCGCGCTGuCGGCuGCCGT

                                                  cov    pid 3521        .         .         :         .         .         .         .         6 3600
 1 Orf_virus_OV-SA00_NC_005336.1_115015-122053 100.0% 100.0%      GGTGTGCGCGATCGCCGTCGCGCTCGAGCGCGGGGAAGAGGCCGACGCCGTGGACCTTATCCTTATAAAATTTTCAATGA
 2 Orf_virus_D1701                              98.3%  88.3%      GGTGTGCGCAATCGCCATCGCGCTCGAGCGCGAGGCGGAGGCCGACGCCGTGGACCTTATCCTTATAAAATTTTCAATGA
 3 Orf_virus_NA1-11                             97.9%  87.5%      GGTGTGCGCGATCGTCATCACGCTCGAGCGCGGGGCGGAGGCCGACGCCGTAGACCTTATACTTATAAAATTTTCAATGA
 4 Orf_virus_OV-HN3_12                          98.1%  87.2%      GGTGTGCGCGATCGTCATCACGCTTGAGCGCGGGGCGGAGGCCGACGCCGTAGACCTTATACTTATAAAATTTTCAATGA
 5 Orf_virus_OV-IA82                            98.6%  88.5%      GGTGTGCGCGATCGCCGTTGCGCTCGAGCGCGGGGCGGAGGCCGACGCCGTGGACCTTATCCTTATAAAATTTTCAATGA
 6 Orf_virus_NZ2                                98.5%  88.2%      GGTGTGCGCGATCGCCATCGCGCTCGAGCGCGGGGCGGAGGCCGACGCCGTGGACCTTATCCTTATAAAATTTTCAATGA
 7 Orf_virus_YX                                 98.1%  95.5%      GGTGTGCGCGATCGCCGTCGCGCTCGAGCGCGGGGCGGAGGCCGACGCCGTTGACCTTATCCTTATAAAATTTTCAATGA
 8 Orf_virus_GO                                 98.8%  96.4%      GGTGTGCGCGATCGCCGTCGCGCTCGAGCGCGGGGCGGAGGCCGACGCCGTTGACCTTATCCTTATAAAATTTTCAATGA
 9 Orf_virus_B029                               91.8%  81.7%      GGTGTGTGCGATCGCCATCGCGCTCGAGCGCGGGGCGGAGGCCGACGCCGTGGACCTTATCCTTATAAAATTTTCAATGA
10 Orf_virus_IHUMI-1                            78.5%  70.9%      GGTGTGCGCGATCGCCATCGCGCTCGAGCGCGGGGCGGAGGCCGACGCCGTGGACCTTATCCTTATAAAATTTTCAATGA
11 Orf_virus_SJ1                                77.2%  74.2%      --------------------------------------------------------------------------------
12 Orf_virus_NP                                 24.1%  23.5%      --------------------------------------------------------------------------------
   consensus/100%                                                ................................................................................
   consensus/90%                                                 ................................................................................
   consensus/80%                                                 GGTGTGsGCuATCGsCuTsuCGCTsGAGCGCGuGGsuGAGGCCGACGCCGTsGACCTTATsCTTATAAAATTTTCAATGA
   consensus/70%                                                 GGTGTGCGCGATCGsCuTCuCGCTCGAGCGCGGGGCGGAGGCCGACGCCGTsGACCTTATsCTTATAAAATTTTCAATGA

                                                  cov    pid 3601        .         .         .         .         :         .         .         . 3680
 1 Orf_virus_OV-SA00_NC_005336.1_115015-122053 100.0% 100.0%      TATGCTAGTTTTTATGCGACCTTCCTTGGAAAAATCGGAATTCAAAAATGAAATAAAACGGCGTTTTGCACGCATATTAT
 2 Orf_virus_D1701                              98.3%  88.3%      TATGCTAGTTTTTATGCAACCTTCCTTGGAAAATTCGGAATTCAAAAATGAAATAAAACGGCGTTTAGCACGCATATTAT
 3 Orf_virus_NA1-11                             97.9%  87.5%      TATGCTAGTTTTTATGCGACCTTCCTTAGAAAATTCGGAATTCAAAAATGAAATAAAACGGCGTTTAGCACGCATATTAT
 4 Orf_virus_OV-HN3_12                          98.1%  87.2%      TATGCTAGTTTTTATGCGACCTTCCTTAGAAAATTCGGAATTCAAAAATGAAATAAAACGGCGTTTAGCACGCATATTAT
 5 Orf_virus_OV-IA82                            98.6%  88.5%      TATGCTAGTTTTTATGCGACCTTCCTTAGAAAATTCGGAATTCAAAAATGAAATAAAACGGCGTTTAGCACGCATATTAT
 6 Orf_virus_NZ2                                98.5%  88.2%      TATGCTAGTTTTTATGCGACCTTCCTTAGAAAATTCGGAATTCAAAAATGAAATAAAACGGCGTTTAGCACGCATATTAT
 7 Orf_virus_YX                                 98.1%  95.5%      TATGCTAGTTTTTATGCGACCTTCCTTGGAAAAATCGGAATTCAAAAATGAAATAAAACGGCGTTTTGCACGCATATTAT
 8 Orf_virus_GO                                 98.8%  96.4%      TATGCTAGTTTTTATGCGACCTTCCTTGGAAAAATCGGAATTCAAAAATGAAATAAAACGGCGTTTTGCACGCATATTAT
 9 Orf_virus_B029                               91.8%  81.7%      TATGCTAGTTTTTATGCGACCTTCCTTAGAAAATTCGGAATTCAAAAATGAAATAAAACGGCGTTTAGCACGCATATTAT
10 Orf_virus_IHUMI-1                            78.5%  70.9%      TATGCTAGTTTTTATGCGACCTTCCTTAGAAAATTCGGAATTCAAAAATGAAATAAAACGGCGTTTAGCACGCATATTAT
11 Orf_virus_SJ1                                77.2%  74.2%      --------------------------------------------------------------------------------
12 Orf_virus_NP                                 24.1%  23.5%      --------------------------------------------------------------------------------
   consensus/100%                                                ................................................................................
   consensus/90%                                                 ................................................................................
   consensus/80%                                                 TATGCTAGTTTTTATGCuACCTTCCTTuGAAAAsTCGGAATTCAAAAATGAAATAAAACGGCGTTTsGCACGCATATTAT
   consensus/70%                                                 TATGCTAGTTTTTATGCGACCTTCCTTuGAAAAsTCGGAATTCAAAAATGAAATAAAACGGCGTTTsGCACGCATATTAT

                                                  cov    pid 3681        .         7         .         .         .         .         :         . 3760
 1 Orf_virus_OV-SA00_NC_005336.1_115015-122053 100.0% 100.0%      TAATACCGACTACCATAG-CAGGCGTCCGCAGCTGCT--GAGAAAGTCCCTTCTACTGCGGGCTCCATGTCATTTCAACG
 2 Orf_virus_D1701                              98.3%  88.3%      TAATACCGACCACCATAGCCAGGCGTCCGCAGCTGCT--GAGAAAGTCCCTT---CTGCGGGCTCCATGTCATTTCAACG
 3 Orf_virus_NA1-11                             97.9%  87.5%      TAATACCGACTACCATGG-CAGGCGTCCGCAGCTGCCAGAAGAAAGTCCCT--TACTGCGGGCTCCATGTCATTTCAACG
 4 Orf_virus_OV-HN3_12                          98.1%  87.2%      TAATACTGACTACCATGG-CAGGCGTCCGCAGCTGCCAGAAGAAAGTCCCTTCTACTGCGGGCTCCATGTCATTTCAACG
 5 Orf_virus_OV-IA82                            98.6%  88.5%      TAATACCGACCACCATGG-CAGGCGTCCGCAGCTGCCAGAAGAAAGTCCCTTCTACTGCGGGCTCCATGTCATTTCAACG
 6 Orf_virus_NZ2                                98.5%  88.2%      TAATACCGACCACCATGG-CAGGCGTCCGCAGCTGCCAGAAGAAAGTCCCTTCTACTGCGGGCTCCATGTCATTTCAACG
 7 Orf_virus_YX                                 98.1%  95.5%      TAATACCGACTACCATAG-CAGGCGTCCGCAGCTGCT--GAGAAAGTCCCTTCTACTGCGGGCTCCATGTCATTTCAACG
 8 Orf_virus_GO                                 98.8%  96.4%      TAATACCGACTACCATAG-CAGGCGTCCGCAGCTGCT--GAGAAAGTCCCTTCTACTGCGGGCTCCATGTCATTTCAACG
 9 Orf_virus_B029                               91.8%  81.7%      TAATACCGACCACCATGG-CAGGCGTCCGCAGCTGCCAGAAGAAAGTCCCTTCTACTGCGGGCTCCATGTCATTTCAACG
10 Orf_virus_IHUMI-1                            78.5%  70.9%      TAATACCGACCACCATGG-CAGGCGTCCGCAGCTGCCAGAAGAAAGTCCCTTCTACTGCGGGCTCCATGTCATTTCAACG
11 Orf_virus_SJ1                                77.2%  74.2%      --------------------------------------------------------------------------------
12 Orf_virus_NP                                 24.1%  23.5%      --------------------------------------------------------------------------------
   consensus/100%                                                ................................................................................
   consensus/90%                                                 ................................................................................
   consensus/80%                                                 TAATACsGACsACCATuG.CAGGCGTCCGCAGCTGCs..uAGAAAGTCCCT....CTGCGGGCTCCATGTCATTTCAACG
   consensus/70%                                                 TAATACCGACsACCATuG.CAGGCGTCCGCAGCTGCs..uAGAAAGTCCCTT.TACTGCGGGCTCCATGTCATTTCAACG

                                                  cov    pid 3761        .         .         .         8         .         .         .         . 3840
 1 Orf_virus_OV-SA00_NC_005336.1_115015-122053 100.0% 100.0%      GGGCAACCGGAGCATCCGGCCGGCGATGTCCGAGGCGTTGCAGAATGACTTCAGCTACAACCCGCGACCGCCTCCGCCGA
 2 Orf_virus_D1701                              98.3%  88.3%      GGGCAACCGGAGCATCCGGCCGGCGATGTCCGAGGCGTTGCAGAATGATTTCAGCTACAACCCGCGACCGCCTCCGCCGA
 3 Orf_virus_NA1-11                             97.9%  87.5%      GGGCAACCGGAGCATCCGGCCGGCGATGTCCGAGGCGCTGCAGAATGATTTCAGCTACAACCCGCGGCCGCCTCCGCCGA
```

```
 4 Orf_virus_OV-HN3_12                                 98.1%  87.2%     GGGCAACCGGAGCATCCGGCCGGCGATGTCCGAGGCGCTGCAGAATGATTTCAGCTACAACCCGCGGCCGCCTCCGCCGA
 5 Orf_virus_OV-IA82                                   98.6%  88.5%     GGGCAACCGGAGCATCCAGCCTGCGATGTCCGAGGCGTTGCAGAATGATTTCAGCTACAACCCGCGACCGCCTCCGCCGA
 6 Orf_virus_NZ2                                       98.5%  88.2%     GGGCAACCGGAGCATCCAGCCGGCGATGTCCGAGGCGTTGCAGAATGATTTCAGCTACAACCCGCGACCGCCTCCGCCGA
 7 Orf_virus_YX                                        98.1%  95.5%     GGGCAACCGGAGCATCCGGCCGGCGATGTCCGAAGCGTTGCAGAACGACTTCAGCTACAACCCGCGACCGCCTCCGCCGA
 8 Orf_virus_GO                                        98.8%  96.4%     GGGCAACCGGAGCATCCGGCCGGCGATGTCCGAGGCGTTGCAGAACGACTTCAGCTACAACCCGCGACCGCCTCCGCCGA
 9 Orf_virus_B029                                      91.8%  81.7%     GGGCAACCGGAGCATCCAGCCGGCGATGTCCGAGGCGTTGCAGAATGATTTCAGCTANNNNNNNNNNNNNNNNNNNNNNN
10 Orf_virus_IHUMI-1                                   78.5%  70.9%     GGGCAACCGGAGCATCCAGCCGGCGATGTCCGAGGCGTTGCAGAATGATTTCAGCTACAACCCGCGACCGCCTCCGCCGA
11 Orf_virus_SJ1                                       77.2%  74.2%     --------------------------------------------------------------------------------
12 Orf_virus_NP                                        24.1%  23.5%     --------------------------------------------------------------------------------
   consensus/100%                                                       ................................................................................
   consensus/90%                                                        ................................................................................
   consensus/80%                                                        GGGCAACCGGAGCATCCuGCCsGCGATGTCCGAuGCGsTGCAGAAsGAsTTCAGCTAsssssssssssssssssssssss
   consensus/70%                                                        GGGCAACCGGAGCATCCuGCCGGCGATGTCCGAGGCGsTGCAGAAsGAsTTCAGCTACAACCCGCGuCCGCCTCCGCCGA

                                                        cov    pid 3841        :       .       .       .       .       9       .       . 3920
 1 Orf_virus_OV-SA00_NC_005336.1_115015-122053        100.0% 100.0%     GCGCAGAAGAGATTGACTTCTTCTGCGTGGACATGCGCAAAGTACTGATGGAAATTGAGGCCAAGCCCAGCAGCTCCAAG
 2 Orf_virus_D1701                                     98.3%  88.3%     GCGCAGAAGAGATTGACTTCTTCTGCGTGGACATGCGCAAAGTACTGATGGAAATCGAGGCTAAGCCTAGCAGCTCCAAG
 3 Orf_virus_NA1-11                                    97.9%  87.5%     GCGCAGAAGAGATTGACTTCTTCTGCGTGGACATGCGCAAAGTACTGATGGAAATCGAGGCCAAGCCCAACAGCTCCAAG
 4 Orf_virus_OV-HN3_12                                 98.1%  87.2%     GCGCAGAAGAGATTGACTTCTTCTGCGTGGACATGCGCAAAGTACTGATGGAAATTGAGGCCAAGCCCAACAGCTCCAAG
 5 Orf_virus_OV-IA82                                   98.6%  88.5%     GCGCAGAAGAGATTGACTTCTTCTGCGTGGACATGCGCAAAGTACTGATGGAAATTGAGGCCAAGCCCAACAGCTCCAAG
 6 Orf_virus_NZ2                                       98.5%  88.2%     GCGCAGAAGAGATTGACTTCTTCTGCGTGGACATGCGCAAAGTACTGATGGAAATTGAGGCCAAGCCCAGCAGCTCCAAG
 7 Orf_virus_YX                                        98.1%  95.5%     GCGCAGAAGAGATTGACTTCTTCTGCGTGGACATGCGCAAAGTACTGATGGAAATTGAGGCCAAGCCCAACAGCTCCAAG
 8 Orf_virus_GO                                        98.8%  96.4%     GCGCAGAAGAGATTGACTTCTTCTGCGTGGACATGCGCAAAGTACTGATGGAAATTGAGGCCAAGCCCAACAGCTCTAAG
 9 Orf_virus_B029                                      91.8%  81.7%     NNNNNNNNNNNNNNNNNNNNNNNNNNNNNNNNNNNNNNNNNNNNNNNNNNNNNNNNNNNNNNNNNNNNNNNNNNNNNGA-
10 Orf_virus_IHUMI-1                                   78.5%  70.9%     GCGCAGAAGAGATTGACTTCTTCTGCGTGGACATGCGCAAAGTACTGATGGAAATTGAGGCCAAG---------------
11 Orf_virus_SJ1                                       77.2%  74.2%     --------------------------------------------------------------------------------
12 Orf_virus_NP                                        24.1%  23.5%     --------------------------------------------------------------------------------
   consensus/100%                                                       ................................................................................
   consensus/90%                                                        ................................................................................
   consensus/80%                                                        sssssssssssssssssssssssssssssssssssssssssssssssssssssssssssssssss...............
   consensus/70%                                                        GCGCAGAAGAGATTGACTTCTTCTGCGTGGACATGCGCAAAGTACTGATGGAAATsGAGGCsAAGssssssssssssuA.

                                                        cov    pid 3921        .       .       :       .       .       .       .       0 4000
 1 Orf_virus_OV-SA00_NC_005336.1_115015-122053        100.0% 100.0%     TACCCCGATTTCATCCACCCGGTTGACAGCAGCCCGCCGTGCACGCCGGCGCGCAAGCGCAACGGCTCCGGCCGCAAGGC
 2 Orf_virus_D1701                                     98.3%  88.3%     TACCCCGATTTCATCCACCCGGTTGACAGCAGCCCGCCGTGCACGCCGGCGCGCAAGCGCAACGGCTCCGGCCGCAAGGT
 3 Orf_virus_NA1-11                                    97.9%  87.5%     TACCCCGATTTCATCCACCCGGTTGACAGCAGCCCGCCGTGCACGCCGGCGCGCAAGCGCAACGGCTTCGGCCGCAAGGC
 4 Orf_virus_OV-HN3_12                                 98.1%  87.2%     TACCCCGATTTCATCCACCTGGTTGACAGCAGCCTGCCGTGCACGCCGGCGCGCAAGCGCAACGGCTTCGGCCGCAAGGC
 5 Orf_virus_OV-IA82                                   98.6%  88.5%     TACCCCAATTTCATCCACCCGGTTGACAGCAGCCCGCCGTGCACGCCGGCGCGCAAGCGCAACGGCTTCGGCCGCAAGGC
 6 Orf_virus_NZ2                                       98.5%  88.2%     TACCCCGATTTCATCCACCCGGTTGACAGCAGCCCGCCGTGCACGCCGGCGCGCAAGCGCAACGGCTTCGGCCGCAAGGC
 7 Orf_virus_YX                                        98.1%  95.5%     TACCCCGATTTCATCCACCCGGTTGACAGCAGCCCGCCGTGCACGCCGGCGCGCAAGCGCAACGGCTCCGGCCGCAAGGC
 8 Orf_virus_GO                                        98.8%  96.4%     TACCCCGATTTCATCCACCCGGTTGACAGCAGCCCGCCGTGCACGCCGGCGCGCAAGCGCAACGGCTCCGGCCGCAAGGC
 9 Orf_virus_B029                                      91.8%  81.7%     -------------------------------------CGGACGATCCTGTGCG-----GCAAAGACTTCAACATAGTGGC
10 Orf_virus_IHUMI-1                                   78.5%  70.9%     --------------------------------------------------------------------------------
11 Orf_virus_SJ1                                       77.2%  74.2%     --------------------------------------------------------------------------------
12 Orf_virus_NP                                        24.1%  23.5%     --------------------------------------------------------------------------------
   consensus/100%                                                       ................................................................................
   consensus/90%                                                        ................................................................................
   consensus/80%                                                        ................................................................................
   consensus/70%                                                        .....................................CGsuCussCCsGsGCG.....GCAAsGuCTsCuuCsssusGGs

                                                        cov    pid 4001        .       .       .       .       :       .       .       . 4080
 1 Orf_virus_OV-SA00_NC_005336.1_115015-122053        100.0% 100.0%     ACCGAACAAGACCCCGGTGCCGCAGCATGCCAAGCGTGACGGCTACTCCCGCTAATGCAGTCCACACACTTCACAC----
 2 Orf_virus_D1701                                     98.3%  88.3%     ACTGAACAAGACCCCGGTGCCGCAGCAGGCCAAGCGTGACGGCTACTCCCGCTAATGCAGTCCACACACTTCACACACTA
 3 Orf_virus_NA1-11                                    97.9%  87.5%     ACCGATCAAAACCCCGGTGCCGCAACAGGCCAAGCGTGACGGCTACTCCCGTTAATGCAGTCCACACACTTCACAC----
 4 Orf_virus_OV-HN3_12                                 98.1%  87.2%     ACCGATCAAAACCCCGGTGCCGCAACAGGCCAAGCGTGACGGCTACTCCCGTTAATGCAGTCCACACACTTCACAC----
 5 Orf_virus_OV-IA82                                   98.6%  88.5%     ACTGAACAAGACCCCGGTGCCGCAGCAGGCCAAGCGTGACGGCTACTCCCGCTAATGCAGTCCACACACTTCACAC----
 6 Orf_virus_NZ2                                       98.5%  88.2%     ACTGAACAAAACCCAGGTGCCGCAGCAGGCCAAGCGTGACGGCTACTCCCGCTAATGCAGTCCACACACTTCACAC----
 7 Orf_virus_YX                                        98.1%  95.5%     ACCGAACAAGACCCCGGTGCCGCAGCAGGCCAAGCGCGACGGCTACTCCCGCTAATGCAGTCCACACACTTCACAC----
 8 Orf_virus_GO                                        98.8%  96.4%     ACCGAACAAGACCCCGGTGCCGCAGCAGGCCAAGCGCGACGGCTACTCCCGCTAATGCAGTCCACACACTTC--------
 9 Orf_virus_B029                                      91.8%  81.7%     GCCG----------------------------------------------------------------------------
10 Orf_virus_IHUMI-1                                   78.5%  70.9%     --------------------------------------------------------------------------------
11 Orf_virus_SJ1                                       77.2%  74.2%     --------------------------------------------------------------------------------
12 Orf_virus_NP                                        24.1%  23.5%     --------------------------------------------------------------------------------
   consensus/100%                                                       ................................................................................
   consensus/90%                                                        ................................................................................
   consensus/80%                                                        ................................................................................
   consensus/70%                                                        uCsG............................................................................

                                                        cov    pid 4081        .       1       .       .       .       .       :       . 4160
 1 Orf_virus_OV-SA00_NC_005336.1_115015-122053        100.0% 100.0%     ACCACA-CAGCACTCAAGCCCACGATCATCACACAATGAAGCAGCCGCAGCCCACAGCCCGCGCTGAGCACGCACATAAA
 2 Orf_virus_D1701                                     98.3%  88.3%     ACTACATCAGCATTCAAGCTCACAATCATCGCACATGAAGCTAGCCAA----------CAGTGCCAAGCACGCACATAAA
 3 Orf_virus_NA1-11                                    97.9%  87.5%     ACTACATCAGCACTCAAGCTTATAATCACCACACAATGAATCAGCCCA--------CCACGTGCCAAGCACACACATAAA
 4 Orf_virus_OV-HN3_12                                 98.1%  87.2%     ACTACATCAGCACTCAAGCTTATAATTACCACACAATGAATCAGCCCA--------CCACGTGCCAAGCACACACATAAA
 5 Orf_virus_OV-IA82                                   98.6%  88.5%     ACTACATCAGCACTCAAGCTTATAATCACCACACAATGAATTAGCCCA--GCCCACACACGTGCCAAGCA--CACATAAA
 6 Orf_virus_NZ2                                       98.5%  88.2%     ACTACATCAGCACTCAAGCTTATAATCACTACACAATGAATCAGCCCA--------CCACGTGCGAAGCACACACATAAA
 7 Orf_virus_YX                                        98.1%  95.5%     ACCACA-CAGCACTCAAGCTCACGATCATCACACAATGAAGCAGCCGCAGCCCACAGCCCGCGCTGAGCACGCACATAAA
 8 Orf_virus_GO                                        98.8%  96.4%     ACCACA-CAGCACTCAAGCTCACGATCATCACACAATGAAGCAGCCGCAGCCCACAGCCCGCGCTGAGCACGCACATAAA
 9 Orf_virus_B029                                      91.8%  81.7%     --------------------------------------------------------------------------------
10 Orf_virus_IHUMI-1                                   78.5%  70.9%     --------------------------------------------------------------------------------
11 Orf_virus_SJ1                                       77.2%  74.2%     --------------------------------------------------------------------------------
12 Orf_virus_NP                                        24.1%  23.5%     --------------------------------------------------------------------------------
   consensus/100%                                                       ................................................................................
   consensus/90%                                                        ................................................................................
   consensus/80%                                                        ................................................................................
   consensus/70%                                                        ................................................................................

                                                        cov    pid 4161        .       .       .       2       .       .       .       . 4240
 1 Orf_virus_OV-SA00_NC_005336.1_115015-122053        100.0% 100.0%     ATCGCCCACAGTCTTGATCATTCCTAATTGTTCCCAATTACTCCCAATCA-CCCTGCCTTACACACACGCACGTTATCAC
 2 Orf_virus_D1701                                     98.3%  88.3%     ATCACCCCC----------TGTCCCGATCGTTCCCAATTGTTCCCAATC--CCGTGCCTTACACGCACGTTAATCACCCG
 3 Orf_virus_NA1-11                                    97.9%  87.5%     ATCACCCAC---------TTGTCCTGATCGTTCCCAATTACTCCCAATCACCCGTGCCTTACACGCACGTTAATCACCCT
 4 Orf_virus_OV-HN3_12                                 98.1%  87.2%     ATCACCCAC---------TTGTCCTGATCGTTCCCAATTACTCCCAATCACCCGTGCCTTACACGCACGTTAATCACCCT
 5 Orf_virus_OV-IA82                                   98.6%  88.5%     ATCACCCAC---------CTGTCCTGATCGTTCCCAATTACTCCCAATCACCCGTGCTTTACACGCACGTAAATCACCCT
 6 Orf_virus_NZ2                                       98.5%  88.2%     ATCACCCAC---------CTGTCCTGATCGTTCCCAA-TACTCCCAATCA-CCGTGCTTTACACGCACGTTAATCACCCT
 7 Orf_virus_YX                                        98.1%  95.5%     ATCACCCACAGTCTTGATCGTTCCTAATTGTTCCCAATTATTCCCAATCA-CCCTGCCTTACACACACGCACGTTATCAC
 8 Orf_virus_GO                                        98.8%  96.4%     ATCACCCACAGTCTTGATCGTTCCTAATTGTTCCCAATTACTCCCAATCA-CCCTGCCTTACACACACGCACGTTATCAC
 9 Orf_virus_B029                                      91.8%  81.7%     --------------------------------------------------------------------------------
10 Orf_virus_IHUMI-1                                   78.5%  70.9%     --------------------------------------------------------------------------------
11 Orf_virus_SJ1                                       77.2%  74.2%     --------------------------------------------------------------------------------
12 Orf_virus_NP                                        24.1%  23.5%     --------------------------------------------------------------------------------
   consensus/100%                                                       ................................................................................
   consensus/90%                                                        ................................................................................
   consensus/80%                                                        ................................................................................
   consensus/70%                                                        ................................................................................

                                                        cov    pid 4241        :       .       .       .       .       3       .       . 4320
 1 Orf_virus_OV-SA00_NC_005336.1_115015-122053        100.0% 100.0%     CCGCCTTCCT--------------------TCTTCGTCCAATTACACATACA-CCCCGTAATTTTGTACTTTTGTACTTT
 2 Orf_virus_D1701                                     98.3%  88.3%     TTCCTTCCTT--------------------CCTCCCCTTAATTACACATACACCCCGTAATGTTGTACTTTTGTACTTT
 3 Orf_virus_NA1-11                                    97.9%  87.5%     CTCCTTCCTTCATGTCGTTCCTGATCGCTCCTCCTCCTTAATCACACA-----CCCCGTAATTTTGTACTTTTGTACTTT
 4 Orf_virus_OV-HN3_12                                 98.1%  87.2%     CTCCTTCCTTCATGTCGTTCCTGATCGCTCCTCCTCCTTAATCACACA-----CCCCGTAATTTTGTACTTTTGTACTTT
 5 Orf_virus_OV-IA82                                   98.6%  88.5%     CTCCTTCGTT---------CCTGATCGCTCCTCCTCCTTAATCACACATACA-CCCCGTAATTTTGTACTTTTGTACTTT
 6 Orf_virus_NZ2                                       98.5%  88.2%     TTCCTTCCTTCATG-CGTTCCTGATCGTTCCTCCTCCTTAATCACACACACA-CCCCGTAATTTTGTACTTTTGTACTTT
```

```
 7 Orf_virus_YX                                  98.1%  95.5%         CCGCCTTCCT---------------------TCTTCCTCCAATTACACATACA-CCCCGTAATTTGTACTTTTGTACTTT
 8 Orf_virus_GO                                  98.8%  96.4%         CCGCCTTCCT---------------------TCTTCCTCCAATTACACATACA-CCCCGTAATTTGTACTTTTGTACTTT
 9 Orf_virus_B029                                91.8%  81.7%         --------------------------------------------------------------------------------
10 Orf_virus_IHUMI-1                             78.5%  70.9%         --------------------------------------------------------------------------------
11 Orf_virus_SJ1                                 77.2%  74.2%         --------------------------------------------------------------------------------
12 Orf_virus_NP                                  24.1%  23.5%         --------------------------------------------------------------------------------
   consensus/100%                                                     ................................................................................
   consensus/90%                                                      ................................................................................
   consensus/80%                                                      ................................................................................
   consensus/70%                                                      ................................................................................

                                                   cov    pid  4321          .         .         :         .         .         .         .         4 4400
 1 Orf_virus_OV-SA00_NC_005336.1_115015-122053  100.0% 100.0%         AATTTTGTACACTTT-TACACTGA-----------CTTTGTACTTTATTTTTGTACCGAAATTGGACAATAATTAT-TTT
 2 Orf_virus_D1701                               98.3%  88.3%         AATTTTGTACACTTT-TACACTGA-----------CTTTGTACTTTATTTTTGTACCAAAATTGGACGATAGTTAT-TTT
 3 Orf_virus_NA1-11                              97.9%  87.5%         AA-TTTGTACACTTTACACACTGA-----------CTTTGTACTTTATTTTTGTACTGAAATTGGACGAT--TTATCTTT
 4 Orf_virus_OV-HN3_12                           98.1%  87.2%         AA-TTTGTACACTTTACACACTGA-----------CTTTGTACTTTATTTTTGTACTGAAATTGGACGAT--TTATCTTT
 5 Orf_virus_OV-IA82                             98.6%  88.5%         AA-TTTGTACACTTTACACACTGACTTTGTACTGCCTTTGTACTTTATTTTTGTACTGAAATTGGACGATACTTATCTTT
 6 Orf_virus_NZ2                                 98.5%  88.2%         AA-TTTGTACACTTTACACACTGACTTTGTACTGCCTTTGTACTTTATTTTTGTACTGAAATTGGACGATACTTATCTTT
 7 Orf_virus_YX                                  98.1%  95.5%         AATTTTGTACACTTT-TACACTGA-----------CTTTGTACTTTATTTTTGTACCGAAATTGGACAATAATTAT-TTT
 8 Orf_virus_GO                                  98.8%  96.4%         AATTTTGTACACTTT-TACACTGA-----------CTTTGTACTTTATTTTTGTACCGAAATTGGACAATAATTAT-TTT
 9 Orf_virus_B029                                91.8%  81.7%         --------------------------------------------------------------------------------
10 Orf_virus_IHUMI-1                             78.5%  70.9%         --------------------------------------------------------------------------------
11 Orf_virus_SJ1                                 77.2%  74.2%         --------------------------------------------------------------------------------
12 Orf_virus_NP                                  24.1%  23.5%         --------------------------------------------------------------------------------
   consensus/100%                                                     ................................................................................
   consensus/90%                                                      ................................................................................
   consensus/80%                                                      ................................................................................
   consensus/70%                                                      ................................................................................

                                                   cov    pid  4401          .         .         .         .         :         .         .         . 4480
 1 Orf_virus_OV-SA00_NC_005336.1_115015-122053  100.0% 100.0%         GTATCCACATCCAACTTTTGCGAATTCCACACGCCAGTTGCGAAAAATGAAATAGTACCGTTTTAGGCTTCAAT-CCCCC
 2 Orf_virus_D1701                               98.3%  88.3%         GTATTCACATCCAAATTTCGCAAATTCCACACGCCGGTCGCGAAAAATGAAATCGTACCGTTTTAGGCGTCGAT-CCCCC
 3 Orf_virus_NA1-11                              97.9%  87.5%         GTATTCACATACAAAATTTGCAAATTCCA-ACACCGGTTGCGAAAAGTGAAATCGTACCGTTTTAGGCTTCGAT--CCCC
 4 Orf_virus_OV-HN3_12                           98.1%  87.2%         GTATTCACATACAAAATTTGCAAATTTCA-ACACCGGTTGCGAAAAGTGAAATCGTACCGTTTTAGGCTTTGAT--CCCC
 5 Orf_virus_OV-IA82                             98.6%  88.5%         GTATTCACATCCAAGTTTTGCAAATTCCACA-GCCGGTCGCGAAAAGTGAAATCGTACCGTTTTAGGCTTCGAT-CCCCC
 6 Orf_virus_NZ2                                 98.5%  88.2%         GTATTCACATCCAAGTTTTGCAAATTCCACA-GCCGGTAGCGAAAAGTGAAATCGTACCGTTTTAGGCTTCGAT-CCCCC
 7 Orf_virus_YX                                  98.1%  95.5%         GTATCCACATCCAAATTTTGCGAATTCCACACGCCGGTCGCGAAAAATGAAATAGTACCGTTTTAGGCTTTGATCCCCCC
 8 Orf_virus_GO                                  98.8%  96.4%         GTATCCACATCCAA-CTTTGCGAATTCCACACGCCGGTCGCGAAAAATGAAATAGTACCGTTTTAGGCTTCAAT-CCCCC
 9 Orf_virus_B029                                91.8%  81.7%         --------------------------------------------------------------TCGGGGTT----------
10 Orf_virus_IHUMI-1                             78.5%  70.9%         --------------------------------------------------------------------------------
11 Orf_virus_SJ1                                 77.2%  74.2%         --------------------------------------------------------------------------------
12 Orf_virus_NP                                  24.1%  23.5%         --------------------------------------------------------------------------------
   consensus/100%                                                     ................................................................................
   consensus/90%                                                      ................................................................................
   consensus/80%                                                      ................................................................................
   consensus/70%                                                      ..............................................................TsuGGssT..........

                                                   cov    pid  4481          .         5         .         .         .         .         :         . 4560
 1 Orf_virus_OV-SA00_NC_005336.1_115015-122053  100.0% 100.0%         TCCCGCGCGAAGACTCACCAGCATGGACTCTCGTCGGCTCGCCCTTGCCGTCGCCTTCGGAGGCGTCCTCGCCAGCATGA
 2 Orf_virus_D1701                               98.3%  88.3%         TCCTGAGCGAAGACTCGCCAGCATGGACTCTCGTAGGCTCGCTCTCGCCGTTGCCTTCGGAGGCGTCCTCGCCAGCATGA
 3 Orf_virus_NA1-11                              97.9%  87.5%         TCCCGCGCGAAGACTCGCCAGGATGGACTCTCGTCGGCTCGCCCTTGCCGTCGCCTTCGGAGGCGTGCTCGC--------
 4 Orf_virus_OV-HN3_12                           98.1%  87.2%         TCCCGCGCGAAGACTCGCCAGGATGGACTCTCGTCGGCTCGCCCTTGCCGTTGCCTTTGGAGGCGTGCTTGCTAGCATGA
 5 Orf_virus_OV-IA82                             98.6%  88.5%         TCCCGCGCGAAGACTCGCCAGCATGGACTCTCGTAGGCTCGCTCTTGCCGTCGCCTTCGGAGGCGTCCTCGCCAGCATGA
 6 Orf_virus_NZ2                                 98.5%  88.2%         TCCCGCGCGAAGACTCGCCAGCATGGACTCTCGTAGGCTCGCTCTTGCCGTCGCCTTCGGAGGCGTCCTCGCCAGCATGA
 7 Orf_virus_YX                                  98.1%  95.5%         TCCCGCGCGAAGACTCGCCACCATGGACTCTCGTCGGCTCGCCCTTGCTGTTGCCTTCGGAGGCGTCCTCGCCAGCATGA
 8 Orf_virus_GO                                  98.8%  96.4%         TCCCGCGCGAAGACTCGCCAGCATGGACTCTCGTCGGCTCGCCCTTGCCGTTGCCTTTGGAGGCGTCCTCGCCAGCATGA
 9 Orf_virus_B029                                91.8%  81.7%         -------CAAAGACTCGCCAGCATGGACTCTCGTAGGCTCGCTCTTGCCGTCGCCTTCGGAGGCGTCCTCGCCAGCATGG
10 Orf_virus_IHUMI-1                             78.5%  70.9%         --------------------------------------------------------------------------------
11 Orf_virus_SJ1                                 77.2%  74.2%         --------------------------------------------------------------------------------
12 Orf_virus_NP                                  24.1%  23.5%         --------------------------------------------------------------------------------
   consensus/100%                                                     ................................................................................
   consensus/90%                                                      ................................................................................
   consensus/80%                                                      ................................................................................
   consensus/70%                                                      .......CuAAGACTCuCCAssATGGACTCTCGTsGGCTCGCsCTsGCsGTsGCCTTsGGAGGCGTsCTsGC........

                                                   cov    pid  4561          .         .         .         6         .         .         .         . 4640
 1 Orf_virus_OV-SA00_NC_005336.1_115015-122053  100.0% 100.0%         CGCAGCGCCGCCGCCTGGCTTCTCTCATCGCCAGCATCGGCCAACGGCTGATGGGCGGCGACGGCATGCGTCGCGTCGCC
 2 Orf_virus_D1701                               98.3%  88.3%         CACAGCGCCGCCGCCTGGCTTCTCTCATCGCCAGCATCGGCCAACGGCTGATGGGCGGCGACGGCATGCGTCGCGTCGCC
 3 Orf_virus_NA1-11                              97.9%  87.5%         CACAGCGCCGCCGCCTGGCTTCTCTCATCGCCAGCATCGGCCAACGGCTGATGGGCGGCGACGGCATGCGTCGCGTCGCC
 4 Orf_virus_OV-HN3_12                           98.1%  87.2%         CACAGCGCTGCCGCCTGGCTTTTCTTATTGCCAGCATCGGCCAACGGCTGATGGGCGGCGACGGCATGCGTCGCGTCGCC
 5 Orf_virus_OV-IA82                             98.6%  88.5%         CACAGCGCCGCCGCCTGGCTTCTCTCATCGCCAGCATCGGCCAACGGCTGATGGGCGGCGACGGCATGCGTCGCGTCGCC
 6 Orf_virus_NZ2                                 98.5%  88.2%         CACAGCGCCGCCGCCTGGCTTCTCTCATCGCCAGCATCGGCCAACGGCTGATGGGCGGCGACGGCATGCGTCGCGTCGCC
 7 Orf_virus_YX                                  98.1%  95.5%         CGCAGCGCCGCCGCCTGGCCTCTCTCATCGCCAGCATCGGCCAACGGCTGATGGGCGGCGACGGCATGCGTCGCGTCGCC
 8 Orf_virus_GO                                  98.8%  96.4%         CGCAGCGCCGCCGCTTGGCTTCTCTCATCGCCAGCATCGGCCAACGGCTGATGGGCGGCGACGGCATGCGTCGCGTCGCC
 9 Orf_virus_B029                                91.8%  81.7%         CACAGCGCCGCCGCCTGGCTTCTCTCATCGCCAGCATCGGCCAACGGCTGATGGGCGGCGACGGCATGCGTCGCGTCGCT
10 Orf_virus_IHUMI-1                             78.5%  70.9%         --------------------------------------------------------------------------------
11 Orf_virus_SJ1                                 77.2%  74.2%         -----------------------CTCATCGCCAGCATCGGCCAACGGCTGATGGGCGGCGACGGCATGCGTCGCGTCGCC
12 Orf_virus_NP                                  24.1%  23.5%         --------------------------------------------------------------------------------
   consensus/100%                                                     ................................................................................
   consensus/90%                                                      ................................................................................
   consensus/80%                                                      .......................CTsATsGCCAGCATCGGCCAACGGCTGATGGGCGGCGACGGCATGCGTCGCGTCGCs
   consensus/70%                                                      CuCAGCGCsGCCGCsTGGCsTsTCTCATCGCCAGCATCGGCCAACGGCTGATGGGCGGCGACGGCATGCGTCGCGTCGCC

                                                   cov    pid  4641          :         .         .         .         .         7         .         . 4720
 1 Orf_virus_OV-SA00_NC_005336.1_115015-122053  100.0% 100.0%         GTTCGGTTGATCGACCAGCTCATGGCCGGACCTCCGGACATCGACGACGAAGCCTTCCAGCGCGAGATCCGCGTGGGCGT
 2 Orf_virus_D1701                               98.3%  88.3%         GTTCGGTTGATTGACCAGCTCATGGCCGGACCCCCGGACATCGACGACGAGGCCTTCCAGCGCGAGATCC------GCGT
 3 Orf_virus_NA1-11                              97.9%  87.5%         GTTCGGTTGATCGACCAGCTCATGGCCGGACCCCCGGACATCAACGACGAGGCCTTCCAGCGCGAGATCCGCGTGGGCGT
 4 Orf_virus_OV-HN3_12                           98.1%  87.2%         GTTTGGTTGATTGACTAGCTCATGGCCGGACTCCCGGACATCAACGACGAGGCCTTCTAGCGCGAGATCCGCGTGGGCGT
 5 Orf_virus_OV-IA82                             98.6%  88.5%         GTTCGGTTGATCGACCAGCTCATGGCCGGACCCCCGGACATCAACGACGAGGCCTTCCAGCGCGAGATCC------GCGT
 6 Orf_virus_NZ2                                 98.5%  88.2%         GTTCGGTTGATCGACCAGCTCATGGCCGGACCCCCGGACATCAACGACGAGGCCTTCCAGCGCGAGATCC------GCGT
 7 Orf_virus_YX                                  98.1%  95.5%         GTTCGGCTGATCGACCAGCTCATGGCCGGACCCCCGGACATCGACGACGAGGCCTTC-----------------------
 8 Orf_virus_GO                                  98.8%  96.4%         GTTCGGCTGATCGACCAGCTCATGGCCGGACCCCCGGACATCGACGACGAGGCCTTCCAGCGCGAGATCCGCGTGGGCGT
 9 Orf_virus_B029                                91.8%  81.7%         GTTCGGTTGATCGACCAGCTCATGGCCGGACCCCCGGACATCAACGACGAGGCCTTCCAGCGCGAGATCCGCGTGGGCGT
10 Orf_virus_IHUMI-1                             78.5%  70.9%         --------------------------------------------------------------------------------
11 Orf_virus_SJ1                                 77.2%  74.2%         GTTCGGTTGATCGACCAGCTCATGGCCGGACCCCCGGACATCGACGACGAGGCCTTCCAGCGCGAGATCCGCGTGGGCGT
12 Orf_virus_NP                                  24.1%  23.5%         --------------------------------------------------------------------------------
   consensus/100%                                                     ................................................................................
   consensus/90%                                                      ................................................................................
   consensus/80%                                                      GTTsGGsTGATsGACsAGCTCATGGCCGGACssCCGGACATCuACGACGAuGCCTTC.......................
   consensus/70%                                                      GTTCGGsTGATsGACCAGCTCATGGCCGGACCCCCGGACATCuACGACGAGGCCTTCsAGCGCGAGATCC......GCGT

                                                   cov    pid  4721          .         .         :         .         .         .         .         8 4800
 1 Orf_virus_OV-SA00_NC_005336.1_115015-122053  100.0% 100.0%         GGGCGAGCTCTTCCAGGCGCTCCACCGCGTGGTCGAGCAGACACGCCGAGAGAAGTACTTCGAGGTTTGTGGCGCCAGCA
 2 Orf_virus_D1701                               98.3%  88.3%         AGGCGAGCTCTTCCAGGCGCTCCACCGCGTGGTCGAGCAGGCACGCCGAGAGAAGTACTTCGAGGTCTGCGGCGCCGGCA
 3 Orf_virus_NA1-11                              97.9%  87.5%         GGGCGAGCTCTTTCAGGCGCTCCACCGCGTGGTCGAGCAGACACGTCGAGAGAAGTACTTCGAGGTCTGCGGCGCCGGTA
 4 Orf_virus_OV-HN3_12                           98.1%  87.2%         GGGCGAGCTTTTTCAGGCGCTCCACCGCGTGGTCGAGCAGACACGTCGAGAGAAGTACTTTGAGGTTTGCGGCGCCGGTA
 5 Orf_virus_OV-IA82                             98.6%  88.5%         GGGCGAGCTCTTCCAGGCGCTCCACCGCGTGGTCGAGCAGGCACGCCGAGAGAAGTACTTCGAGGTCTGCGGCGCCGGCA
 6 Orf_virus_NZ2                                 98.5%  88.2%         GGGCGAGCTCTTCCAGGCGCTCCACCGCGTGGTCGAGCAGGCACGCCGAGAGAAGTACTTCGAGGTCTGCGGCGCCGGCA
 7 Orf_virus_YX                                  98.1%  95.5%         -------------CAGGCGCTCCACCGCGTGGTCGAGCAGGCACGCCGAGAGAAGTACTTCGAGGTTTGCGGCGCCAGCA
 8 Orf_virus_GO                                  98.8%  96.4%         GGGCGAGCTCTTCCAGGCGCTCCACCGCGTGGTCGAGCAGGCACGCCGAGAGAAGTACTTCGAGGTTTGCGGCGCCAGCA
 9 Orf_virus_B029                                91.8%  81.7%         GGGCGAGCTCTTCCAGGCGCTCCACCGCGTGGTCGAGCAGGCACGCCGAGAGAAGTACTTCGAGGTCTGCGGCGCCGGCA
```

```
10 Orf_virus_IHUMI-1                              78.5%  70.9%      --------------------------------------------------------------------------------
11 Orf_virus_SJ1                                  77.2%  74.2%      GGGCGAGCTCTTCCAGGCGCTCCACCGCGTGGTCGAGCAGGCACGCCGAGAGAAGTACTTCGAGGTTTGCGGCGCCAGCA
12 Orf_virus_NP                                   24.1%  23.5%      --------------------------------------------------------------------------------
   consensus/100%                                                   ................................................................................
   consensus/90%                                                    ................................................................................
   consensus/80%                                                    ...........CAGGCGCTCCACCGCGTGGTCGAGCAGuCACGsCGAGAGAAGTACTTsGAGGTsTGsGGCGCCuGsA
   consensus/70%                                                    uGGCGAGCTsTTsCAGGCGCTCCACCGCGTGGTCGAGCAGuCACGsCGAGAGAAGTACTTCGAGGTsTGCGGCGCCuGsA

                                                    cov    pid 4801      .         .         .         .         :         .         .         . 4880
 1 Orf_virus_OV-SA00_NC_005336.1_115015-122053 100.0% 100.0%      ACGACGCCGACGCACCCGTCAGCGAGATGGACACCGCGGCCGCACTCCCGCAGCCCCAGCCTGCGCCCCTGGCGATCACG
 2 Orf_virus_D1701                                98.3%  88.3%      ACGGCGCCGACG---CCGTCGTCGAGATGGACACCGCGGCCGCACCCCCGCAGCCCCAGCCTGCGCCCTTTGTGATCACG
 3 Orf_virus_NA1-11                               97.9%  87.5%      ACGGCGCCGACGCGCCCGTCGTCGAGATGGACACCGCGGCCGCACCCCCGCAGCCCCAGCCCGCGCCCTTCGTTGTCACG
 4 Orf_virus_OV-HN3_12                            98.1%  87.2%      ACGGCGCCGACGCGCCCGTCGTCGAGATGGACACCGCGGCCGCACCCCCGCAGCCCCAGCCCGCGCCCTTCGTTGTCACG
 5 Orf_virus_OV-IA82                              98.6%  88.5%      ACGACGCCGACGCGCCCGTCGTCGAGATGGACACCGCGGCCGCACCCCCGCAGCCCCAGCCCGCGCCCTTCGTGGTCACG
 6 Orf_virus_NZ2                                  98.5%  88.2%      ACGACGCCGACGCGCCCGTCGTCGAGATGGACACCGCGGCCGCACCCCCGCAGCCCCAGCCCGCGCCCCTCGTGGTCACG
 7 Orf_virus_YX                                   98.1%  95.5%      ACGACGCCGACGCACCCGTCGTCGAGATGGACACCGCGGCCGCACCCCCGCAGCCCCAGCCCGCGCCCCTGGCGATCACG
 8 Orf_virus_GO                                   98.8%  96.4%      ACGACGCCGACGCACCCGTCGTCGAGATGGACACCGCGGCCGCACCCCCGCAGCCCCAGCCCGCGCCCCTCGCGATTACG
 9 Orf_virus_B029                                 91.8%  81.7%      ACGACGCCGACGCGCCCGTCGTCGAGATGGACACTGCGGCCGCACCCCCGCAGCCCCAGCCCGCGCCCTTCGTGGTCACG
10 Orf_virus_IHUMI-1                              78.5%  70.9%      --------------------------------------------------------------------------------
11 Orf_virus_SJ1                                  77.2%  74.2%      ACGACGCCGACGCACCCGTCGGCGAGATGGACACCGCGGCCGCACCCCCGCAGCCCCAGCCCGCGCCCCTGGCGATTACG
12 Orf_virus_NP                                   24.1%  23.5%      --------------------------------------------------------------------------------
   consensus/100%                                                   ................................................................................
   consensus/90%                                                    ................................................................................
   consensus/80%                                                    ACGuCGCCGACG...CCGTCusCGAGATGGACACsGCGGCCGCACsCCCGCAGCCCCAGCCsGCGCCCsTsGssuTsACG
   consensus/70%                                                    ACGuCGCCGACGCuCCCGTCGsCGAGATGGACACCGCGGCCGCACCCCCGCAGCCCCAGCCsGCGCCCsTsGssuTsACG

                                                    cov    pid 4881      .         9         .         .         .         .         :         . 4960
 1 Orf_virus_OV-SA00_NC_005336.1_115015-122053 100.0% 100.0%      CCACAGAACGCGTTCATGTTCGTGCCGCAAAGCAGCCACGTGCACGTGGACGAGAGCGTGGACCCGTTCTTCGGCATGAG
 2 Orf_virus_D1701                                98.3%  88.3%      CCGCAGAACGCGTTCATGTTCGTGCCGCAAAACAGCCACGTGCATGTGGACGAGAGCGTGGACCCGTTCTTCGGCATGAG
 3 Orf_virus_NA1-11                               97.9%  87.5%      CCGCAGAACGCGTTCGTGTTCGTGCCGCAAAGCAGCCACGTGCACGTGGACGAGAGCGTGGACCCGTTCTTCGGCATGAG
 4 Orf_virus_OV-HN3_12                            98.1%  87.2%      CCGCAGAACGCGTTCGTGTTTGTGCCGCAAAGCAGCCACGTGCACGTGGACGAGAGCGTGGACCCGTTCTTCGGCATGAG
 5 Orf_virus_OV-IA82                              98.6%  88.5%      CCGCAGAACGCGTTCATGTTCGTGCCGCAAGGCAGCCACGTGCACGTGGACGAGAGCGTGGACCCGTTCTTCGGCATGAG
 6 Orf_virus_NZ2                                  98.5%  88.2%      CCGCAGAACGCGTTCATGTTCGTGCCGCAAGGCAGCCACGTGCACGTGGACGAGAGCGTGGACCCGTTCTTCGGCATGAG
 7 Orf_virus_YX                                   98.1%  95.5%      CCACAGAACGCGTTCATGTTCGTGCCGCAAAGCAGCCACGTGCACGTGGACGAGAGCGTGGACCCGTTCTTCGGCATGAG
 8 Orf_virus_GO                                   98.8%  96.4%      CCGCAGAACGCGTTCATGTTCGTGCCGCAAAGCAGCCACGTGCACGTGGACGAGAGCGTGGACCCGTTCTTCGGCATGAG
 9 Orf_virus_B029                                 91.8%  81.7%      CCGCAGAACGCGTTCATGTTCGTGCCGCAAGGCAGCCACGTGCACGTGGACGAGAGCGTGGACCCGTTCTTCGGCATGAG
10 Orf_virus_IHUMI-1                              78.5%  70.9%      --------------------------------------------------------------------------------
11 Orf_virus_SJ1                                  77.2%  74.2%      CCACAGAACGCGTTCATGTTCGTGCCGCAAAGCAGCCACGTGCACGTGGACGAGAGCGTGGACCCGTTCTTCGGCATGAG
12 Orf_virus_NP                                   24.1%  23.5%      --------------------------------------------------------------------------------
   consensus/100%                                                   ................................................................................
   consensus/90%                                                    ................................................................................
   consensus/80%                                                    CCuCAGAACGCGTTCuTGTTsGTGCCGCAAuuCAGCCACGTGCAsGTGGACGAGAGCGTGGACCCGTTCTTCGGCATGAG
   consensus/70%                                                    CCuCAGAACGCGTTCuTGTTCGTGCCGCAAuGCAGCCACGTGCACGTGGACGAGAGCGTGGACCCGTTCTTCGGCATGAG

                                                    cov    pid 4961      .         .         .         0         .         .         .         . 5040
 1 Orf_virus_OV-SA00_NC_005336.1_115015-122053 100.0% 100.0%      CCCCTCCATCTTCGGGCGCGACATCCCCCTTCAGCCGCCCGAGGAGCTGCTGAGCGACTACGACCCGCTCATGAGCCAGG
 2 Orf_virus_D1701                                98.3%  88.3%      CCCCTCCATCTTCGGGCGCGACCTCCCCCCTCAGCCGCCCGAGGAGCTGCTGAGCGACTACGACCCGCTCATGAGCCAGG
 3 Orf_virus_NA1-11                               97.9%  87.5%      CCCCTCCATCTTCGGGCGCGACCTCCCCTTTCAGCCGCCCGAAGAGCTGCTGAGCGACTACGACCCGCTCATGAGCCAGG
 4 Orf_virus_OV-HN3_12                            98.1%  87.2%      CCCCTCCATCTTTGGGCGCGACCTCCCCTTTTAGCCGCCCGAAGAGCTGCTGAGCGACTACGACCCGCTCATGAGCCAGG
 5 Orf_virus_OV-IA82                              98.6%  88.5%      CCCCTCCATCTTCGGGCGCGACCTCCCCCTTCAGCCGCCCGAGGAGCTGCTGAGCGACCACGACCCGCTCATGAGCCAGG
 6 Orf_virus_NZ2                                  98.5%  88.2%      CCCCTCCATCTTCGGGCGCGACCTCCCCCCTCAGCCGCCCGAGGAGCTGCTGAGCGACTACGACCCGCTCATGAGCCAGG
 7 Orf_virus_YX                                   98.1%  95.5%      CCCCTCCATCTTCGGGCGCGACATCCCCCTTCAGCCGCCAGAGGAGCTGCTGAGCGACTACGACCCGCTCATGAGCCAGG
 8 Orf_virus_GO                                   98.8%  96.4%      CCCCTCCATCTTCGGGCGCGACATCCCCCTTCAGCCGCCAGAGGAGCTGCTGAGCGACTACGACCCGCTCATGAGCCAGG
 9 Orf_virus_B029                                 91.8%  81.7%      CCCCTCCATCTTCGGGCGCGACCTCCCCCTTCAGCCGCCCGAGGAGCTGCTGAGCGACTACGACCCGCTCATGAGCCAGG
10 Orf_virus_IHUMI-1                              78.5%  70.9%      --------------------------------------------------------------------------------
11 Orf_virus_SJ1                                  77.2%  74.2%      CCCCTCCATCTTCGGGCGCGACATCCCCCTTCAGCCGCCCGAGGAGCTGCTGAGCGACTACGACCCGCTCATGAGCCAGG
12 Orf_virus_NP                                   24.1%  23.5%      --------------------------------------------------------------------------------
   consensus/100%                                                   ................................................................................
   consensus/90%                                                    ................................................................................
   consensus/80%                                                    CCCCTCCATCTTsGGGCGCGACsTCCCCssTsAGCCGCCsGAuGAGCTGCTGAGCGACsACGACCCGCTCATGAGCCAGG
   consensus/70%                                                    CCCCTCCATCTTCGGGCGCGACsTCCCCssTCAGCCGCCsGAuGAGCTGCTGAGCGACTACGACCCGCTCATGAGCCAGG

                                                    cov    pid 5041      :         .         .         .         .         .         1         . 5120
 1 Orf_virus_OV-SA00_NC_005336.1_115015-122053 100.0% 100.0%      CCGGCGAGCCGCCGAGCCCGCGGTCGCCCTGCGAGGCCGACCTCTGGTGCTTCGAGACGCTCAACGACAGCGACAGCGAT
 2 Orf_virus_D1701                                98.3%  88.3%      CCGGCGAGCCGCCGAGCCCGCGGTCTCCCTGCGAGGGCGACCTCTGGTGCTTCGAGACGCTCGGCGACAGCGACAGCGAT
 3 Orf_virus_NA1-11                               97.9%  87.5%      CCGGCGAACCGCCGAGTCCGCGGTCGCCCTGCGAGGCAGACCTCTGGTGCTTCGAGACGCTCGGCGACAGCGACAGCGAT
 4 Orf_virus_OV-HN3_12                            98.1%  87.2%      CTGGCGAACTGCCGAGTCCGCGGTCGCCCTGCGAGGCAGACCTTTGGTGCTTTGAGACGCTCGGCGACAGCGACAGCGAT
 5 Orf_virus_OV-IA82                              98.6%  88.5%      CCGGCGAGCCGCCGAGCCCGCGGTCGCCCTGCGAGGCCGACCTCTGGTGCTTCGAGACGCTCGGCGACAGCGACAGCGAT
 6 Orf_virus_NZ2                                  98.5%  88.2%      CCGGCGAGCCGCCGAGCCCGCGGTCGCCCTGCGAGGCCGACCTCTGGTGCTTCGAGACGCTCGGCGACAGCGACAGCGAT
 7 Orf_virus_YX                                   98.1%  95.5%      CCGGCGAGCCGCCGAGCCCGCGGTCGCCCTGCGAGGCCGACCTCTGGTGCTTCGAGACGCTC------AACGACAGCGAT
 8 Orf_virus_GO                                   98.8%  96.4%      CCGGCGAGCCGCCGAGCCCGCGGTCGCCCTGCGAGGCCGACCTCTGGTGCTTCGAGACGCTC------AACGACAGCGAT
 9 Orf_virus_B029                                 91.8%  81.7%      CCGGCGAGCCGCCGAGCCCGCGGTCGCCCTGCGAGGCCGACCTCTGGTGCTTCGAGACGCTCGGCGACAGCGACAGCGAT
10 Orf_virus_IHUMI-1                              78.5%  70.9%      --------------------------------------------------------------------------------
11 Orf_virus_SJ1                                  77.2%  74.2%      CCGGCGAGCCGCCGAGCCCGCGGTCGCCCTGCGAGGCCGACCTCTGGTGCTTCGAGACGCTCAACGACAGCGACAGCGAT
12 Orf_virus_NP                                   24.1%  23.5%      --------------------------------------------------------------------------------
   consensus/100%                                                   ................................................................................
   consensus/90%                                                    ................................................................................
   consensus/80%                                                    CsGGCGAuCsGCCGAGsCCGCGGTCsCCCTGCGAGGssGACCTsTGGTGCTTsGAGACGCTC......AuCGACAGCGAT
   consensus/70%                                                    CCGGCGAuCCGCCGAGsCCGCGGTCGCCCTGCGAGGCsGACCTCTGGTGCTTCGAGACGCTC......AuCGACAGCGAT

                                                    cov    pid 5121      .         .         .         :         .         .         .         2 5200
 1 Orf_virus_OV-SA00_NC_005336.1_115015-122053 100.0% 100.0%      TGAGCCTCGCACCTCACCCCAC---ACCCTTACCTTACCCCACCCACCCAACAC-------------------CTCAAC
 2 Orf_virus_D1701                                98.3%  88.3%      TGAGCC-TGC-CCCCACCCCAC--------------ACTCTACCTCACCTTACCCCAACAC--------------CCCAAC
 3 Orf_virus_NA1-11                               97.9%  87.5%      TGAGCC-CGCACCACACCCCACCTCACC----------------------------------------------ACACCCCAA
 4 Orf_virus_OV-HN3_12                            98.1%  87.2%      TGAGCC-CGCACCACACCCCACCTCACC----------------------------------------------ACACCCCAA
 5 Orf_virus_OV-IA82                              98.6%  88.5%      TGAGCC-CGCACCACACCCCACCTCACCCACCCCACACTCCACCTCACCTCACCCTAACACCAACACCCTAACACCCAAC
 6 Orf_virus_NZ2                                  98.5%  88.2%      TGAGAC-CGCACCACACCCTACCTCACCCACCCCACACTCCACCTCACCTCACCCTAACAC-------CCAACACCCAAC
 7 Orf_virus_YX                                   98.1%  95.5%      TGAGCCTCGCACCTCACACCA----------------------------------------------------------AC
 8 Orf_virus_GO                                   98.8%  96.4%      TGAGCCTCGCACCTCACCCCACCCTACCTCACCCACACCCACCCACA------------------------------CCAAC
 9 Orf_virus_B029                                 91.8%  81.7%      TGAGCC-CGCACCACACCCCAC--------------ACTCCACCTCACCTCACCCTAAC---------CCAACACCCAAC
10 Orf_virus_IHUMI-1                              78.5%  70.9%      --------------------------------------------------------------------------------
11 Orf_virus_SJ1                                  77.2%  74.2%      TGAGCCTCGCACCTCACCCCACCCACCCCACACCTACCTCACCCACACCCACTCACA------------------CCAAC
12 Orf_virus_NP                                   24.1%  23.5%      --------------------------------------------------------------------------------
   consensus/100%                                                   ................................................................................
   consensus/90%                                                    ................................................................................
   consensus/80%                                                    TGAGsC.sGC.CCsCACsCsA..........................................................As
   consensus/70%                                                    TGAGCC.CGCACCsCACCCCAC.......................................................sCsAs

                                                    cov    pid 5201      .         .         .         .         .         :         .         . 5280
 1 Orf_virus_OV-SA00_NC_005336.1_115015-122053 100.0% 100.0%      ACCTCGACCGGACAATGAAGGAGTCCCACATTTCACT---GGACGCGGATGAAGCCGCACATCTCCCACATGAAGGATCG
 2 Orf_virus_D1701                                98.3%  88.3%      ACCCCAGCCGGACAATGAAGGAGTCCCACATTTCACTGAAGGACGCGGATGAAGCCGCACATCCCCCACATGAAGGATTG
 3 Orf_virus_NA1-11                               97.9%  87.5%      ACCTCAACCGGACAATGAAGGAGTCCCACATTTCA---AAGGACGCGGATG-AGCCGCACAT--CCCACATGAAGGATTG
 4 Orf_virus_OV-HN3_12                            98.1%  87.2%      ACCTCAACCGGACAATGAAGGAGTCTCACATTTCA---AAGGACGCGGATG-AGCCGCACAT--CCCACATGAAGGATTG
 5 Orf_virus_OV-IA82                              98.6%  88.5%      ACCTCAACCGGACAATGAAGGAGTCCCACATTTCACTGAAGGACGCGGATGAAGCCGCACAT-CCCCACATGAAGGATTG
 6 Orf_virus_NZ2                                  98.5%  88.2%      ACCTCAACCGGACAATGAAGGAGTCCCACATTTCACTGAAGGACGCGGATGAAGCCGCAC-TCCCCACATGAAGGATTG
 7 Orf_virus_YX                                   98.1%  95.5%      ACCTCAACCGGACAATGAAGGAGTCCCACATTTCA---AAGGACGCGGATGAAGCCGCACATCTCCCACATGAAGGATCG
 8 Orf_virus_GO                                   98.8%  96.4%      ACCTCAACCGGACAATGAAGGAGTCCCACATTTCA---AAGGACGCGGATGAAGCCGCACATCTCCCACATGAAGGATCG
 9 Orf_virus_B029                                 91.8%  81.7%      ACCTCAACCGGACAATGAAGGAGTCCCACATTTCACT-AAGGACGCGGATGAAGCCGCACAT-CCCCACATGAAGGATTG
10 Orf_virus_IHUMI-1                              78.5%  70.9%      --------------------------------------------------------------------------------
11 Orf_virus_SJ1                                  77.2%  74.2%      ACCTCAACCGGACAATGAAGGAGTCCCACATTTCACT---GGACGCGGATGAAGCCGCACATCTCCCACATGAAGGATCG
12 Orf_virus_NP                                   24.1%  23.5%      --------------------------------------------------------------------------------
```

```
    consensus/100%                                                ................................................................................
    consensus/90%                                                 ................................................................................
    consensus/80%                                                 ACCsCuuCCGGACAATGAAGGAGTCsCACATTTCA.....GGACGCGGATG.AGCCGCAC.T..CCCACATGAAGGATsG
    consensus/70%                                                 ACCTCAACCGGACAATGAAGGAGTCCCACATTTCA.....GGACGCGGATG.AGCCGCACAT..CCCACATGAAGGATsG

                                                      cov    pid 5281          .         3         .         .         .         .         :         . 5360
  1 Orf_virus_OV-SA00_NC_005336.1_115015-122053 100.0% 100.0%     GCCAACGGTCAAACATTTCACCTGC--AATGAAGGACGATGCGCGGTCGCGATTGGCCTGCGACCGACATCGCACAC--A
  2 Orf_virus_D1701                               98.3%  88.3%     G-CAACGGTCAAACATTTCACCTGC--AATGAAGGACGATGCGCGGTCGCAATTGGCCTGCGACCGACATCGCACACAAA
  3 Orf_virus_NA1-11                              97.9%  87.5%     G-CAACGGTCAAACATTTCACCTGCAAAATGAAGGACGATGCGCGGTCGCGATTGGCTTGCGACCGACATCGCACACAAA
  4 Orf_virus_OV-HN3_12                           98.1%  87.2%     G-CAACGGTCAAACATTTTACCTGCAAAATGAAGGACGATGCGCGGTCGCGATTGGCTTGCGACCGACATCGCACACAAA
  5 Orf_virus_OV-IA82                             98.6%  88.5%     G-CAACGGTCAAACATTTCACCTGC--AATGAAGGACGATGCGCGGTCGC-ATTGGCCTGCGACCGACATCGCACAC--A
  6 Orf_virus_NZ2                                 98.5%  88.2%     G-CAACGGTCAAACATTTCACCTGC--AATGAAGGACGATGCGCGGTCGC-ATTGGCCTGCGACCGACATCGCACAC--A
  7 Orf_virus_YX                                  98.1%  95.5%     G--CACGGTCAAACATTTCACCTGC--AATGAAGGACGATGCGCGGTCGCGATTGGCCTGCGACCGACATCGCACAC--A
  8 Orf_virus_GO                                  98.8%  96.4%     G-CAACGGTCAAACATTTCACCTGC--AATGAAGGACGATGCGCGGTCGCGATTGGCCTGCGACCGACATCGCACAC--A
  9 Orf_virus_B029                                91.8%  81.7%     G-CAACGGTCAAACATTTCACCTGC--AATGAAGGACGATGCGCGGTCGC-ATTGGCCTGCGACCGACATCGCTCAC--A
 10 Orf_virus_IHUMI-1                             78.5%  70.9%     --------------------------------------------------------------------------------
 11 Orf_virus_SJ1                                 77.2%  74.2%     G-CAACGGTCAAACATTTCACCTGC--AATGAAGGACGATGCGCGGTCGCGATTGGCCTGCGACCGACATCGCACAC--A
 12 Orf_virus_NP                                  24.1%  23.5%     --------------------------------------------------------------------------------
    consensus/100%                                                ................................................................................
    consensus/90%                                                 ................................................................................
    consensus/80%                                                 G..sACGGTCAAACATTTsACCTGC..AATGAAGGACGATGCGCGGTCGC.ATTGGCsTGCGACCGACATCGCsCAC..A
    consensus/70%                                                 G.CAACGGTCAAACATTTCACCTGC..AATGAAGGACGATGCGCGGTCGC.ATTGGCsTGCGACCGACATCGCACAC..A

                                                      cov    pid 5361          .         .         .         4         .         .         .         . 5440
  1 Orf_virus_OV-SA00_NC_005336.1_115015-122053 100.0% 100.0%     TGAAGGACACAATTGGTTTGTTAATCCGGACAATGAAGGACAAATTGTTTTTGTTAATCAGGACAATTGGAACACAATCA
  2 Orf_virus_D1701                               98.3%  88.3%     TGAAGGACACAATTGGTTTGTTAATCCGGACAATGAAGG-CAAATTGTTTTTGTTAATCAGGACAATTGGAACACAATCA
  3 Orf_virus_NA1-11                              97.9%  87.5%     TGAAGGACACAATTTGTTTGTTAATCCGGACAATGAAGGACAAATTGTTTTTGTTAATCAGGACAATTGGAACACAATCA
  4 Orf_virus_OV-HN3_12                           98.1%  87.2%     TGAAGGACACAATTTGTTTGTTAATCTGGACAATGAAGGACAAATTGTTTTTGTTAATCAGGACAATTGGAACACAATTA
  5 Orf_virus_OV-IA82                             98.6%  88.5%     TGAAGGACACAATTGGTTTGTTAATCCGGACAATGAAGGACAAATTGTTTTTGTTAATCAGGACAATTGG-ACACAATCA
  6 Orf_virus_NZ2                                 98.5%  88.2%     TGAAGGACACAATTGGTTTGTTAATCCGGACAATGAAGGACAAATTGTTTTTGTTAATCAGGACAATTAGAACACAATCA
  7 Orf_virus_YX                                  98.1%  95.5%     TGAAGGACACAATTGGTTTGTTAATCCGGACAATGAAGGACAAATTGTTTTTGTTAATCAGGACAATTGGAACACAATCA
  8 Orf_virus_GO                                  98.8%  96.4%     TGAAGGACACAATTGGTTTGTTAATCCGGACAATGAAGGACAAATTGTTTTTGTTAATCAGGACAATTGGAACACAATCA
  9 Orf_virus_B029                                91.8%  81.7%     TGAAGGACACAATTGGTTTGTTAATCCGGACAATGAAGGACAAATTGTTTTTGTTAATCAGGACAATTGGAACACAATCA
 10 Orf_virus_IHUMI-1                             78.5%  70.9%     ------------------------------------------------TTTGTTAATCAGGACAATTAGAACACAATCA
 11 Orf_virus_SJ1                                 77.2%  74.2%     TGAAGGACACAATTGGTTTGTTAATCCGGACAATGAAGGACAAATTGTTTTTGTTAATCAGGACAATTGGAACACAATCA
 12 Orf_virus_NP                                  24.1%  23.5%     --------------------------------------------------------------------------------
    consensus/100%                                                ................................................................................
    consensus/90%                                                 ................................................TTTGTTAATCAGGACAATTuG.ACACAATsA
    consensus/80%                                                 TGAAGGACACAATTsGTTTGTTAATCsGGACAATGAAGG.CAAATTGTTTTTGTTAATCAGGACAATTuGAACACAATCA
    consensus/70%                                                 TGAAGGACACAATTsGTTTGTTAATCCGGACAATGAAGGACAAATTGTTTTTGTTAATCAGGACAATTGGAACACAATCA

                                                      cov    pid 5441          :         .         .         .         .         5         .         . 5520
  1 Orf_virus_OV-SA00_NC_005336.1_115015-122053 100.0% 100.0%     AATTAT-TTTTTGTGCGGTCATAAAATCGATATTTGATGCACATATATTAGTAAGTATATTAGACTAAATTCTCCGGGGA
  2 Orf_virus_D1701                               98.3%  88.3%     GATTAA-TTTTTGTACGATCATAAAATCGATATTTGATGCACATATATTAGTAAGTGTATTAGACTAAATTCTCCGGGGA
  3 Orf_virus_NA1-11                              97.9%  87.5%     GATTAATTTTTTGTACGATCATAAAATCGATATTTGATGCACATATATTAGTAAGTATATTAAACTAAATTCTCCGGGGA
  4 Orf_virus_OV-HN3_12                           98.1%  87.2%     GATTAATTTTTTGTACGATCATAAAATTGATATTTGATGCACATATATTAGTAAGTATATTAAACTAAATTCTCCGGGGA
  5 Orf_virus_OV-IA82                             98.6%  88.5%     GATTAA-TTTTTGTACGATCATAAAATCGATATTTGATGCACATATATTAGTAAGTATATTAGACTAAATTCTCCGGGGA
  6 Orf_virus_NZ2                                 98.5%  88.2%     AA-----TTTTTGTACGATCATAAAATCGATATTTGATGCACATATATTAGTAAGTATATTAGACTAAATTCTCCGGGGA
  7 Orf_virus_YX                                  98.1%  95.5%     AATTAT-TTTTTGTGCGGTCCTAAAATCGATATTTGATGCACATATATTAGTAAGTATATTAGACTAAATTCTCCGGGGA
  8 Orf_virus_GO                                  98.8%  96.4%     AATTAATTTTTTGTGCGGTCCTAAAATCGATATTTGATGCACATATATTAGTAAGTATATTAGACTAAATTCTCCGGGGA
  9 Orf_virus_B029                                91.8%  81.7%     AATTAA-TTTTTGTACGATCATAAAATCGATATTTGATGCACATATATTAGTAAGTATATTAGACTAAATTCTCCGGGGA
 10 Orf_virus_IHUMI-1                             78.5%  70.9%     AATTAA-TTTTTGTACGATCATAAAATCGATATTTGATGCACATATATTAGTAAGTATATTAGACTAAATTCTCCGGGGA
 11 Orf_virus_SJ1                                 77.2%  74.2%     AATTAA-TTTTTGTGCGGTCATAAAATCGATATTTGATGCACATATATTAGTAAGTATATTAGACTAAATTCTCCGGGGA
 12 Orf_virus_NP                                  24.1%  23.5%     --------------------------------------------------------------------------------
    consensus/100%                                                ................................................................................
    consensus/90%                                                 uA.....TTTTTGTuCGuTCsTAAAATsGATATTTGATGCACATATATTAGTAAGTuTATTAuACTAAATTCTCCGGGGA
    consensus/80%                                                 uATTAs.TTTTTGTuCGuTCsTAAAATCGATATTTGATGCACATATATTAGTAAGTATATTAuACTAAATTCTCCGGGGA
    consensus/70%                                                 uATTAs.TTTTTGTuCGuTCATAAAATCGATATTTGATGCACATATATTAGTAAGTATATTAGACTAAATTCTCCGGGGA

                                                      cov    pid 5521          .         .         :         .         .         .         .         6 5600
  1 Orf_virus_OV-SA00_NC_005336.1_115015-122053 100.0% 100.0%     GGCAAGCAGTTGGATACGGCGGGGCGGGGCACGACGTGCACGGAGAATTCGGGCGGG--CCCCCTCCCCCCACCCCCACG
  2 Orf_virus_D1701                               98.3%  88.3%     GGCAAGCAGTTGGATACGGCGGGGCGGGGCACGACGTGCACGGAGAATTCGGGCGGGTCCCCCTTCCCCCCACCCCCACG
  3 Orf_virus_NA1-11                              97.9%  87.5%     GGCAAGCAGTTGGATACGGCGGGGCGGGGCACGACGTGCACGGAGAATTCGGGCGGGTCCCCCTTCCCCCCACCCCCACG
  4 Orf_virus_OV-HN3_12                           98.1%  87.2%     GGCAAGCAGTTGGATACGGCGGGGCGGGGCACGACGTGCACGGAGAATTCGGGCGGGTCCCCCTTCCCCCCACCCCCACG
  5 Orf_virus_OV-IA82                             98.6%  88.5%     GGCAAGCAGTTGGATACGGCGGGGCGGGGCACGACGTGCACGGAGAATTCGGGCGGGTCCCCCTTCCCCCCACCCCCACG
  6 Orf_virus_NZ2                                 98.5%  88.2%     GGCAAGCAGTTGGATACGGCGGGGCGGGGCACGACGTGCACGGAGAGTTCGGGCGGGTCCCCCTTCCCCCCACCCCCACG
  7 Orf_virus_YX                                  98.1%  95.5%     GGCAAGCAGTTGGATACGGCGGGGCGGGGCACGACGTGCACGGAGAATTCGGGCGGG--CCCCCTCCCCCCACCCCCACG
  8 Orf_virus_GO                                  98.8%  96.4%     GGCAAGCAGTTGGATACGGCGGGGCGGGGCACGACGTGCACGGAGAATTCGGGCGGG--CCCCCTCCCCCCACCCCCACG
  9 Orf_virus_B029                                91.8%  81.7%     GGCAAGCAGTTGGATACGGCGGGGCGGGGCACGACGTGCACGGAGAATTCGGGCGGGTCCCCCTTCCCCCCACCCCCACG
 10 Orf_virus_IHUMI-1                             78.5%  70.9%     GGCAAGCAGTTGGATACGGCGGGGCGGGGCACGACGTGCACGGAGAATTCGGGCGGGT-CCCCTTCCCCCCACCCCCACG
 11 Orf_virus_SJ1                                 77.2%  74.2%     GGCAAGCAGTTGGATACGGCGGGGCGGGGCACGACGTGCACGGAGAATTCGGGCGGG-CCCCCCTCCCCCCACCCCCACG
 12 Orf_virus_NP                                  24.1%  23.5%     --------------------------------------------------------------------------------
    consensus/100%                                                ................................................................................
    consensus/90%                                                 GGCAAGCAGTTGGATACGGCGGGGCGGGGCACGACGTGCACGGAGAuTTCGGGCGGG..CCCCsTCCCCCCACCCCCACG
    consensus/80%                                                 GGCAAGCAGTTGGATACGGCGGGGCGGGGCACGACGTGCACGGAGAATTCGGGCGGG..CCCCsTCCCCCCACCCCCACG
    consensus/70%                                                 GGCAAGCAGTTGGATACGGCGGGGCGGGGCACGACGTGCACGGAGAATTCGGGCGGG..CCCCsTCCCCCCACCCCCACG

                                                      cov    pid 5601          .         .         .         .         :         .         .         . 5680
  1 Orf_virus_OV-SA00_NC_005336.1_115015-122053 100.0% 100.0%     CACCACGATGCGTCTAATCTTAGCGCTCGTGGCCTGCTTGTTGGCGGCGCCGATGCCGTTATCGGGTCGTTCGACAAGCA
  2 Orf_virus_D1701                               98.3%  88.3%     CACCACGATGCGTCTAATCTTAGCGCTTGTGGCCTGCTTGTTGGCGGCGCCGATGCCGTTATCGGGTCGTTCGACAAGCA
  3 Orf_virus_NA1-11                              97.9%  87.5%     CACCACGATGCGTCTAATCTTAGCGCTCGTGGCCTGCTTGTTGGCGGCGCCGATGCCGTTATCGGGTCGTTCGACAAGCA
  4 Orf_virus_OV-HN3_12                           98.1%  87.2%     CACCACGATGCGTCTAATCTTAGCGCTCGTGGCCTGCTTGTTGGCGGCGCCGATGCCGTTATCGGGTCGTTCGACAAGCA
  5 Orf_virus_OV-IA82                             98.6%  88.5%     CACCACGATGCGTCTAATCTTAGCGCTCGTGGCCTGCTTGTTGGCGGCGCCGATGCCGTTATCGGGTCGTTCGACAAGCA
  6 Orf_virus_NZ2                                 98.5%  88.2%     CACCACGATGCGCCTAATCTTAGCGCTCGTGGCCTGCTTGTTGGCGGCGCCGATGCCGTTATCGGGTCGTTCGACAAGCA
  7 Orf_virus_YX                                  98.1%  95.5%     CACCACGATGCGTCTAATTTTAGCGCTCGTGGCTTGCTTGTTGGCGGCGCCGATGCCGTTATCGGGTCGTACGACAAGCA
  8 Orf_virus_GO                                  98.8%  96.4%     CACCACGATGCGCCTAATCTTAGCGCTCGTGGCCTGCTTGTTGGCGGCGCCGATGCCGTTATCGGGTCGTACGACAAGCA
  9 Orf_virus_B029                                91.8%  81.7%     CACCACGATGCGCCTAATCTTAGCGCTCGTGGCCTGCTTGTTGGCGGCGCCGATGCCGTTATCGGGTCGTTCGACAAGCA
 10 Orf_virus_IHUMI-1                             78.5%  70.9%     CACCACGATGCGTCTAATCTTAGCGCTCGTGGCCTGCTTGTTGGCGGCGCCGATGCCGTTATCGGGTCGTACGACAAGCA
 11 Orf_virus_SJ1                                 77.2%  74.2%     CACCACGATGCGTCTAATCTTAGCGCTCGTGGCCTGCTTGTTGGCGGCGCCGATGCCGTTATCGGGTCGTTCGACAAGCA
 12 Orf_virus_NP                                  24.1%  23.5%     -----------------------------------------------------------------------CGACTAGC-
    consensus/100%                                                .......................................................................CGACsAGC.
    consensus/90%                                                 CACCACGATGCGsCTAATsTTAGCGCTsGTGGCsTGCTTGTTGGCGGCGCCGATGCCGTTATCGGGTCGTsCGACAAGCA
    consensus/80%                                                 CACCACGATGCGsCTAATCTTAGCGCTCGTGGCCTGCTTGTTGGCGGCGCCGATGCCGTTATCGGGTCGTsCGACAAGCA
    consensus/70%                                                 CACCACGATGCGsCTAATCTTAGCGCTCGTGGCCTGCTTGTTGGCGGCGCCGATGCCGTTATCGGGTCGTsCGACAAGCA

                                                      cov    pid 5681          .         7         .         .         .         .         :         . 5760
  1 Orf_virus_OV-SA00_NC_005336.1_115015-122053 100.0% 100.0%     CCCCAAACACA---CCGTCCGCACTCGGCTCGACGAGTGCGACACCAAGCTCGGAAGACGCTGCGGCTTCGAGCACAACG
  2 Orf_virus_D1701                               98.3%  88.3%     CTCCAAACACGTCCCCCTCCGTACTCGGCTCGACGAGTTCGGAACCAAGCTCGGAAGACGCTGTGGCTTCGAGCACAACG
  3 Orf_virus_NA1-11                              97.9%  87.5%     CTCCAAACACGTCCCTCTCCGTACTCGGCTCGACGAGTTCGGAACCAAGCTCGGAAGACGCTGTGGCTTTGAGCACAACG
  4 Orf_virus_OV-HN3_12                           98.1%  87.2%     CTCCAAACACGTCCCTCTCCGCACTCGGCTCGACGAGTTCGGAACCAAGCTCGGAAGACGCTGTGGCTTCGAGCACAACG
  5 Orf_virus_OV-IA82                             98.6%  88.5%     CCCCAAACACA---CAGTCCGTACTCGGCTCGACGAGTTCGGAACCAAGCTCGGAAGACGCTGTGGCTTCGAGCACAACG
  6 Orf_virus_NZ2                                 98.5%  88.2%     CTCCAAACACGTCCCCCTCCGCACTCGGCTCGACGAGTTCGGAACCAAGCTCGGAAGACGCTGTGGCTTCGAGCACAACG
  7 Orf_virus_YX                                  98.1%  95.5%     CCCCAAATTTA---CAGTCCGCACTCGGCTCGACGAGTTCGGCACAAAGCTCTGAAGACGCAGTGTCTTCGAGCACAACG
  8 Orf_virus_GO                                  98.8%  96.4%     CCCCAAACACA---CCGTCCGCACTCGGCTCGACGAGTACGGCACCAAGCTCGGAAGACGCTGTGGCTTCGAGCACAACG
  9 Orf_virus_B029                                91.8%  81.7%     CTCCAAACACGTCCCCCTCCGCACTCGGCTCGACGAGTTCGGAAACAAGCTCGGAAGACGCTGTGGCTTCGAGCACAACG
 10 Orf_virus_IHUMI-1                             78.5%  70.9%     CTCCAAACACGTCCCCCTCCGTACTCGGCTCGACGAGTTCGGAACCAAGCTCGGAAGACGCTGTGGCTTCGAGCACACTC
 11 Orf_virus_SJ1                                 77.2%  74.2%     CCCCAAATTTA---CAGTCCGCACTCGGCTCGACGAGTTCGGAACCAAGCTCGGAAGACGCTGCGGCTTCGAGCACAACG
 12 Orf_virus_NP                                  24.1%  23.5%     --------------------------------------------------------------------------------
    consensus/100%                                                ................................................................................
    consensus/90%                                                 CsCCAAAsssu...CssTCCGsACTCGGCTCGACGAGTsCGusAssAAGCTCsGAAGACGCsGsGsCTTsGAGCACAsss
    consensus/80%                                                 CsCCAAAsssu...CssTCCGsACTCGGCTCGACGAGTsCGGsACCAAGCTCGGAAGACGCTGsGGCTTCGAGCACAACG
```

```
   consensus/70%                                                   CsCCAAACACu...CssTCCGsACTCGGCTCGACGAGTTCGGsACCAAGCTCGGAAGACGCTGTGGCTTCGAGCACAACG

                                                      cov    pid 5761         .         .         .         8         .         .         .         . 5840
 1 Orf_virus_OV-SA00_NC_005336.1_115015-122053 100.0% 100.0%     ACAAGCAC------------TACAAGCACACTCACTTCGTCCACAAGTGTGGACACCACTACTACCTCGGGCGCTACGAC
 2 Orf_virus_D1701                              98.3%  88.3%     ACAAGCAC------------TACAAGCACACTCACTCTGTCCACAAGTGTGGACACCACTACTACCTCGGGCGCTACAAC
 3 Orf_virus_NA1-11                             97.9%  87.5%     ACAAGCACACT---------------------CACTCTGTCCACAAATGTGGACACCACTACTACCTCGGGCGCTACAAC
 4 Orf_virus_OV-HN3_12                          98.1%  87.2%     ACAAGCACACTCACAAGCACTACAAGCACACGCACTCTGTCCACAAGTGTGGACACCACTACTA---CGGGCGCTACAAC
 5 Orf_virus_OV-IA82                            98.6%  88.5%     ACAAGCACACTCACAAGCACTACAAGCACACTCACTATGTCCACAAGTGTGGACACCACTACTACCTCGGGCGCTACGAC
 6 Orf_virus_NZ2                                98.5%  88.2%     ACAAGCACACTCACAAGCACTACAAGCACACTCACTATGTCCACAAGTGTGGACACCACTACTACCTCGGGCGCTACAAC
 7 Orf_virus_YX                                 98.1%  95.5%     ACAAGCACACT---------------------CACTTTGTCCACAAGTGTGGACACCACTACTACCTCGGGCGCTACGAC
 8 Orf_virus_GO                                 98.8%  96.4%     ACAAGCACACT---------------------CACTTTGCCCACAAGTGTGGACACCACTACTACCTCGGGCGCTACGAC
 9 Orf_virus_B029                               91.8%  81.7%     ACAAGCACACT---------------------CACTATGTCCACAAGTGTGGACACCACTACTACCTCTGGCGCTACAAC
10 Orf_virus_IHUMI-1                            78.5%  70.9%     ACAATCAC------------TACAAGCACACTCACTCTGTCCACAAGTGTGGACACCACTACTACCTCGGGCGCTACAAC
11 Orf_virus_SJ1                                77.2%  74.2%     ACAAGCACACT---------------------CACTTCGTCCACAAGTGTGGACACCACTACTACCTCGGGCGCTACGAC
12 Orf_virus_NP                                 24.1%  23.5%     --------------------------------------------------------------------------------
   consensus/100%                                                  ................................................................................
   consensus/90%                                                   ACAAsCAC.....................CACTssGsCCACAAuTGTGGACACCACTACTA...CsGGCGCTACuAC
   consensus/80%                                                   ACAAGCAC.....................CACTssGTCCACAAGTGTGGACACCACTACTACCTCGGGCGCTACuAC
   consensus/70%                                                   ACAAGCAC.....................CACTsTGTCCACAAGTGTGGACACCACTACTACCTCGGGCGCTACuAC

                                                      cov    pid 5841         :         .         .         .         .         9         .         . 5920
 1 Orf_virus_OV-SA00_NC_005336.1_115015-122053 100.0% 100.0%     GTCCA---A---CAGCACTTCTGCAGCAAGTGTGAGCTCTTCCACATCCGCAACCACTGAGGCATCGACGGCACCAACGA
 2 Orf_virus_D1701                              98.3%  88.3%     GTCCACAAACAGCAGCACTTTTGCAGCAAGTGTAAGCTCTTCCACACCCACAACCACTGAGGCATCAACCACACCAACAA
 3 Orf_virus_NA1-11                             97.9%  87.5%     GTCCACAAA---CAGCACTCCTGCAGCGAGTGTGAGCCCCTCCACACTCGCAACCACTGAGGCATCAACGGCACTAACGA
 4 Orf_virus_OV-HN3_12                          98.1%  87.2%     GTCCGCAAA---CAGCACTCCTGCAGCGAGTGTGAGCTCCTCCACACCCGCAGCCACTGAGGCATCGACGGCA-------
 5 Orf_virus_OV-IA82                            98.6%  88.5%     GTCCACAAA---CAGCACTCCTGCAGCGAGTGTGAGTTCTTCCACACCCGCAGCCACTGAGGCATCGACGGCACCAACGA
 6 Orf_virus_NZ2                                98.5%  88.2%     GTCCGCAAA---CAGCACTCCTGCAGCGAGTGTGAGCTCCTCCACACCCGCAACTACCGAGGCATCGACGGCACCAACGA
 7 Orf_virus_YX                                 98.1%  95.5%     GTCCA---A---CAGCACTTCTGCAGCAAGTGTGAGCTCTTCCACACCCTCAACCACTAAGGCATCGACGGCACCAACGA
 8 Orf_virus_GO                                 98.8%  96.4%     GTCCA---A---CAGCACTTCTGCAGCAAGTGTGAGCTCTTCCACACCCGCAACCACTGAGGCATCGACGGCACCAACGA
 9 Orf_virus_B029                               91.8%  81.7%     GTCCGCAAA---CAGCACTCCTGCAGCGAGTGTGAGCTCCTCCACACCCGCAACCACTGAGGCATCGACGGCACCAACGA
10 Orf_virus_IHUMI-1                            78.5%  70.9%     GTCCACAAA---CAGCACTCCTGCAGCGAGTGTGAGTTCTTCCACACCCGCAGCTACTGAGGCATCGACGGCACCAACGA
11 Orf_virus_SJ1                                77.2%  74.2%     GTCCACACG---CAGCACTTCTGCAGCAAGTGTGAGCTCTTCCACACCCGCAACTACTGAGGCATCGACGGCACTAACGA
12 Orf_virus_NP                                 24.1%  23.5%     --------------------------------------------------------------------------------
   consensus/100%                                                  ................................................................................
   consensus/90%                                                   GTCCu...u...CAGCACTssTGCAGCuAGTGTuAGssCsTCCACAssCsCAuCsACsuAGGCATCuACsuCA.......
   consensus/80%                                                   GTCCu...A...CAGCACTsCTGCAGCuAGTGTGAGsTCsTCCACACCCuCAuCsACTGAGGCATCuACGGCACsAACuA
   consensus/70%                                                   GTCCu...A...CAGCACTsCTGCAGCuAGTGTGAGCTCsTCCACACCCGCAuCsACTGAGGCATCGACGGCACsAACGA

                                                      cov    pid 5921         .         .         :         .         .         .         .         0 6000
 1 Orf_virus_OV-SA00_NC_005336.1_115015-122053 100.0% 100.0%     CGCCGT---------TGACACCGACGAC------AGTGAAGGTAACGAATGGCAAAGAAAAC---AAGGCGTCTGCCTAC
 2 Orf_virus_D1701                              98.3%  88.3%     CGCCGTCAACGCTGACAACGCCGACAAC------AGTGAAGGTAACAAAAAACG---AGGACACGAAGGCGTCTGCCTAC
 3 Orf_virus_NA1-11                             97.9%  87.5%     CGCCGT---------CGACGCCAACGAC------AGTGAAGGTAACGAAAGACA---AAGACACGAAGGCGTCTGCCTAC
 4 Orf_virus_OV-HN3_12                          98.1%  87.2%     --CCGT---------CGACGCCGACGAC------AGTGAAGGTAACGAAGGGCAAGGAAGACACGAAGGCGTCTGCCTAC
 5 Orf_virus_OV-IA82                            98.6%  88.5%     CGCCGT---------CGACGCAGACGAC------AGTGAAGGTAACGAAAGACA---AAGACACGAAGGCGTCTGCCTAC
 6 Orf_virus_NZ2                                98.5%  88.2%     CGCCGT---------CGACGCCGACGAC------AGTGAAGGTAACGAAGGGCAAGGAAGACACGAAGGCGTCTGCCTAC
 7 Orf_virus_YX                                 98.1%  95.5%     CGCTGT---------TGACGCCAACGAC------AGTGAAGGTAACGAATGGCAAGGAAAACACGAAGGCGTCTGCCTAC
 8 Orf_virus_GO                                 98.8%  96.4%     CGCCGT---------TGACGCCGACGAC------AGTGAAGGTAACGAATGGCAAGGAAAACACGAAGGCGTCTGCCTAC
 9 Orf_virus_B029                               91.8%  81.7%     CGCCGT---------CGACGCCGACGAC------AGTGAAGGTAACGAAGGGCAAGGAAGACACGAAGGCGTCTGCCTAC
10 Orf_virus_IHUMI-1                            78.5%  70.9%     CGCCGT---------CGACGCAGACGACAGTGAAAGTGAAGGTAACGAAAGACA---AAGACACGAAGGCGTCTGCCTAC
11 Orf_virus_SJ1                                77.2%  74.2%     CGCTGT---------TGACGCCGACGAC------AGTGAAGGTAACGAATGGCAAGGAAAAC---AAGGCGTCTGCCTAC
12 Orf_virus_NP                                 24.1%  23.5%     --CCGT---------TGACGCCGACGAC------AGTGAAGGTAACGAATGGCAAGAAAAACACGAAGGCGTCTGCCTAC
   consensus/100%                                                  ..CsGT.........suACuCsuACuAC......AGTGAAGGTAACuAAsuuCu...AuuAC...AAGGCGTCTGCCTAC
   consensus/90%                                                   ..CsGT.........sGACGCsuACGAC......AGTGAAGGTAACGAAsGuCA...AAuAC...AAGGCGTCTGCCTAC
   consensus/80%                                                   CGCCGT.........sGACGCCGACGAC......AGTGAAGGTAACGAAsGuCA...AAuACACGAAGGCGTCTGCCTAC
   consensus/70%                                                   CGCCGT.........sGACGCCGACGAC......AGTGAAGGTAACGAAsGuCA...AAuACACGAAGGCGTCTGCCTAC

                                                      cov    pid 6001         .         .         .         .         :         .         .         . 6080
 1 Orf_virus_OV-SA00_NC_005336.1_115015-122053 100.0% 100.0%     CTCGCTGTACCAGTTATGTTCATGACCATGACAACGCTCGCGATGGTCGTCGTCGTGGTCGTGCTCATGTACAAACAGGG
 2 Orf_virus_D1701                              98.3%  88.3%     CTTGTTGTACTAATTATTTTCATGGTCATGACCACCCTTGTAATGGTCGTCTTCATGGTCGTGCTCGTGTACAAACAGGG
 3 Orf_virus_NA1-11                             97.9%  87.5%     CTCGTTTTACTAATCACGTTCATGGTCATGACCACGCTAGTGATGGTCGTGGTCGTGGTCGTGGTCGTGTACAAACAGGG
 4 Orf_virus_OV-HN3_12                          98.1%  87.2%     CTCGTTTTACTAATAACGTTCATGGTCATGACCACGCTAGTGATGGTCGTGGTCGTGGTCGTGGTCGTGTACAAACAGGG
 5 Orf_virus_OV-IA82                            98.6%  88.5%     CTCGTTTTACTAATCACGTTCATGGTCATGACAACGCTAGTGATGGTTGTGGTCGTGGTCGTGATCGTGTACAAACAGGG
 6 Orf_virus_NZ2                                98.5%  88.2%     CTCGTTTTACTAATCACGTTCATGGTCATGACCACGCTCGTGATGGTCGTGGTCGTGGTCGTGGTCGTGTACAAACAGGG
 7 Orf_virus_YX                                 98.1%  95.5%     CTCGCTGTACCAGTTATGTTCATGACCATGACCACGCTCGCGATGGTCGTCGTTGTGGTCGTGCTCATGTACAAACAGGG
 8 Orf_virus_GO                                 98.8%  96.4%     CTCGCTGTACCAGTTATGTTCATGACCATGACCACGCTCGCGATGGTCGTCGTCGTGGTTGTGCTCATGTACAAACAGGG
 9 Orf_virus_B029                               91.8%  81.7%     CTCGTTTTACTAATCACGTTCATGGTCATGACCACGCTCGTGATGGTCGTGGTCGTGGTCGTGGTTGTGTACAAACAGGG
10 Orf_virus_IHUMI-1                            78.5%  70.9%     CTCGTTTTACTAATCACGTTCATGGTCATGACCACGCTCGTGATGGTCGTGGTCGTGGTCGTGGTCATGTACAAACAGGG
11 Orf_virus_SJ1                                77.2%  74.2%     CTCGCTGTACCAGTTATGTTCATGACCATGACCACGCTCGCGATGGTCGTCGTCGTGGTTGTGCTCATGTACAAACAGGG
12 Orf_virus_NP                                 24.1%  23.5%     CTCGCTGTACCAGTTATGTTCATGACCATGACCACGCTCGCGATGGTCGTCGTTGTGGTCGTGCTCATGTACAAACAGGG
   consensus/100%                                                  CTsGsTsTACsAuTsAssTTCATGusCATGACsACsCTsGsuATGGTsGTssTsuTGGTsGTGsTsuTGTACAAACAGGG
   consensus/90%                                                   CTCGsTsTACsAuTsAsGTTCATGusCATGACsACGCTsGsGATGGTCGTsGTsGTGGTsGTGsTCuTGTACAAACAGGG
   consensus/80%                                                   CTCGsTsTACsAuTsAsGTTCATGusCATGACCACGCTsGsGATGGTCGTsGTCGTGGTCGTGsTCuTGTACAAACAGGG
   consensus/70%                                                   CTCGsTsTACsAuTsAsGTTCATGusCATGACCACGCTsGsGATGGTCGTsGTCGTGGTCGTGsTCuTGTACAAACAGGG

                                                      cov    pid 6081         .         1         .         .         .         .         :         . 6160
 1 Orf_virus_OV-SA00_NC_005336.1_115015-122053 100.0% 100.0%     ACTCTGCAACTGCTTCTG-TAAGATGTTTCCCTGCTGCAAAGAACTCAAGGACTACCTCGACGAGGAGGAGAGCGCCGGG
 2 Orf_virus_D1701                              98.3%  88.3%     ACTCTGCAACTGCTGCTGTTAACATGTTTCCCTGCTGCGACAAGCTCAAGGACTACCTCCACAAGGAGGAGAGCGCCGGG
 3 Orf_virus_NA1-11                             97.9%  87.5%     ACTCTGTAACTGCTGCTG-TAGGATGTTTCCCTGCTGCAAAGAGATCAAGGACTACCTCGACGAGGAGGAGAGCGCCGGG
 4 Orf_virus_OV-HN3_12                          98.1%  87.2%     ACTCTGTAACTGCTGCTG-TAGGATGTTTCCCTGCTGCAAAGAGATCAAGGACTACCTCGACGAGGAGGAGAGCGCCGGG
 5 Orf_virus_OV-IA82                            98.6%  88.5%     ACTTTGTGACTGCTGCTG-TAAGATGTTTCCCTGCTGCAAAGAGCTCAAGGACTACCTCGACGAGGAGGAGAGCGCCGGG
 6 Orf_virus_NZ2                                98.5%  88.2%     ACTCTGTAACTGCTGCTG-TAA---GTTTCCCTGCTGCAAAGAGCTCAAGGACTACCTCGACGAGGAGGAGAGCGCCGGG
 7 Orf_virus_YX                                 98.1%  95.5%     ACTCTGTAACTGCTTCTG-TAAGATGTTTCCCTGCTGCAAAGAACTCAAGGACTACCTCGACGAGGAGGAGAGCGCCGGG
 8 Orf_virus_GO                                 98.8%  96.4%     ACTCTGCAACTGCTTCTG-TAAGATGTTTCCCTGCTGCAAAGAACTCAAGGACTACCTCGACGAGGAGGAGAGCGCCGGG
 9 Orf_virus_B029                               91.8%  81.7%     ACTCTGCAACTGCTGCTG-TAAGATGTTTCCCTGCTGCAAAGAGCTCAAGGACTACCTCGACGAGGAGGAGAGCGCCGGG
10 Orf_virus_IHUMI-1                            78.5%  70.9%     ACTCTGTGACTGCTGCTG-TAA---GTTTCCCTGCTGCAAAGAGCTCAAGGACTACCTCGACGAGGAGGAGAGCGCCGGG
11 Orf_virus_SJ1                                77.2%  74.2%     ACTCTGCAACTGCTTCTG-TAAGATGTTTCCCTGCTGCAAAGAACTCAAGGACTACCTCGACGAGGAGGAGAGCGCCGGG
12 Orf_virus_NP                                 24.1%  23.5%     ACTCTGCAACTGCTTCTG-TAAGATGTTTCCCTGCTGCAAAGAACTCAAGGACTACCTCGACGAGGAGGAGAGCGCCGGG
   consensus/100%                                                  ACTsTGsuACTGCTsCTG.TAu...GTTTCCCTGCTGCuAsuAusTCAAGGACTACCTCsACuAGGAGGAGAGCGCCGGG
   consensus/90%                                                   ACTCTGsuACTGCTsCTG.TAu...GTTTCCCTGCTGCAAAGAusTCAAGGACTACCTCGACGAGGAGGAGAGCGCCGGG
   consensus/80%                                                   ACTCTGsAACTGCTsCTG.TAAsATGTTTCCCTGCTGCAAAGAuCTCAAGGACTACCTCGACGAGGAGGAGAGCGCCGGG
   consensus/70%                                                   ACTCTGsAACTGCTsCTG.TAAGATGTTTCCCTGCTGCAAAGAuCTCAAGGACTACCTCGACGAGGAGGAGAGCGCCGGG

                                                      cov    pid 6161         .         .         .         2         .         .         .         . 6240
 1 Orf_virus_OV-SA00_NC_005336.1_115015-122053 100.0% 100.0%     CTGTACGACGCCGTGACGTGGAGCCACTCGAACCCCGGCTTCCGGCTCGTCACGCGCACAGACCCCAGATGATGAAGATC
 2 Orf_virus_D1701                              98.3%  88.3%     CTGTACGACGCCGTGACGTGGAGCCACTCGAACCCCGGCTTCCGGCTTGTCACGCGCGCGGACCCCAGATGATGAGGATC
 3 Orf_virus_NA1-11                             97.9%  87.5%     CTGTACGACTCCTTGACGTGGAGCCCCTCGAAACCCGGCTTCCGGCTCGTCGTGCGCGAGGACCCCAGATGATGAGGATC
 4 Orf_virus_OV-HN3_12                          98.1%  87.2%     CTGTACGACTCCTTGACGTGGAGCCCCTCGAAACCCGGCTTCCGGCTCGTCGTGCGCGAGGACCCCAGATGATGAGGATC
 5 Orf_virus_OV-IA82                            98.6%  88.5%     CTGTACGACGCCTTGACGTGGAGCCGCTCAGACCCCGGCCTCCGGCTCGTCGTGCGCGCGGACCCCAGATGATGAGGATC
 6 Orf_virus_NZ2                                98.5%  88.2%     CTGTACGACGCCTTGACGTGGAGCCACTCAGACTCCGGCCTCCGGCTCGTCGTGCGCGCGGACCCCAGATGATGAGGATC
 7 Orf_virus_YX                                 98.1%  95.5%     CTGTACGACGCCGTGACGTGGAGCCACTCGAACCCCGGCTTCCGGCTCGTCACGCGCACAGACCCCAGATGACGAAGATC
 8 Orf_virus_GO                                 98.8%  96.4%     CTGTACGACGCCGTGACGTGGAGCCACTCAAACCCCGGCTTCCGGCTCGTCACGCGCACAGACCCCAGATGACGAAGATC
 9 Orf_virus_B029                               91.8%  81.7%     CTGTACGACGCCTTGACGTGGAGCCACTCGAACCCCGGCCTCCGGCTCGTCGTGCGCGCGGACCCCAGATGATGAGGATC
10 Orf_virus_IHUMI-1                            78.5%  70.9%     CTGTACGACGCCTTGACGTGGAGCCGCTCAGACCCCGGCCTCCGGCTCGTCGTGCGCGCGGACCCCAGATGATGAGGATC
11 Orf_virus_SJ1                                77.2%  74.2%     CTGTACGACGCCGTGACGTGGAGCCACTCGAACCCCGGCTTCCGGCTCGTCACGCGCACAGACCCCAGATGATGAAGATC
12 Orf_virus_NP                                 24.1%  23.5%     CTGTACGACGCCGTGACGTGGAGCCACTCAAACCCCGGCTTCCGGCTCGTCACGCGCACAGACCCCAGATGATGAAGATC
   consensus/100%                                                  CTGTACGACsCCsTGACGTGGAGCCsCTCuuAssCCGGCsTCCGGCTsGTCusGCGCusuGACCCCAGATGAsGAuGATC
   consensus/90%                                                   CTGTACGACsCCsTGACGTGGAGCCsCTCuuAsCCCGGCsTCCGGCTCGTCusGCGCusuGACCCCAGATGAsGAuGATC
   consensus/80%                                                   CTGTACGACGCCsTGACGTGGAGCCuCTCuuACCCCGGCsTCCGGCTCGTCusGCGCuCuGACCCCAGATGATGAuGATC
   consensus/70%                                                   CTGTACGACGCCsTGACGTGGAGCCuCTCuAACCCCGGCsTCCGGCTCGTCusGCGCuCuGACCCCAGATGATGAuGATC

                                                      cov    pid 6241         :         .         .         .         .         3         .         . 6320
```

```
 1 Orf_virus_OV-SA00_NC_005336.1_115015-122053 100.0% 100.0%      GGATCAGATCGGCAACTGTTATTTGTCACGCCCGCCGCGAACATTATGCCTCTAAATGCTGAGAATTAACTGAAATTCA
 2 Orf_virus_D1701                              98.3%  88.3%      TTATCCGATCGGCAACCG-TGTTTTGTCCCGCCCGTCGCGAACATTATGCCTCTAAATGCTGAGAATTAACTGAAATTCA
 3 Orf_virus_NA1-11                             97.9%  87.5%      GGATAAGATTGG----CG-TGTTTT-TCCCGCCCGTCGCGAACATTATGCCTCTAAATGCCGAGAATTAACTGAAATTCA
 4 Orf_virus_OV-HN3_12                          98.1%  87.2%      GGATAAGATTGG----CG-TGTTTT-TCCCGCCCGTCGCGAACATTATGCCTCTAAATGCCGAGAATTAACTGAAATTCA
 5 Orf_virus_OV-IA82                            98.6%  88.5%      GGATAAGATCGG----CG-TGTTTT-TCCCGCCCGTCGCGAACATTATGCCTCTAAATGCCGAGAATTAACTGAAATTCA
 6 Orf_virus_NZ2                                98.5%  88.2%      GGATAAGATCGG----CG-TGTTTT-TCCCGCCCGTCGCGAACATTATGCCTCTAAATGCCGAGAATTAACTGAAATTCA
 7 Orf_virus_YX                                 98.1%  95.5%      GGATCAGATTGGCAACC--TGTTTTATCCCGCCCGTCGCGAACATTATGCCTCTAAATGCTGAGAATTAACTGAAATTCA
 8 Orf_virus_GO                                 98.8%  96.4%      GGATCAGATCGGCAACC--TGTTTTATCCCGCCCGTCGCGAACATTATGCCTCTAAATGCTGAGAATTAACTGAAATTCA
 9 Orf_virus_B029                               91.8%  81.7%      GGATCAGATCGG----CG-TGTTTT-TCCCGCCCGTCGCGAACATTATGCCTCTAAATGCCGAGAATTAACTGAAATTCA
10 Orf_virus_IHUMI-1                            78.5%  70.9%      GGATCAGATCGG----CG-TGTTTT-TCCCGCCCGTCGCGAACATTATGCCTCTAAATGCCGAGAATTAACTGAAATTCA
11 Orf_virus_SJ1                                77.2%  74.2%      GGATCAGATCGGCAACTGTTATTTGTCCCGCCCGCCGCGAACATTATGCCTCTAAATGCTGAGAATTAACTGAAATTCA
12 Orf_virus_NP                                 24.1%  23.5%      G-----GATCGGCAACC--TGTTTTATCCCGCCCGCCGCGAACATTATGCCTCTAAATGCTGAGAATTAACTGAAATTCA
   consensus/100%                                                 s.....GATsGG....s..TuTTTT.TCsCGCCCGsCGCGAACATTATGCCTCTAAATGCsGAGAATTAACTGAAATTCA
   consensus/90%                                                  GsATssGATsGG....s..TuTTTT.TCCCGCCCGsCGCGAACATTATGCCTCTAAATGCsGAGAATTAACTGAAATTCA
   consensus/80%                                                  GGATsAGATsGG....C..TGTTTT.TCCCGCCCGsCGCGAACATTATGCCTCTAAATGCsGAGAATTAACTGAAATTCA
   consensus/70%                                                  GGATsAGATCGG....CG.TGTTTT.TCCCGCCCGTCGCGAACATTATGCCTCTAAATGCsGAGAATTAACTGAAATTCA

                                                   cov    pid 6321         .         .         :         .         .         .         .         4 6400
 1 Orf_virus_OV-SA00_NC_005336.1_115015-122053 100.0% 100.0%      AACACGCTTTGGGATTCAACTCCGCGGCCCACACGCAACCATGGCTGGCTTCCTAGGTGCGTTCAGAGGCGTGTGCTCCG
 2 Orf_virus_D1701                              98.3%  88.3%      AACACGCTTTGGGGTTCAACTCCGTGACCCACA--CAACCATGGCTGGCTTCCTAGGCGCGTTCAGAGGCGTGTGCTGCG
 3 Orf_virus_NA1-11                             97.9%  87.5%      AACACGCTTTGGGACTCAACTCTGTGGCCCACA--CAACCATGGCTGGCTTCCTAGGCGCGTTCAGAGGCGTGTGCTCTG
 4 Orf_virus_OV-HN3_12                          98.1%  87.2%      AACACGCTTTGGGACTCAACTCTGTGGCCCACA--CAACCATGGCTGGCTTCCTAGGCGCGTTCAGAGGCGTGTGCTCTG
 5 Orf_virus_OV-IA82                            98.6%  88.5%      AACACGCTTTGGGACTCAACTCTGTGGCCCACA--CAACCATGGCTGGCTTCCTAGGCGCGTTCAGAGGCGTGTGCTCCG
 6 Orf_virus_NZ2                                98.5%  88.2%      AACACGCTTTGGGACTCAACTCCGTGACCCACACTCAACCATGGCTGGCTTCCTAGGCGCATTCAGAGGCGTGTGCTCCG
 7 Orf_virus_YX                                 98.1%  95.5%      AACACGCTTTGGGATTCAACTCCGCGGCCCACACGCAACCATGGCTGGCTTCCTAGGTGCGTTCAGAGGCGTGTGCTCCG
 8 Orf_virus_GO                                 98.8%  96.4%      AACACGCTTTGGGATTCAACTCCGCGGCCCACACGCAACCATGGCTGGCTTCCTAGGTGCGTTCAGAGGCGTGTGCTCCG
 9 Orf_virus_B029                               91.8%  81.7%      AACACGCTTTGGGACTCAACTCTGTGGCCCACT--CAACCATGGCTGGCTTCCTAGGCGCGTTCAGAGGCGTGTGCTCCG
10 Orf_virus_IHUMI-1                            78.5%  70.9%      AACACGCTTTGGGACTCAACTCTGTGGCCCACA--CAACCATGGCTGGCTTCCTAGGCGCGTTCAGAGGCGTGTGCTCCG
11 Orf_virus_SJ1                                77.2%  74.2%      AACACGCTTTGGGACTCAACTCCGCGGCCCACACGCAACCATGGCTGGCTTCCTAGGTGCGTTCAGAGGCGTGTGCTCCG
12 Orf_virus_NP                                 24.1%  23.5%      AACACGCTTTGGGATTCAACTCTGCGGCCCACACGCAACCATGGCTGGCTTCCTAGGCGCGTTCAGAGGCGTGTGTTCAG
   consensus/100%                                                 AACACGCTTTGGGusTCAACTCsGsGuCCCACs..CAACCATGGCTGGCTTCCTAGGsGCuTTCAGAGGCGTGTGsTssG
   consensus/90%                                                  AACACGCTTTGGGAsTCAACTCsGsGuCCCACA..CAACCATGGCTGGCTTCCTAGGsGCGTTCAGAGGCGTGTGCTCsG
   consensus/80%                                                  AACACGCTTTGGGAsTCAACTCsGsGGCCCACA..CAACCATGGCTGGCTTCCTAGGsGCGTTCAGAGGCGTGTGCTCsG
   consensus/70%                                                  AACACGCTTTGGGAsTCAACTCsGsGGCCCACA..CAACCATGGCTGGCTTCCTAGGsGCGTTCAGAGGCGTGTGCTCCG

                                                   cov    pid 6401         .         .         .         .         :         .         .         . 6480
 1 Orf_virus_OV-SA00_NC_005336.1_115015-122053 100.0% 100.0%      AAATATGGCAGTCGCTCCGTGGACACGGACACCACCACTCTTCCAACTGTCCGCGACGACGCGCCAACAGCATGGACGAG
 2 Orf_virus_D1701                              98.3%  88.3%      AAATATGGCAGTCGCTCCGTGGACAGGGACACCACCACTCGTCTAACTGCCCGCGACAACGCGCCAATAGCGTGGACGAC
 3 Orf_virus_NA1-11                             97.9%  87.5%      ACTTATGGCAGTCGCTCCGTGGACACGGA---CACCACTCTTC---CTGCCCGCGACGACGCGCCAACAGCATGGACGAC
 4 Orf_virus_OV-HN3_12                          98.1%  87.2%      ACTTATGGCAGTCGCTCCGTGGACACGGA---CACCACTCTTC---CTGCCCGCGACGACGCGCCAACAGCATGGACGAC
 5 Orf_virus_OV-IA82                            98.6%  88.5%      ACTTATGGCAGTCGCTCCGTGGACACGGA---CACCACTCTTCCAGCTGCCCGCGACGACGCGCCAACAGCATGGACGAC
 6 Orf_virus_NZ2                                98.5%  88.2%      ACTTATGGCAGTCGCTCCGTGGACACGGA---CACCACTCTTCCAGCTGCCCGCGACGACGCGCCAACAGCATGGACGAC
 7 Orf_virus_YX                                 98.1%  95.5%      AAATATGGCAGTCGCTCCGTGGACACGGACACCACCACTCTTCCAACTGCCCGCGACGACGCGCCAACAGCGTGGACGAA
 8 Orf_virus_GO                                 98.8%  96.4%      AAATATGGCAGTCGCTCCGTGGACACGGACACCACCACTCTTCCAACTGCCCGCGACGACGCGCCAACAGCGTGGACGAA
 9 Orf_virus_B029                               91.8%  81.7%      ACTTATGGCAGTCGCTCCGTGGACACGGA---CACCACTCTTCCAGCTGCCCGCGACGACGCGCCAACAGCATGGACGAC
10 Orf_virus_IHUMI-1                            78.5%  70.9%      ACTTATGGCAGTCGCTCCGTGGACACGGA---CACCACTCTTCCAGCTGCCCGCGACGACGCGCCAACAGCATGGACGAC
11 Orf_virus_SJ1                                77.2%  74.2%      AAATATGGCAGTCGCTCCGTGGACACGGACACCACCACTCCTCCAACTGCCCGCGACGACGCGCCAACAGCATGGACGAG
12 Orf_virus_NP                                 24.1%  23.5%      AAATATGGCAATCGCTCCGTGGACACGGACACCACCACTCTTCCAACTGCCCGCGACGACGCGCCAACAGCGTGGACGAA
   consensus/100%                                                 AssTATGGCAuTCGCTCCGTGGACAsGGA...CACCACTCsTC...CTGsCCGCGACuACGCGCCAAsAGCuTGGACGAs
   consensus/90%                                                  AssTATGGCAGTCGCTCCGTGGACACGGA...CACCACTCsTC...CTGCCCGCGACGACGCGCCAACAGCuTGGACGAs
   consensus/80%                                                  AssTATGGCAGTCGCTCCGTGGACACGGA...CACCACTCTTCsAuCTGCCCGCGACGACGCGCCAACAGCuTGGACGAs
   consensus/70%                                                  AssTATGGCAGTCGCTCCGTGGACACGGA...CACCACTCTTCCAuCTGCCCGCGACGACGCGCCAACAGCuTGGACGAs

                                                   cov    pid 6481         .         5         .         .         .         .         :         . 6560
 1 Orf_virus_OV-SA00_NC_005336.1_115015-122053 100.0% 100.0%      CGCGACCGGCGCCGGCACCGCCACC------GCGAGATCCCCGACAGCTCGGCGTCGCTGAACCGCGACCTGATGCCGCG
 2 Orf_virus_D1701                              98.3%  88.3%      CGCGACCGGCGCCGGCGCCGCCACCGCCACTGCGAGATCCCCGACAGCTCAGTGTCGCTGAACCGCGACCTGATGCCGCG
 3 Orf_virus_NA1-11                             97.9%  87.5%      CGCGACCGGCGCCGACACCGCCACC------GCGAGATCCCCAACAGCTCGGCGTCGCTGAACAGCGACCCGATGCCGCA
 4 Orf_virus_OV-HN3_12                          98.1%  87.2%      CGCGACCGGCGCCGACACCGCCACC------GCGAGATCCCCAACAGCTCGGCGTCGCTGAACAGCGACCCGATGCCGCA
 5 Orf_virus_OV-IA82                            98.6%  88.5%      CGCGACCGGCGCCGGCACCGCCACC------GCGAGATCCCCAACAGCTCGGCGTCGCTGAACAGCGACCCGATGCCGCC
 6 Orf_virus_NZ2                                98.5%  88.2%      CGCGACCGGCGCCGACACCGCCACC------GCGAGATCCCCAACAGCTCGGCGTCGCTGAACAGCGACCCGATGCCGCA
 7 Orf_virus_YX                                 98.1%  95.5%      CGCGACCGGCGCCGGCACCGCCACC------GCGAGATCCCCGACAGCTCGGCGTCGCTGAACCGCGACCTGATGCCGCG
 8 Orf_virus_GO                                 98.8%  96.4%      CGCGACCGGCGCCGGCACCGCCACC------GCGAGATCCCCGACAGCTCGGCGTCGCTGAACCGCGACCTGATGCCGCG
 9 Orf_virus_B029                               91.8%  81.7%      CGCGACCGGCGCCGGCACCGCCACC------GCGAGATCCCCAACAGCTCGGCGTCGCTGAACAGCGACCCGATGCCGCC
10 Orf_virus_IHUMI-1                            78.5%  70.9%      CGCGACCGGCGCCGGCACCGCCACC------GCGAGATCCCCAACAGCTCGGCGTCGCTGAACAGCGACCCGATGCCGCA
11 Orf_virus_SJ1                                77.2%  74.2%      CGCGACCGGCGCCGGCACCGCCACC------GCGAGATCCCCGACAGCTCGGCGTCGCTGAACCGCGACCTGATGCCGCG
12 Orf_virus_NP                                 24.1%  23.5%      CGCGACCGGCGCCGGCACCGCCACC------GCGAGATCCCCGACAGCTCGTCGTCGCTGAACCGCGACCTGATGCCGCG
   consensus/100%                                                 CGCGACCGGCGCCGuCuCCGCCACC......GCGAGATCCCCuACAGCTCussGTCGCTGAACsGCGACCsGATGCCGCs
   consensus/90%                                                  CGCGACCGGCGCCGuCACCGCCACC......GCGAGATCCCCuACAGCTCGGCGTCGCTGAACsGCGACCsGATGCCGCs
   consensus/80%                                                  CGCGACCGGCGCCGuCACCGCCACC......GCGAGATCCCCuACAGCTCGGCGTCGCTGAACsGCGACCsGATGCCGCu
   consensus/70%                                                  CGCGACCGGCGCCGGCACCGCCACC......GCGAGATCCCCuACAGCTCGGCGTCGCTGAACsGCGACCsGATGCCGCu

                                                   cov    pid 6561         .         .         .         6         .         .         .         . 6640
 1 Orf_virus_OV-SA00_NC_005336.1_115015-122053 100.0% 100.0%      ACGCAGTGCGGGCGCGCTCCGGCACCACGACCGCTGCCCCTCGGAAAAGAGCAGACACTCCTCCGACAGG----------
 2 Orf_virus_D1701                              98.3%  88.3%      ACGCAGTGCGGGCGCGCTC---CACTACGACCGCTGCCCCTCGGAAAAGAGCAGACACTCCTCCGACAGG----------
 3 Orf_virus_NA1-11                             97.9%  87.5%      ACGCAGTGCGGGTGCGCGCCGACACTACGAATGCCGCCCCTCGGAAAAGAGCAGACACTCCTCCGACAGG----------
 4 Orf_virus_OV-HN3_12                          98.1%  87.2%      ACGCAGTGCGGGTGCGCGCCGACACTACGAATGCCGCCCCTCGGAAAAGAGCAGACACTCCTCCGACAGG----------
 5 Orf_virus_OV-IA82                            98.6%  88.5%      ACGCAGTGCGGGTGCGCGCCGGCACTACGACTGCCGCCCCTCGGAAAAGAGCAGACACTCCTCCGACAGG----------
 6 Orf_virus_NZ2                                98.5%  88.2%      ACGCAGTGCGGGTGCGCGCCGGCACTACGACTGCCGCCCCTCGGAAAAGAGCAGACACTCCTCCGACAAGCACCACTCGG
 7 Orf_virus_YX                                 98.1%  95.5%      ACGCAGTGCGGGCGCGCTCCGGCACCACGACCGCTGCCCCTCGGAAAAGAGCAGACACTCCTCCGACAGG----------
 8 Orf_virus_GO                                 98.8%  96.4%      ACGCAGTGCGGGCGCGCTCCGGCACCACGACCGCTGCCCCTCGGAAAAGAGCAGACACTCCTCCGACAGG----------
 9 Orf_virus_B029                               91.8%  81.7%      ACGCAGTGCGGGTGCGCGCCGGCACTACGACTGCCGCCCCTCGGAAAAGAGCAGACACTCCTCCGACAGG----------
10 Orf_virus_IHUMI-1                            78.5%  70.9%      ACGCAGTGCGGGTGCGCGCCGGCACTACGACTGCCGCCCCTCGGAAAAGAGCAGACACTCCTCCGACAAG----------
11 Orf_virus_SJ1                                77.2%  74.2%      ACGCAGTGCGGGCGCGCTCCGGCACCACGACCGCTGCCCCTCGGAAAAGAGCAGACACTCCTCCGACAGG----------
12 Orf_virus_NP                                 24.1%  23.5%      ACGCAGTGCGGGCGCGCTCCGGCACCACGACCGCTGCCCCTCGGAAAAGAGCAGACACTCCTCCGACAGG----------
   consensus/100%                                                 ACGCAGTGCGGGsGCGCsC...CACsACGAssGCsGCCCCTCGGAAAAGAGCAGACACTCCTCCGACAuG..........
   consensus/90%                                                  ACGCAGTGCGGGsGCGCsCCGuCACsACGAssGCsGCCCCTCGGAAAAGAGCAGACACTCCTCCGACAuG..........
   consensus/80%                                                  ACGCAGTGCGGGsGCGCsCCGuCACsACGACsGCsGCCCCTCGGAAAAGAGCAGACACTCCTCCGACAGG..........
   consensus/70%                                                  ACGCAGTGCGGGsGCGCsCCGGCACsACGACsGCsGCCCCTCGGAAAAGAGCAGACACTCCTCCGACAGG..........

                                                   cov    pid 6641         :         .         .         .         .         7         .         . 6720
 1 Orf_virus_OV-SA00_NC_005336.1_115015-122053 100.0% 100.0%      --------CACCGCTCGGCGGACCGACACCAATCGGCGGACAGGGACCGACACCGCCGCGGTCGCAAGAACTACGACTCG
 2 Orf_virus_D1701                              98.3%  88.3%      --------CACCGCTCGGCGGACCGACACCACTCGGCGGACAGGGACCAACACCGTCGCGGTCGCAAAAACTACGACTCG
 3 Orf_virus_NA1-11                             97.9%  87.5%      --------CACCACTCGGCGGACCGACACCAATCGGCGGACAGGGAAAGACACCGTCGCAGTCGCAAGAACTACGACTCG
 4 Orf_virus_OV-HN3_12                          98.1%  87.2%      --------CACCACTCGGCGGACCGACACCAATCGGCGGACAGGGAAAGACACCGTCGCAGTCGCAAGAACTACGACTCG
 5 Orf_virus_OV-IA82                            98.6%  88.5%      --------CACCACTCGGCGGACCGACACCAATCGGCGGACAGGGACAGACACCGTCGCAGTCGCAAGAACTACGACTCG
 6 Orf_virus_NZ2                                98.5%  88.2%      CGGACCGACACCACTCGGCGGACCGACACCAATCGGCGGACAGGGACAGACACCGTCGCAGTCGCAAGAACTACGACTCG
 7 Orf_virus_YX                                 98.1%  95.5%      --------CACCGCTCGGCGGACCGACACCAATCGGCGGACAGGGACCGACACCGCCGCGGTCGCAAGAACTACGACTCG
 8 Orf_virus_GO                                 98.8%  96.4%      --------CACCGCTCGGCGGACCGACACCAATCGGCGGACAGGGACCGACACCGCCGCGGTCGCAAGAACTACGACTCG
 9 Orf_virus_B029                               91.8%  81.7%      --------CACCACTCGGCGGACCGACACCAATCGGCGGACAGGGACAGACACCGTCGCAGTCGCAAGAACTACGACTCG
10 Orf_virus_IHUMI-1                            78.5%  70.9%      --------CACCACTCGGCGGACCGACACCACTCGGCGGACAGGGACAGACACCGTCGCAGTCGCAAGAACTACGACTCG
11 Orf_virus_SJ1                                77.2%  74.2%      --------CACCGCTCGGCGGACCGACACCAATCGGCGGACAGGGACCGACACCGCCGCGGTCGCAAGAACTACGACTCG
12 Orf_virus_NP                                 24.1%  23.5%      --------CACCGCTCGGCGGACCGACACCAATCAGCGGACAGAGACCGACACCGTCGCGGTCGCAAGAACTACGACTCG
   consensus/100%                                                 ........CACCuCTCGGCGGACCGACACCAsTCuGCGGACAGuGAssuACACCGsCGCuGTCGCAAuAACTACGACTCG
   consensus/90%                                                  ........CACCuCTCGGCGGACCGACACCAsTCGGCGGACAGGGAssGACACCGsCGCuGTCGCAAGAACTACGACTCG
   consensus/80%                                                  ........CACCuCTCGGCGGACCGACACCAATCGGCGGACAGGGACsGACACCGsCGCuGTCGCAAGAACTACGACTCG
   consensus/70%                                                  ........CACCuCTCGGCGGACCGACACCAATCGGCGGACAGGGACsGACACCGsCGCuGTCGCAAGAACTACGACTCG

                                                   cov    pid 6721         .         .         :         .         .         .         .         8 6800
 1 Orf_virus_OV-SA00_NC_005336.1_115015-122053 100.0% 100.0%      CACCCGTCGCGCAAGAACCGCGACTACGAGCGGGCAGACTACCAGGGATACCCCTCACAAACCCACCCAGACGCCCCCGC
 2 Orf_virus_D1701                              98.3%  88.3%      CACCCGTCGCGCAAGAACCGCAACCACGAGCGGGCAGACTACCAAAGACACCCCTCTCAAACCCACCCAGACGCCCCCGC
 3 Orf_virus_NA1-11                             97.9%  87.5%      CACCCGTCGCGCAGGAACCGCAACTACGAGCGGGAAGACTACCAGAGACACCCCTCACAAACCCACCCA---GCCCCCGC
```

```
 4 Orf_virus_OV-HN3_12                              98.1%  87.2%       CACCCGTCGCGCAGGAACCGCAACTACGAGCGGGAAGACTACCAGAGACACCCCTCACAAACCCACCCA---GCCCCCGC
 5 Orf_virus_OV-IA82                                98.6%  88.5%       CACCCGTCGCGCAGGAACCGCAACTACGAGCGGGCGGACTACCAGAGACATCCCTCACAGACCCACCCAGAAGCCCCCGC
 6 Orf_virus_NZ2                                    98.5%  88.2%       CACCCGTCGCGCAGGAACCGCAACTACGAGCGGGCGGACTACCAGAGACACCCCTCACAAACCCACCCAGACGCCCCCGC
 7 Orf_virus_YX                                     98.1%  95.5%       CACCCGTCGCGCAAGAACCGCAACTACGAGCGGGCAGACTACCAGGGACACCCCTCACAAACCCACCCAGACGCCCCCGC
 8 Orf_virus_GO                                     98.8%  96.4%       CACCCGTCGCGCAAGAACCGCAACTACGAGCGGGCAGACTACCAGGGACACCCCTCACAAACCCACCCAGACGCCCCCGC
 9 Orf_virus_B029                                   91.8%  81.7%       CACTCGACGCGCAGGAACCGCAACTACGAGCGGGCGGACTACCAGAAACATCCCTCACAGACCCACCCAGAAGCCCCCGC
10 Orf_virus_IHUMI-1                                78.5%  70.9%       CACC---CGCGCAGGAACCGCAACTACGAGCGGGTAGACTACCAGAGACATCCCTCACAGACCCGCCCAGAAGCCCCCGC
11 Orf_virus_SJ1                                    77.2%  74.2%       CACCCGTCGCGCAAGAACCGCGACTACGAGCGGGCAGACTACCAGGGATACCCCTCACAAACCCACCCAGACGCCCCCGC
12 Orf_virus_NP                                     24.1%  23.5%       CACCCGTCGCGCAAGAACCGCAACTACGAGCGGGCAGACTACCAGGGACACCCCTCACAAAACCACCCAGACGCCCCCGC
   consensus/100%                                                      CACs...CGCGCAuGAACCGCuACsACGAGCGGGsuGACTACCAuuuAsAsCCCTCsCAuAsCCuCCCA...GCCCCCGC
   consensus/90%                                                       CACCCGsCGCGCAuGAACCGCuACTACGAGCGGGsuGACTACCAGuGAsAsCCCTCACAuACCCACCCA...GCCCCCGC
   consensus/80%                                                       CACCCGTCGCGCAuGAACCGCAACTACGAGCGGGsuGACTACCAGuGACAsCCCTCACAuACCCACCCAGAsGCCCCCGC
   consensus/70%                                                       CACCCGTCGCGCAuGAACCGCAACTACGAGCGGGCAGACTACCAGuGACACCCCTCACAAACCCACCCAGAsGCCCCCGC

                                                     cov    pid  6801          .         .         .         .         :         .         .         . 6880
 1 Orf_virus_OV-SA00_NC_005336.1_115015-122053     100.0% 100.0%       GCAGACCTCGACGCTCAAGGTGACCTCCCTCAGCACCAGCTGCAGCACCCTGTCCCAACATCACTACGAGACCCCTGACC
 2 Orf_virus_D1701                                  98.3%  88.3%       GCAGACCTCGACGCTCAAAGTGACCTCCCTCAGCACCAGCTGCAGCACCCTGTCCAAACATCACTACGAGACCCCCGACC
 3 Orf_virus_NA1-11                                 97.9%  87.5%       GCAGAGTTCGACGCTCAAGGTGACCTCCCTCAGCACCAGCTGCAGCACCCTGTCCCAACATCACTACGAGACCCCCGACC
 4 Orf_virus_OV-HN3_12                              98.1%  87.2%       GCAGACTTCGACGCTCAAGGTGACCTCCCTCAGCACCAGCTGCAGCACCCTGTCCCAACATCACTACGAGACCCCCGACC
 5 Orf_virus_OV-IA82                                98.6%  88.5%       GCAGACCTCGACGCTCAAGGTGACCTCCCTCAGCACCAGCTGCAGCACCCTGTCCCAACATCACTACGAGACCCCCGACC
 6 Orf_virus_NZ2                                    98.5%  88.2%       GCAGACCTCGACGCTCAAGGTGACCTCCCTAAGCACCAGCTGCAGCACCCTGTCCCAACATCACTACGAGACCCCCGACC
 7 Orf_virus_YX                                     98.1%  95.5%       GCAGACCTCGACGCTCAAGGTGACCTCCCTCAGCACCAGCTGCAGCACCCTGTCCCAACACCACTACGAGACCCCCGACC
 8 Orf_virus_GO                                     98.8%  96.4%       GCAGACCTCGACGCTCAAGGTGACCTCCCTCAGCACCAGCTGCAGCACCCTGTCCCAACACCACTACGAGACCCCCGACC
 9 Orf_virus_B029                                   91.8%  81.7%       GCAGACCTCGACGCTCAAGGTGACCTCCCTCAGCACCAGCTGCAGCACCCTGTCCCAACATCACTACGAGACCCCCGACC
10 Orf_virus_IHUMI-1                                78.5%  70.9%       GCAGACCTCGACGCTCAAGGTGACCTCCCTCAGCACCAGCTGCAGCACCCTGTCCCAACATCACTACGAGACCCCCGACC
11 Orf_virus_SJ1                                    77.2%  74.2%       GCAGACCTCGACGCTCAAGGTGACCTCCCTCAGCACCAGCTGCAGCACCCTGTCCCAACATCACTACGAGACCCCCGACC
12 Orf_virus_NP                                     24.1%  23.5%       GCAGACCTCGACGCTCAAGGTGACCTCCCTCAGCACTAGCTGCAGCACCCTGTCCCAACATCACTACGAGACCCCCGACC
   consensus/100%                                                      GCAGAssTCGACGCTCAAuGTGACCTCCCTsAGCACsAGCTGCAGCACCCTGTCCsAACAsCACTACGAGACCCCsGACC
   consensus/90%                                                       GCAGACsTCGACGCTCAAGGTGACCTCCCTCAGCACCAGCTGCAGCACCCTGTCCCAACAsCACTACGAGACCCCCGACC
   consensus/80%                                                       GCAGACCTCGACGCTCAAGGTGACCTCCCTCAGCACCAGCTGCAGCACCCTGTCCCAACATCACTACGAGACCCCCGACC
   consensus/70%                                                       GCAGACCTCGACGCTCAAGGTGACCTCCCTCAGCACCAGCTGCAGCACCCTGTCCCAACATCACTACGAGACCCCCGACC

                                                     cov    pid  6881          .         9         .         .         .         .         :         . 6960
 1 Orf_virus_OV-SA00_NC_005336.1_115015-122053     100.0% 100.0%       ACGTCTACGACGTCCCGGAAGACAGTCGCGGGGCGTCGCTCCCCCCTTGCGCGGACCTTGCGCTCCCCCCGCTCGCCATG
 2 Orf_virus_D1701                                  98.3%  88.3%       ACATCTACGACGTCCCGGAAGACAGTCGCGGGGCGTCGGCTCCCCCTCGCGCGGACCTCGCGCTCCCCCCGCTCGCCATG
 3 Orf_virus_NA1-11                                 97.9%  87.5%       ACATCTACGACATCCCGGAAGACAGTCGCGGGGCGTCGGCTCCCCTTCGCGCGGACCTCGCGCTTCCCCCGCTCGCCATG
 4 Orf_virus_OV-HN3_12                              98.1%  87.2%       ACATCTACGACATCCCGGAAGACAGTCGCGGGGCGTCGGCTCCCCTTCGCGCGGACCTCGCGCTTCCCCCGCTCGCCATG
 5 Orf_virus_OV-IA82                                98.6%  88.5%       ACATCTACGACATCCCGGAAGACGGTCGCGGGGCGTCGGCTCCCCCTCGCGCGGACCTCGCGCTCCCCCCGCTCGCCATG
 6 Orf_virus_NZ2                                    98.5%  88.2%       ACATCTACGACATCCCGGAAGACAGTCGCGGGGCGTCGGCTCCCCCTCGCGCGGACCTCGCGCTCCCCCCGCTCGCCATG
 7 Orf_virus_YX                                     98.1%  95.5%       ACGTCTACGACGTCCCGGAAGACAGTCGCGGGGCGTCGCTCCCCCCTTGCGCGGACCTTGCGCTCCCCCCGCTCGCCATG
 8 Orf_virus_GO                                     98.8%  96.4%       ACATCTACGACGTCCCGGAAGACAGTCGCGGGGCGTCGCTCCCCCCTTGCGCGGACCTTGCGCTCCCCCCGCTCGCCATG
 9 Orf_virus_B029                                   91.8%  81.7%       ACATCTACGACATCCCGGAAGACAGTCGCGGGGCGTTGGCTCCCCCTCGCGCGGACCTCGCGCTCCCCCCGCTCGCCATG
10 Orf_virus_IHUMI-1                                78.5%  70.9%       ACATCTACGACATCCCGGAAGACAGTCGCGGGGCGTCGGCTCCCCCTCGCGCGGACCTCGCGCTCCCCCCGCTCGCCATG
11 Orf_virus_SJ1                                    77.2%  74.2%       CCGTCTACGACGTCCCGGAAGACAGTCGCGGGGCGTCGCTCCCCCCTTGCGCGGACCTTGCGCTCCCCCCGCTCGCCATG
12 Orf_virus_NP                                     24.1%  23.5%       ACGTCTACGACGTCCCGGAAGACAGTCGCGGGGCGTCGCCCCCCCCTTGCGCGGACCTTGCGCTCCCCCCGCTCGCCATG
   consensus/100%                                                      sCuTCTACGACuTCCCGGAAGACuGTCGCGGGGCGTsGsssCCCCsTsGCGCGGACCTsGCGCTsCCCCCGCTCGCCATG
   consensus/90%                                                       ACuTCTACGACuTCCCGGAAGACAGTCGCGGGGCGTCGsssCCCCsTsGCGCGGACCTsGCGCTsCCCCCGCTCGCCATG
   consensus/80%                                                       ACuTCTACGACuTCCCGGAAGACAGTCGCGGGGCGTCGsssCCCCCTsGCGCGGACCTsGCGCTCCCCCCGCTCGCCATG
   consensus/70%                                                       ACuTCTACGACuTCCCGGAAGACAGTCGCGGGGCGTCGsssCCCCCTsGCGCGGACCTsGCGCTCCCCCCGCTCGCCATG

                                                     cov    pid  6961          .         .         .         0         .         .         .         . 7040
 1 Orf_virus_OV-SA00_NC_005336.1_115015-122053     100.0% 100.0%       CCCAACTCCAAGCCCTCGCGCCGCATGCGCCCGGCGTCCATGAGCGACTTCCCAATGAAGCACTGTGGCGCCAGCAAACC
 2 Orf_virus_D1701                                  98.3%  88.3%       CCCAACTCCAAGCCCTCGCGCCGCGTGCGCCCGGCGTCCATGAACGACTGCCCGATAAAGCACTGCGGCGCCAGCAGACC
 3 Orf_virus_NA1-11                                 97.9%  87.5%       CCAAAATCCAAGCACCCGCGCCGCATGCGCCCGGCTTCCATGAACGACTGCCTGATGACGCACCGCGGCGCCGGCAAACC
 4 Orf_virus_OV-HN3_12                              98.1%  87.2%       CCAAAATCCAAGCACCCGCGCCGCATGCGCCCGGCTTCCATGAACGACTGCCTGATGACGCACCGCGGCGCCGGCAAACC
 5 Orf_virus_OV-IA82                                98.6%  88.5%       CCCAAATCCAAG---CCGCGCCGCACGCGCCCGGCGTCCATGAACGACTGCCTGATGAAGCACTGCGGCGCCGGCAGACC
 6 Orf_virus_NZ2                                    98.5%  88.2%       CCCAAATCCAAG---CCGCGTCGCACGCGCCCGGCGTCCATGAACGACTGCCTGATGAAGCACTGCGGCGCCGGCAGACC
 7 Orf_virus_YX                                     98.1%  95.5%       CCCAACTCCAAGCCCTCGCGCCGCATGCGCCCGGCGTCCATGAGCGACTTCCCAATGAAGCACTGCGGCGCCAGCAAACC
 8 Orf_virus_GO                                     98.8%  96.4%       CCCAACTCCAAGCCCTCGCGCCGCATGCGCCCGGCGTCCATGAGCGACTTCCCAATGAAGCACTGCGGCGCCAGCAAACC
 9 Orf_virus_B029                                   91.8%  81.7%       CCCAAATCCAAG---CCGCGCCGCATGCGCCCGGCGTCCATGAACGACTGCCTGATGAAGCACTGCGGCGCCGGCAGACC
10 Orf_virus_IHUMI-1                                78.5%  70.9%       CCCAAATCCAAG---CCGCGCCGCACGCGCCCGGCGTCCATGAACGACTGCCTGATGAAGCACTGCGGCGCCGGCAGACC
11 Orf_virus_SJ1                                    77.2%  74.2%       CCCAACTCTAAGCCCTCGCGCCGCATGCGCCCGGCGTCCATGAGCGACTTCCCAATGAAGCACTGTGGTGCCAGCAAACC
12 Orf_virus_NP                                     24.1%  23.5%       CCCAACTCCAAGCCCTCGCGCCGCATGCGCCCAGCGTCCATGAGCGACTTCCCAATGAAGCACTGCGGCGCCAGCAAACC
   consensus/100%                                                      CCsAAsTCsAAG...sCGCGsCGCusGCGCCCuGCsTCCATGAuCGACTsCCsuATuAsGCACsGsGGsGCCuGCAuACC
   consensus/90%                                                       CCsAAsTCCAAG...sCGCGCCGCAsGCGCCCGGCsTCCATGAuCGACTsCCsuATGAsGCACsGsGGCGCCuGCAuACC
   consensus/80%                                                       CCCAAsTCCAAG...sCGCGCCGCAsGCGCCCGGCGTCCATGAuCGACTsCCsuATGAAGCACTGCGGCGCCuGCAuACC
   consensus/70%                                                       CCCAAsTCCAAG...sCGCGCCGCATGCGCCCGGCGTCCATGAuCGACTsCCsuATGAAGCACTGCGGCGCCuGCAuACC

                                                     cov    pid  7041          :         .         .         .         .         1         .         . 7120
 1 Orf_virus_OV-SA00_NC_005336.1_115015-122053     100.0% 100.0%       CAACCTCCAAGACGACATATGCACACTATGCACTGATATAGAGACACAACTGAGTGCACTAGAGAAGTCTCTGGAGTCAG
 2 Orf_virus_D1701                                  98.3%  88.3%       CAACCTCCAAAGCGACATATGCACACTGTGCACTGATATAGAGACACAGCTGAGTGCACTAGAGAAGTCTCTGGAGTCAG
 3 Orf_virus_NA1-11                                 97.9%  87.5%       CAACCTCCAAGACGATATATGCACACTATGCACTGATATAGAGACACAGCTGAGCGCACTAGAGAAGTCTCTGGAGTCAG
 4 Orf_virus_OV-HN3_12                              98.1%  87.2%       CAACCTCCAAGACGATATATGCACACTATGCACTGATATAGAGACACAGCTGAGCGCACTAGAGAAGTCTCTGGAGTCAG
 5 Orf_virus_OV-IA82                                98.6%  88.5%       CAACCTCCAAGACGACATATGCACACTATGTACTGATATAGAGACACAGCTGAGCGCACTAGAGAAGTCTCTGGAGTCAG
 6 Orf_virus_NZ2                                    98.5%  88.2%       CAACCTCCAAGACGACATATGCACACTATGTACTGATATAGAGACACAGCTGAGCGCACTAGAGAAGTCTCTGGAGTCAG
 7 Orf_virus_YX                                     98.1%  95.5%       CAACCTCCAAGACGACATATGCACACTATGTACTGATATAGAGACACAGCTGAGTGCACTAGAGAAGTCTCTGGAGTCAG
 8 Orf_virus_GO                                     98.8%  96.4%       CAACCTCCAAGACGACATATGCACACTATGCACTGATATAGAGACACAGCTGAGTGCACTAGAGAAGTCTCTGGAGTCAG
 9 Orf_virus_B029                                   91.8%  81.7%       CAACCTCCAAGACGACATATGCACACTATGTACTGATATAGAGACACAGCTGAGCGCACTAGAGAAGTCTCTGGAGTCAG
10 Orf_virus_IHUMI-1                                78.5%  70.9%       CAACCTCCAAGACGACATATGCACACTATGTACTGATATAGAGACACAGCTGAGCGCACTAGAGAAGTCTCTGGAGTCAG
11 Orf_virus_SJ1                                    77.2%  74.2%       CAACCTCCAAGACGACATATGCACACTATGTACTGATATAGAGACACAGCTGAGTGCACTAGAGAAGTCTCTGGAGTCAG
12 Orf_virus_NP                                     24.1%  23.5%       CAACCTCCAAGACGACATATGCACACTATGTACTGATATAGAGACACAGCTGAGTGCACTAGAGAAGTCTCTGGAGTCAG
   consensus/100%                                                      CAACCTCCAAuuCGAsATATGCACACTuTGsACTGATATAGAGACACAuCTGAGsGCACTAGAGAAGTCTCTGGAGTCAG
   consensus/90%                                                       CAACCTCCAAGACGAsATATGCACACTATGsACTGATATAGAGACACAGCTGAGsGCACTAGAGAAGTCTCTGGAGTCAG
   consensus/80%                                                       CAACCTCCAAGACGACATATGCACACTATGsACTGATATAGAGACACAGCTGAGsGCACTAGAGAAGTCTCTGGAGTCAG
   consensus/70%                                                       CAACCTCCAAGACGACATATGCACACTATGsACTGATATAGAGACACAGCTGAGsGCACTAGAGAAGTCTCTGGAGTCAG

                                                     cov    pid  7121          .         .         :         .         .         .         .       2 7200
 1 Orf_virus_OV-SA00_NC_005336.1_115015-122053     100.0% 100.0%       AGCTCAACTTCTATCGTCGCTACATACAAGACACTAAGACACTACTCGCCACGCGAGCAGCAAACATCGGCAGCAAAGCT
 2 Orf_virus_D1701                                  98.3%  88.3%       AGCTCAACTTCTATCGTCGCTACATACAAGACACTAAGACATTACTCGCCACGCGAGCAGCAAACATCGGCAACAAAGCT
 3 Orf_virus_NA1-11                                 97.9%  87.5%       AGCTCAACTTCTATCGTCGCTACATACAAGACACTAAGACATTGCTCGCCACGCGAGCAGCAAACATCGGCAGCAAAGCT
 4 Orf_virus_OV-HN3_12                              98.1%  87.2%       AGCTCAACTTCTATCGTCGCTACATACAAGACACTAAGACATTGCTCGCCACGCGAGCAGCAAACATCGGCAGCAAAGCT
 5 Orf_virus_OV-IA82                                98.6%  88.5%       AGCTCAACTTCTATCGTCGCTACATACAAGACACTAAGACCTTGCTCGCCACGCGAGCAGCAAACATCGGCAGCAAAGCT
 6 Orf_virus_NZ2                                    98.5%  88.2%       AGCTCAACTTCTATCGTCGCTACATACAAGACACTAAGACATTGCTCGCCACGCGAGCAGCAAACATCGGCAGCAAAGCT
 7 Orf_virus_YX                                     98.1%  95.5%       AGCTCAACTTCTATCGTCGCTACATACAAGACACTAAGACACTACTCGCCACGCGAGCAGCAAACATCGGCAGCAAAGCT
 8 Orf_virus_GO                                     98.8%  96.4%       AGCTCAACTTCTATCGTCGCTACATACAAGACACTAAGACACTACTCGCCACGCGAGCAGCAAACATCGGCAGCAAAGCT
 9 Orf_virus_B029                                   91.8%  81.7%       AGCTCAACTTCTATCGTCGCTACATACAAGACACTAAGACATTGCTCGCCACGCGAGCAGCAAACATCGGCAGCAAAGCT
10 Orf_virus_IHUMI-1                                78.5%  70.9%       AGCTCAACTTCTATCGTCGCTACATACAAGACACTAAGACATTGCTCGCCACGCGAGCAGCAAACATCGGCAGCAAAGCT
11 Orf_virus_SJ1                                    77.2%  74.2%       AGCTCAACTTCTATCGTCGCTACATACAAGACACTAAGACACTACTCGCCACGCGAGCAGCAAACATTGGCAGCAAAGCT
12 Orf_virus_NP                                     24.1%  23.5%       AGCTCAACTTCTATCGTCGCTACATACAAGACACTAAGACACTACTCGCCACGCGAGCAGCAAACATCGGCAGCAAAGCT
   consensus/100%                                                      AGCTCAACTTCTATCGTCGCTACATACAAGACACTAAGACssTuCTCGCCACGCGAGCAGCAAACATsGGCAuCAAAGCT
   consensus/90%                                                       AGCTCAACTTCTATCGTCGCTACATACAAGACACTAAGACAsTuCTCGCCACGCGAGCAGCAAACATCGGCAGCAAAGCT
   consensus/80%                                                       AGCTCAACTTCTATCGTCGCTACATACAAGACACTAAGACAsTuCTCGCCACGCGAGCAGCAAACATCGGCAGCAAAGCT
   consensus/70%                                                       AGCTCAACTTCTATCGTCGCTACATACAAGACACTAAGACAsTuCTCGCCACGCGAGCAGCAAACATCGGCAGCAAAGCT

                                                     cov    pid  7201          .         .         .         .         :         .         .         . 7280
 1 Orf_virus_OV-SA00_NC_005336.1_115015-122053     100.0% 100.0%       CTGATCTACACCAACGACTACAACAACAGTGGCAACGTCGGCGAAGAGGAGCACTGCTCGGAGGAGTGCTGCAAAGTGGA
 2 Orf_virus_D1701                                  98.3%  88.3%       CTGATCTACACCAACGACTACAACGACAGTGGCAACGTCTGTGAAAAGGAGCACTGCTCGGAGGAGTGCTGCAGAGTGGA
 3 Orf_virus_NA1-11                                 97.9%  87.5%       CTGATCTACACCGACGACTACAACGACAGTGGCAACGTCGGCGAAGGGGAGCACTGCTCGGAGGAGTGCTGCAAAGTGGA
 4 Orf_virus_OV-HN3_12                              98.1%  87.2%       CTGATCTACACCGACGACTACAACGACAGTGGCAACGTCGGCGAAGGGGAGCACTGCTCGGAGGAGTGCTGCAAAGTGGA
 5 Orf_virus_OV-IA82                                98.6%  88.5%       CTGATCTACACCGACGACTACAACGGCAGTGGCAACGTCGGCGAAGGGGAGCACTGCTCGGAGGAGTGCTGCAAAGTGGA
 6 Orf_virus_NZ2                                    98.5%  88.2%       CTGATCTACACCGACGACTACAACGGCAGTGGCGACGTCGGCGAAAAGGAGCACTGCTCGGAGGAGTGCTGCAAAGTGGA
```

```
 7 Orf_virus_YX                                  98.1%  95.5%       CTGATCTACACCAACGACTACAACAACAGTGGCAACGTCGGCGAAGAGGAGCACTGCTCGGAGGAGTGCTGCAAAGTGGA
 8 Orf_virus_GO                                  98.8%  96.4%       CTGATCTACACCAACGACTACAACAACAGTGGCAACGTCGGCGAAGAGGAGCACTGCTCGGAGGAGTGCTGCAAAGTGGA
 9 Orf_virus_B029                                91.8%  81.7%       CTGATCTACACCGACGACTACAACGGCAGTGGCAACGTCGGCGAAGGGGAGCACTGCTCAGAGGAGTGCTGCAAAGTGGA
10 Orf_virus_IHUMI-1                             78.5%  70.9%       CTGATCTACACCGACGACTACAACGGCAGTGGCGACGTCGGCGAAAAGGAGCACTGCTCGGAGGAGTGCTGCAAAGTGGA
11 Orf_virus_SJ1                                 77.2%  74.2%       CTGATCTACACCAACGACTACAACAACAGTGGCAACGTCGGCGAAGAGGAGCACTGCTCGGAGGAGTGCTGCAAAGTGGA
12 Orf_virus_NP                                  24.1%  23.5%       CTGATCTACACCAACGACTACAACAACAGTGGCAACGTCGGCGAAGAGGAGCACTGCTCGGAGGAGTGCTGCAAAGTGGA
   consensus/100%                                                   CTGATCTACACCuACGACTACAACuuCAGTGGCuACGTCsGsGAAuuGGAGCACTGCTCuGAGGAGTGCTGCAuAGTGGA
   consensus/90%                                                    CTGATCTACACCuACGACTACAACuuCAGTGGCuACGTCGGCGAAuuGGAGCACTGCTCGGAGGAGTGCTGCAAAGTGGA
   consensus/80%                                                    CTGATCTACACCuACGACTACAACuuCAGTGGCAACGTCGGCGAAuuGGAGCACTGCTCGGAGGAGTGCTGCAAAGTGGA
   consensus/70%                                                    CTGATCTACACCuACGACTACAACuuCAGTGGCAACGTCGGCGAAGuGGAGCACTGCTCGGAGGAGTGCTGCAAAGTGGA

                                                   cov    pid 7281          .         3         .         .         .         .        :] 7351
 1 Orf_virus_OV-SA00_NC_005336.1_115015-122053 100.0% 100.0%       GGAAGTTCTGTGAGAAAGCGTGTTTTTCTGTAATGTGAAATAAGAA--GCCTTATGTGTGCACAGACATGG
 2 Orf_virus_D1701                               98.3%  88.3%       GGAAGTTCTGTGAGAAAGTGCGTTTTTCTGTAATGTGAAATAAGATAGGCCTTATGTGTGCACAAACATGG
 3 Orf_virus_NA1-11                              97.9%  87.5%       GGAAGTTCTGTGAGAAAGTGCGTTTTTCTGTAATGTGAAATAAGATA-GCCTTATGTGTGCACAGACATGG
 4 Orf_virus_OV-HN3_12                           98.1%  87.2%       GGAAGTTCTGTGAGAAAGTGCGTTTTTCTGTAATGTGAAATAAGATA-GCCTTATGTGTGCACAGACATGG
 5 Orf_virus_OV-IA82                             98.6%  88.5%       GGAAGTTCTGTGAGAAAGTGCGTTTTTCTGTAATGTGAAATAAGATA-GCCTTATGTGTGCACAGACATGG
 6 Orf_virus_NZ2                                 98.5%  88.2%       GGAAGTTCTGTGAGAAAGTGCGTTTTTCTGTAATGTGAAATAAGATA-GCCTTATGTGTGCACAGACATGG
 7 Orf_virus_YX                                  98.1%  95.5%       GGAAGTTCTGTGAGAAAGCGCGTTTTTCTGTAATGTGAAATAAGAA--GCCTTATGTGTGCACAGACATGG
 8 Orf_virus_GO                                  98.8%  96.4%       GGAAGTTCTGTGAGAAAGCGCGTTTTTCTGTAATGTGAAATAAGAA--GCCTTATGTGTGCACAGACATGG
 9 Orf_virus_B029                                91.8%  81.7%       GGAAGTTCTGTGAGAAAGTGCGTTTTTCTGTAATGTGAAATAAGATA-GCCTTATGTGTGCACAGACATGG
10 Orf_virus_IHUMI-1                             78.5%  70.9%       GGAAGTTCTGTGAGAAAGTGCGTTTTTCTGTAATGTGAAATAAGATA-GCCTTATGTGTGCACAGACATGG
11 Orf_virus_SJ1                                 77.2%  74.2%       GGAAGTTCTGTGAGAAAGCGCGTTTTTCTGTAATGTGAAATAAGAA--GCCTTATGTGTGAACAGACATGG
12 Orf_virus_NP                                  24.1%  23.5%       GGAAGTTCTGTGAGAAAGCGCGTTTTTCTGTAATGTGAAATAAGAA--GCCTTATGTGTGCACAGACATGG
   consensus/100%                                                   GGAAGTTCTGTGAGAAAGsGsGTTTTTCTGTAATGTGAAATAAGAs..GCCTTATGTGTGsACAuACATGG
   consensus/90%                                                    GGAAGTTCTGTGAGAAAGsGCGTTTTTCTGTAATGTGAAATAAGAs..GCCTTATGTGTGCACAGACATGG
   consensus/80%                                                    GGAAGTTCTGTGAGAAAGsGCGTTTTTCTGTAATGTGAAATAAGAs..GCCTTATGTGTGCACAGACATGG
   consensus/70%                                                    GGAAGTTCTGTGAGAAAGsGCGTTTTTCTGTAATGTGAAATAAGAs..GCCTTATGTGTGCACAGACATGG
```

MView (https://desmid.github.io/mview/) 1.63, Copyright © 1997-2018 Nigel P. Brown (mailto:biomview@gmail.com)
